# Optimization of strains for fermentation of kiwifruit juice and effects of mono- and mixed culture fermentation on its sensory and aroma profiles

**DOI:** 10.1016/j.fochx.2023.100595

**Published:** 2023-02-04

**Authors:** Tian Lan, Xinran Lv, Qinyu Zhao, Yushan Lei, Chenxu Gao, Quyu Yuan, Xiangyu Sun, Xuebo Liu, Tingting Ma

**Affiliations:** a College of Food Science and Engineering, College of Enology, Northwest A&F University, Yangling 712100, China; b Shaanxi Rural Science and Technology Development Center, Xi’an 710054, China; c Shaanxi Bairui Kiwifruit Research Co, Ltd, Xi’an 710054, China

**Keywords:** Kiwifruit juice, Fermentation, Strain screening, Volatile metabonomics, Aroma profile

## Abstract

In this study, a quality evaluation model of fermented kiwifruit juice (KJ) based on strain growth characteristics, sensory quality and functional characteristics was established by PCA, and the effects of mono- and mixed culture fermentation on the sensory and aroma profiles of KJ were comparatively studied. Experiments determined that *L. brevis* (LB) was the optimal strain for monoculture fermentation, and *L. plantarum* (LP2):LB = 1:2 was the optimum ratio for mixed fermentation. The results showed that lactic acid bacteria (LAB) fermentation significantly reduced the pH, soluble solid content and lightness, and improved its functional characteristics and viscosity. Mixed culture fermentation was superior to monoculture fermentation in terms of colony counts, sensory quality and viscosity. In general, after LAB fermentation, the concentrations of esters, ketones, alcohols and terpenoids in KJ increased significantly, while the concentrations of aldehydes decreased significantly. The production of esters and terpenoids was more strongly promoted by monoculture fermentation, while mixed culture fermentation promoted the production of more ketones and alcohols. 2,5-octanedione and 1-octen-3-ol could be the characteristic aroma compounds of mixed fermented KJ.

## Highlights

• *L. brevis* and *L. plantarum* were optimized as strains for fermenting kiwi juice (KJ).
• Mixed fermentation showed better colony counts, overall sensory score, and viscosity.
• Monoculture fermentation promoted the production of more esters and terpenoids in KJ.
• Mixed-culture fermentation promoted the production of more ketones and alcohols in KJ.
• 2,5-Octanedione and 1-octen-3-ol were characteristic aromas of mixed fermented KJ.

## Graphical abstract

**Figure:**
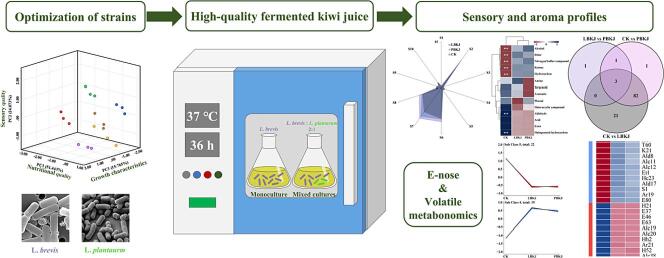

## 1 Introduction

As one of the most commercially valuable fruits, kiwifruit (*Actinidia* spp.) is highly favored by consumers for its unique flavor and rich nutrient content. Kiwifruit is rich in bioactive phytochemicals and has many health benefits (Wang et al., 2022). However, as a climacteric fruit, kiwifruit is not suitable for long-term storage after the eating-ripe stage and is prone to rotting and deterioration. Thus, vigorously developing deeply processed kiwifruit products, especially high-quality, nutritious, and healthy products, is an effective way to solve the decaying and wasting of fresh fruit and increase its added value and commercial income (Zhao et al., 2020). Currently, kiwifruit is processed into products such as jam, juice, wine, and vinegar. Among them, kiwifruit juice (KJ) products can greatly retain the flavor and nutrients of fresh kiwifruit and are becoming more popular among consumers (Ma et al., 2019). As more consumers pursue a nutritious and healthy lifestyle, it is necessary to explore novel processing techniques to enrich juice products and improve the health benefits of kiwifruit products.

Probiotic fermented fruit juice, as an emerging product, have received extensive attention for their rich nutritional and health benefits (Szutowska, 2020). The specific enzyme combinations in probiotics can metabolize various compounds in fruits in certain ways (Filannino et al., 2018), and then produce new active polysaccharides, organic acids, short-chain fatty acids and phenolic compounds, while reducing the content of sugar and anti-nutritional factors, such as alkaloids, tannins, oxalates, etc. (Emmanuel & Deborah, 2018). These transformations greatly change the functional table components of fruit juice, and improve the bioaccessibility and bioavailability of functional components to a certain extent (Cele et al., 2022), thus providing a variety of health benefits for the human body, such as enhanced gastrointestinal and immune function and reduced blood cholesterol and intestinal inflammation (Zhong et al., 2021). Meanwhile, probiotic fermentation can affect the aroma profile and sensory quality of fruit juice by producing and metabolizing volatile compounds, such as esters, alcohols, aldehydes, ketones, terpenes, etc. In the study of the influence of probiotics fermentation on the sensory characteristics of apple juice (Chen et al., 2019), passion fruit juice (Fonseca et al., 2022), jujube pulp (Pan et al., 2022) and grape juice (Sheng et al., 2022), probiotics fermentation has a certain beneficial impact on the aroma characteristics of fruit juice, and to a certain extent retains and enriches some typical aroma components of fruit juice, while producing some volatile substances with pleasant smell. Overall, probiotic fermentation is a process of lowering sugar, producing acid and forming a variety of secondary metabolites, which positively impacts the flavor, nutrition, functional characteristics, and shelf life of fruit and vegetable juices. Lactic acid bacteria (LAB) is a common probiotic used for this purpose in the food and beverage industry (Chen et al., 2019).

Recently, with the increasing demand for functional foods, research on the use of probiotics in processing fruit juice is increasing. Currently, several researchers have explored the fermentation of kiwifruit pulp (KP) (Chen et al., 2021, Zhou et al., 2020) and juice (KJ) (Wang et al., 2022). In the studies of Zhou et al., 2020, Chen et al., 2021, three LAB were used to ferment KP, and the transformation of phenolic compounds and *in vitro* digestion characteristics during fermentation were explored. Wang et al. (2022) used *Lactobacillus plantarum* and *L. acidophilus* to ferment juice from Xuxiang and Hongyang kiwifruit, respectively, and preliminarily explored the changes in bioactive substances and aroma components during fermentation. These studies indicated that fermentation by LAB exerts a positive effect on the flavor of KP/KJ, and the bioactive compound content and antioxidant activity were significantly improved after fermentation. Meanwhile, different LAB strains show significant differences in kiwifruit matrices of different cultivars. However, the existing studies were all carried out using monoculture fermentation conditions, compared with monoculture fermentation, mixed fermentation, as a more complex system, allows the exchange of multiple metabolites and provides mutual growth-stimulating effects (Pan et al., 2022). In the studies of apricot and grape juices fermentation, it was found that mixed fermentation showed better growth dynamic properties than monoculture fermentation, and it made the juice form more complex aroma compounds and was more advantageous in improving the aroma characteristics of the juice (Bujna et al., 2018, Sheng et al., 2022). At present, studies on the fermentation of KJ are limited to the effect of individual strains on a few kiwifruit cultivars; there is a lack of extensive research on fermentation by multiple and mixed strains, as well as a comprehensive study on the sensory qualities of fermented KJ.

In this study, Ruiyu (*Actinidia deliciosa* cv. Ruiyu) KJ was used as the matrix, and based on the fermentation properties, functional characteristics and sensory characteristics, LAB strains that were more suitable for KJ fermentation were screened from 6 commercial LAB for mono- and mixed culture fermentation. Colorimeter, electronic nose (E-nose), and volatile metabonomics were used to compare the effects of mono- and mixed culture fermentation on the sensory qualities of KJ, and the results are expected to provide a theoretical basis and technical reference to produce high-quality fermented KJ.

## 2 Materials and methods

### 2.1 Microorganism and inoculum preparation

*L. casei* CICC 20994 (LC), *L. pentosus* CICC 22174 (LP1), *L. plantarum* CICC 20265 (LP2), *L. brevis* CICC 20269 (LB) and *L. fermentum* CICC 21800 (LF) in lyophilized form were purchased from the China Center of Industrial Culture Collection (CICC, Beijing, China). *L. hamnosus* ATCC 53103 (LH) in lyophilized form was purchased from the American Type Culture Collection (ATCC, Manassas, VA, USA). All strains were activated in MRS broth (Land Bridge Technology Co., Ltd., Beijing, China). Sterilized glycerol (1:1, v/v) was added to the culture before storage at −80 °C.

Before use, all strains were activated in MRS broth at 37 °C for 12 h. The cells were collected by centrifugation at 2000×*g* for 5 min at 4 °C. The strain was resuspended into 7 mL of sterile normal saline to obtain a standby bacterial suspension.

### 2.2 Fermentation of KJ

**Fig. 1:**
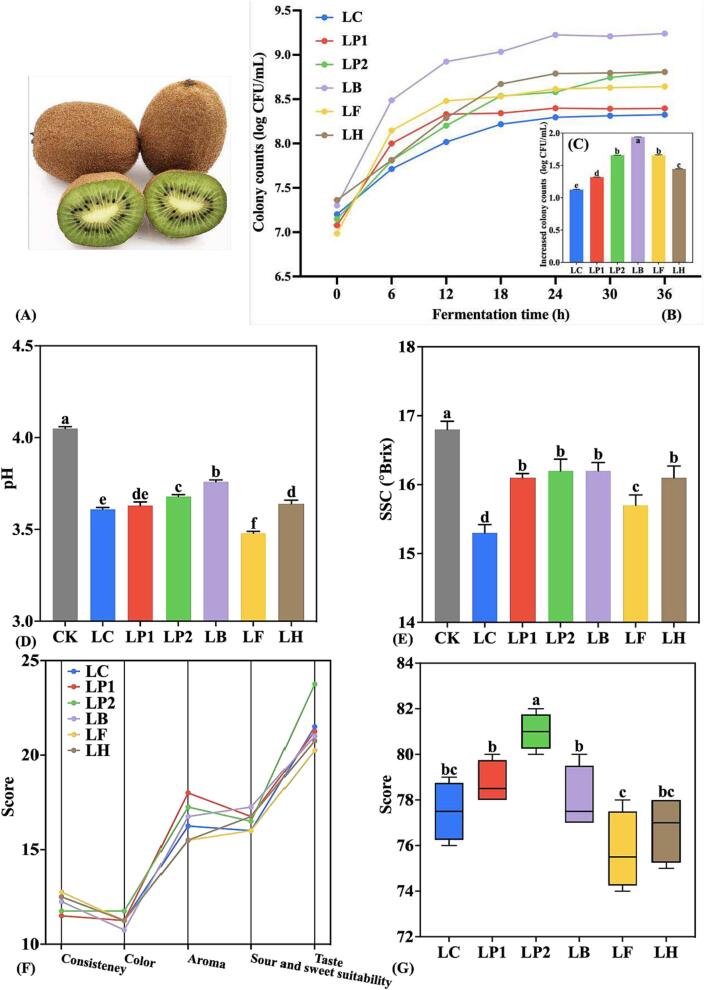
(A) The picture of Ruiyu; (B) growth curves and (C) increased colony counts of various LAB strains in KJ; (D) pH, (E) SSC, (F) sensory score and (G) overall sensory score of KJ samples. Lowercase letters indicate significant differences (*p* < 0.05) among KJ samples. LC, *L. casei* CICC 20994; LP1, *L. pentosus* CICC 22174; LP2, *L. plantarum* CICC 20265; LB, *L. brevis* CICC 20269; LF, *L. fermentum* CICC 21800; LH, *L. hamnosus* ATCC 53103; CK, control.

Ruiyu kiwifruits (Fig. 1 (A)) were purchased from Shaanxi Bairui Kiwifruit Research Co., Ltd. (Xi’an, China) and stored at room temperature until the eating-ripe stage for the KJ preparation. Kiwifruits were peeled and squeezed, and the pulp was centrifuged at 6000×*g* for 20 min to get KJ. Then the KJ was placed in a boiling water bath for 1 min to sterilize it, and it was cooled to about 40 °C before fermentation. The sterilized KJ complied with the microbiological safety criteria specified in the Chinese national standard GB 7101–2015; the pH was 4 ± 0.1 and the soluble solid content (SSC) was 16.5 ± 0.5°Brix.

LAB was inoculated in sterilized KJ so that the initial cell concentration in the KJ was about 7.0 log CFU/mL, and the accurate cell concentration was determined by OD600 (see Section 2.3). Fermented KJ was obtained by fermentation at 37 °C for 36 h. KJ cultured under the same conditions but without LAB added was used as the control check (CK).

### 2.3 Determination of colony counts

Optical density (OD600) linear regression models were established by referring to the method of Markkinen et al. (2022), that is, by taking the colony counts obtained by plate counting as the abscissa and OD600 values as the ordinate. The colony counts during KJ fermentation were monitored by OD600.

For this process, 5 mL KJ samples were centrifuged at 2,000 × *g* for 5 min at 4 °C, then the supernatant was discarded. The precipitation was resuspended into sterile normal saline to obtain the suspension, and the OD600 of the suspension was measured spectrophotometrically. The CK resuspended by the same centrifugation was used as a blank.

### 2.4 SSC and pH analysis

The SSC of KJ samples was determined using a PAL-1 Abbe Refractometer (Atago Co., Tokyo, Japan). The pH was determined using a PHS-3E pH meter (Shanghai Leici Co. ltd., Shanghai, China).

### 2.5 Ascorbic acid, total polyphenol, and total flavonoid analysis

The ascorbic acid content (AAC) of the juice samples was measured using the 2,6-dichloroindophenol titrimetric method. The total polyphenol content (TPC) and total flavonoid content (TFC) were determined by the Folin–Ciocalteu and AlCl_3_ colorimetric methods, respectively. The results were expressed as mg gallic acid equivalent (GAE)/L and mg catechol equivalent (CE)/L, respectively (Ma et al., 2019).

### 2.6 Sensory evaluation

The sensory evaluation procedure was approved by the local ethics committee. The sensory characteristics of juice samples were evaluated according to Pan et al. (2022) with slight modifications. The sensory panel was made up of 15 panelists who were experienced with sensory evaluation, 5 males and 10 females aged 20–40 years from Northwest A&F University. For the evaluation, 20 mL fermented KJ samples in transparent tasting cups were randomly coded and given to the panelists. Based on a 100-point scoring standard, the panelists evaluated the samples on 5 aspects: consistency, color, aroma, sour and sweet suitability, and taste, and the overall sensory score was the sum of the 5 indicators. The scoring details are shown in Table S1.

### 2.7 Establishment of quality evaluation model for fermentation KJ

The principal component analysis (PCA) was used to establish the quality evaluation model of fermentation KJ based on the fermentation characteristics of strains (increased colony count), sensory characteristics of KJ (overall sensory score) and functional characteristics of KJ (AAC, TPC and TFC), so as to obtain the comprehensive score of different strains and sort them.

The purpose of PCA was to convert many original variables into new variables, namely principal components (PCs) which were linear combinations of the original variables X_1_, X_2_, …, X_p_. In the majority of cases, two or three PCs were sufficient to explain the changes of about 80–90 % in the original variables, resulting in a large compression of data. If each sample was characterized with p variables Xi (columns), then PC Z_i_ could be presented as follows (Vainionpää et al., 2004):

(1) Z_i_ = a_i1_X_1_ + a_i2_X_2_ + a_i3_X_3_+…+ a_ip_X_p_

where a_ij_ were the component coefficient corresponding the eigenvalue.

The comprehensive evaluation function (F) was obtained by taking the variance contribution rate b_i_ of the PCs as the weight:

(2) F = b_1_Z_1_ + b_2_Z_2_ + b_3_Z_3_+…+b_p_Z_p_

### 2.8 Viscosity and color analysis

The viscosity of KJ samples was measured by an NDJ-5S rotary viscometer (Shanghai Pingxuan Co. Ltd., Shanghai, China). The color characteristics were determined with an X-Rite Ci7600 colorimeter (Grand Rapids, MI, USA) in transmission mode with CK as a control group. The lightness (L*), green/red component (a*), yellow/blue component (b*), chroma (C*), hue (h°), and total color difference (ΔE) of samples were recorded. The samples were tested in triplicate.

### 2.9 E-nose analysis

The PEN 3 E-nose (Airsense Analytics, Schwerin, Germany) was used to evaluate the overall odor profile of the juice samples. For this evaluation, 3 mL juice samples were put into 20 mL headspace bottles and equilibrated at 25 °C for 10 min before the test. The E-nose’s detection parameters were set by referring to Lan et al. (2021).

### 2.10 Volatile organic compound (VOC) analysis

A metabonomic strategy based on headspace–solid phase microextraction gas chromatography–mass spectrometry (HS-SPME/GC–MS) was used to determine VOCs in KJ samples. The extraction, detection, identification, and quantification of VOCs were performed by Wuhan MetWare Biotechnology Co., ltd. (www.MetWare.cn) as previously described (Xia et al., 2021). See supplementary materials for details.

Significantly differential volatile metabolites (DVMs) were screened based on the following conditions: fold change (FC) ≥ 2 or ≤ 0.5, variable influence on projection (VIP) ≥ 1, and p-value < 0.05 (Xia et al., 2021). All biological replicates per sample were analyzed in triplicate. A balanced mixture of all sample extractions (including 3 replicates) was settled as a mixed sample for quality control (QC).

### 2.11 Statistical analysis

Excel 16.4 was adopted to systematically sort out, analyze, and visualize the data. SPSS 26.0 (IBM, Armonk, NY, USA) was used for ANOVA, multiple comparisons, and principal component analysis (PCA). GraphPad Prism 9.3.1 was implemented for PCA and data visualization. Metabolomics data analysis was performed using the Metware Cloud, a free online platform for data analysis (https://cloud.metware.cn), and Adobe Illustrator 2020 was used to draw figures.

## 3 Results and discussion

### 3.1 Effects of fermentation by different LAB strains on KJ quality

#### 3.1.1 Bacterial growth profile during monoculture fermentation

The growth curves of 6 LAB strains in KJ during fermentation are shown in Fig. 1 (B). The colony counts of all strains remained at more than 8.00 log CFU/mL at the end of fermentation, which was in line with the minimum viability level of probiotics that may have a beneficial impact on host health after passing through the digestive tract, i.e., 10^6^-10^7^ CFU/mL or CFU/g of carrier food product (Szutowska, 2020). However, different LAB strains had different growth abilities in KJ. Among them, LB showed the strongest adaptability, which increased by 1.94 log CFU/mL at the end of fermentation, reaching the highest colony count of 9.20 log CFU/mL (Fig. 1 (B) & (C)). This might be due to the better adaptability of LB at lower pH conditions (Gong et al., 2019). LB was followed by LP2 and LF, both of which increased by 1.66 log CFU/mL during the 36 h of fermentation (Fig. 1 (C)). LP2 has been shown to have strong adaptability in various juice matrices and has shown good fermentation characteristics (Chen et al., 2019, Hashemi et al., 2017). However, LC only increased by 1.12 log CFU/mL after 36 h of fermentation. Studies have shown that LC has a strong acid-producing ability and will rapidly reduce the pH of juice after fermentation, thereby inhibiting the growth and reproduction of bacteria and ending fermentation early (Chen et al., 2019). In addition, all LAB strains increased rapidly during the first 24 h of fermentation, then reached a stable period; the reason for this phenomenon might be that LAB produce large amounts of acid, which inhibits the proliferation of bacteria while using nutrients to grow and reproduce.

#### 3.1.2 Effect of fermentation with different LAB on pH, SSC and sensory quality of KJ

The effects of different LAB strains on the physicochemical and sensory properties of KJ are shown in Fig. 1(D-G). According to Fig. 1(D) & (E), compared with CK, the pH and SSC of all fermented KJ samples were significantly reduced (*p* < 0.05). Among them, LB fermentation decreased pH and SSC by only 6.0 % and 3.0 % respectively, while LF fermentation decreased both by 13.0 % and 6.0 %. This directly affected the sensory scores in sour and sweet suitability; that is, LB scored the highest and LF scored the lowest (Fig. 1 (F)).

Further sensory evaluation of fermented KJ was carried out (Fig. 1 (F)), and the overall sensory score was obtained (Fig. 1 (G)). The results show that KJ fermented by LP2 performed best in color and taste, while KJ fermented by LB had the highest score in sour and sweet suitability, but the lowest in color. In addition, KJ fermented by LP1 scored highest in aroma, but lowest in consistency. In general, KJ fermented by LP2 had the highest overall sensory score, significantly higher than the other LAB (*p* < 0.05), followed by LP1 and LB, but there was no significant difference in the overall sensory score between the two (*p* > 0.05).

#### 3.1.3 Effect of fermentation by different LAB on functional components of KJ

**Fig. 2:**
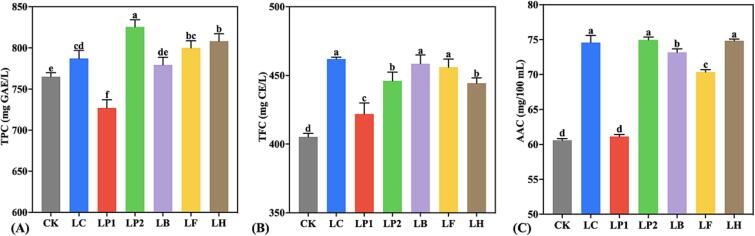
(A) TPC, (B) TFC and (C) AAC of KJ samples. Lowercase letters indicate significant differences (*p* < 0.05) among samples.

As shown in Fig. 2(A), compared with CK, the TPC of KJ was significantly increased after fermentation by most LAB strains (except LP1 and LB). This might be due to the fact that during the fermentation process, complex phenolics are converted to more simple phenols through glycosyl hydrolase, phenolic acid decarboxylase and reductase, and esterase activities (Filannino et al., 2018). At the same time, compared with other LAB strains, LP2 more strongly enhanced TPC, with an increase of about 60 mg GAE/L, which might be because LP2 has the ability to deglycosylate glycosylated phenolics and release soluble conjugated and insoluble bound phenolic fractions from plant cell walls (Li et al., 2021). In a study of fermented mulberry juice by Kwaw et al. (2018), LP2 also showed an excellent ability to increase the concentration of phenolics. Moreover, some studies have shown that phenolic hydroxyl in phenolic acid structure can protect probiotics, so the increase of TPC might promote the proliferation of LAB to a certain extent (Zhang et al., 2023). As shown in Fig. 2(B), TFC enhanced significantly in KJ after LAB fermentation (*p* < 0.05), a result consistent with that obtained by Wang et al. (2022). This was mainly attributed to during fermentation, the production of hydrolytic enzymes contributes to the hydrolyzation of complex phenolics to simpler forms, thereby increasing the concentration of flavonoids in the food matrix (Kwaw et al., 2018). Specifically, specific bacterial glycosyl hydrolases can convert flavonoid glycosides to the corresponding aglycones, which usually show higher bioactivity in humans than their precursor glycosides (Filannino et al., 2018). In addition, the difference in TFC among fermented KJ samples might be due to the individual adaptability of the strains and their ability to produce hydrolytic enzymes. Kiwifruit is known as the “king of vitamin C”, so the AAC of KJ is an important nutritional evaluation index (Ma et al., 2017). As shown in Fig. 2(C), except for LP1, the AAC in KJ after LAB fermentation was significantly higher than in CK (*p* < 0.05), and the AAC after fermentation by LC, LP2 and LH increased by 15–22 %. The occurrence of this phenomenon is more attributed to the high retention of ascorbic acid (AA) by LAB fermentation than to the production of AA. In general, AA is sensitive to temperature, pH, dissolved oxygen and metal ions during processing (Zhao et al., 2021), and the pH reduction and oxygen depletion caused by fermentation contribute to improving its stability (Hal et al., 2013). Therefore, compared with the KJ samples without inoculation but cultured under the same conditions (CK), LAB fermentation can retain the original AA in KJ to a large extent. In general, LAB fermentation significantly enhanced the TPC, TFC and AAC in KJ, thereby significantly improving its functional characteristics.

### 3.2 PCA-based preferential selection of strains for fermented KJ

**Fig. 3:**
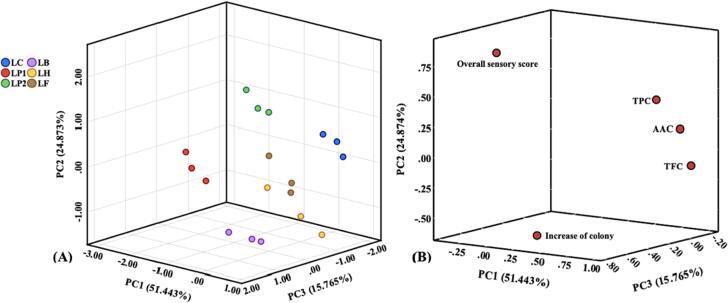
(A) PCA score plot and (B) loading plot of fermented KJ with six LAB strains.

The excellent strains were selected by the quality evaluation model of fermented KJ based on PCA. As shown in Fig. 3(A), three PCs were extracted, and their total variance explained is 92.083 %, which indicates that the three PCs can explain the original variables. According to Fig. 3(B) and Table S2, PC1 mainly explains the information about AAC, TPC and TFC, which reflects the functional characteristics, while PC2 and PC3 respectively explain the overall sensory score and increased colony counts, reflecting the sensory quality and growth characteristics. Based on the component coefficient matrix and standardized data, the score expression of the three PCs can be obtained as follows:

(3) Z_1_ = -0.103X_1_ + 0.141X_2_ + 0.373X_3_ + 0.310X_4_ + 0.351X_5_

(4) Z_2_ = 0.65X_1_-0.465X_2_ + 0.165X_3_ + 0.355X_4_-0.11X_5_

(5) Z_3_ = 0.60X_1_ + 0.925X_2_-0.019X_3_ + 0.053X_4_-0.222X_5_

where Z_1_-Z_3_ are the scores of the three PCs, and X_1_-X_5_ are the standardized data of increased colony count, overall sensory score, ACC, TPC and TFC.

Then, according to equation (2) and in combination with the above analysis, the comprehensive evaluation function (F), namely equation (6), was obtained, as follows:

(6) F = 0.559Z_1_ + 0.270Z_2_ + 0.171Z_3_

According to the quality evaluation model, the scores and ranking of KJ after fermentation with different LAB strains were calculated, and are shown in Table S3. The results show that KJ fermented by LP2 had the highest comprehensive score, followed by LB. Specifically, LP2 scored the highest in sensory quality and second in colony counts, while LB scored the highest in colony counts, and second in functional characteristics. Considering that the total enumeration of viable bacteria is a key metric in probiotic industrial science, used to ensure strain health benefits (Hansen et al., 2018), thereby the increased colony counts in KJ were taken as the first-order parameter. Based on this, LB was selected as the target strain for monoculture fermentation in subsequent studies. In addition, the sensory quality of juice is an important factor affecting consumer acceptance. Therefore, LP2, with the optimal sensory score, was selected as the compound strain with LB for subsequent mixed fermentation studies to improve the sensory quality of fermented KJ.

### 3.3 Determination of mixed cultures ratio of LAB

#### 3.3.1 Bacterial growth profile during mixed fermentation

**Fig. 4:**
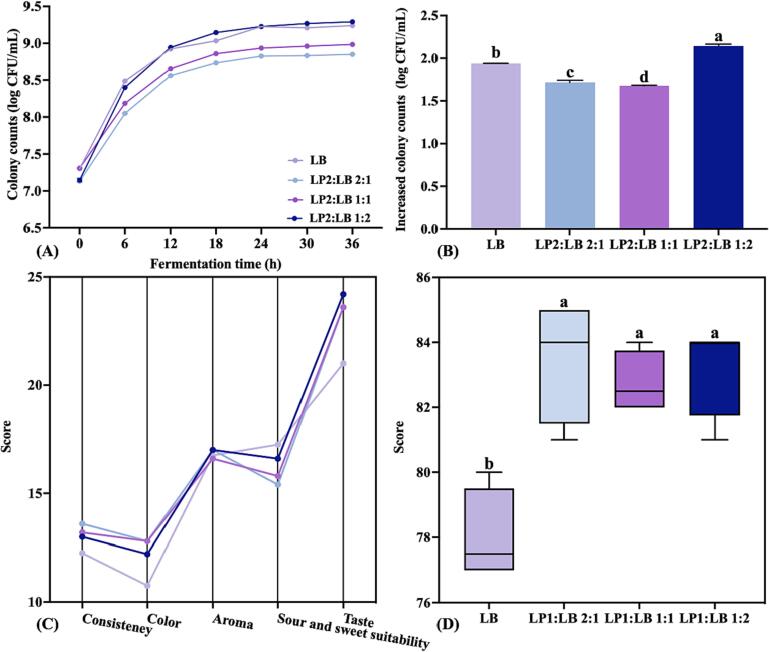
(A) Growth curves and (B) increased colony counts of different LAB ratios in KJ, and (C) sensory score and (D) overall sensory score of mixed fermented KJ with different LAB ratios. Lowercase letters indicate significant differences (*p* < 0.05) among samples.

As shown in Fig. 4(A), the ratio of LP2 to LB was set at 2:1, 1:1, and 1:2, and the initial cell concentration in KJ was about 7 log CFU/mL; KJ was fermented at 37 °C for 36 h, and establish growth curves. The results show that the growth characteristics of LAB in KJ at different ratios were significantly different. When the LP2:LB ratio was 1:2, the colony count reached the highest value of 9.29 log CFU/mL after fermentation, an increase of 2.14 log CFU/mL compared to pre-fermentation (Fig. 4(B)), which was significantly higher than LB fermentation (*p* < 0.05). When LP2:LB was 2:1 or 1:1, although the increased colony counts were higher than those of most monoculture fermentations, they were significantly lower than that of LB fermentation (*p* < 0.05). This indicates that the appropriate ratio of mixed fermentation could significantly improve the fermentation characteristics of KJ.

#### 3.3.2 Effect of different ratios of mixed fermentation on sensory quality of KJ

A sensory evaluation of fermented KJ with different mixed culture ratios was carried out (Fig. 4(C) & (D)). In terms of overall sensory quality, the scores for KJ with mixed fermentation were significantly higher compared to LB mono-fermentation (*p* < 0.05), which indicates that mixed fermentation could improve the sensory quality of KJ. However, there was no significant difference in the overall sensory scores of mixed culture fermented KJ with different ratios (*p >* 0.05). Notably, when LP2:LB was 1:2, the aroma, taste and sour and sweet suitability had better scores. Therefore, mixed fermentation with LP2:LB = 1:2, with the best fermentation characteristics and good sensory quality, was used for the subsequent comparative study.

### 3.4 Comparative study of the mono- and mixed culture fermented KJ

Based on the above results, LBKJ was obtained by monoculture fermentation with LB, and PBKJ was obtained by the mixed culture fermentation with LP2:LB = 1:2. Taking CK as the control, the sensory and aroma profiles of LBKJ and PBKJ were compared, and the effect of fermentation on the VOCs of KJ was further clarified based on the volatile metabolomics.

#### 3.4.1 Viscosity

**Fig. 5:**
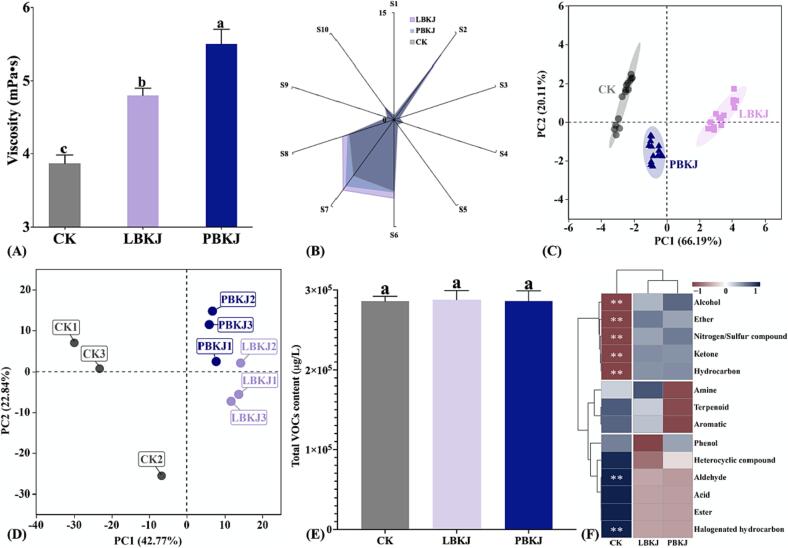
(A) Viscosity of KJ samples, with lowercase letters indicating significant differences (*p* < 0.05) among samples; (B) radar map and (C) PCA of overall odor profile of CK, LBKJ and PBKJ by E-nose; (D) PCA score plot for CK, LBKJ, and PBKJ based on results of volatile metabonomics; (E) TVC of KJ samples, with lowercase letters indicating significant differences (*p* < 0.05) among samples; and (F) heatmap of contents of different classes of VOCs in KJ samples; **: significant difference at *p* < 0.05; shifting shades from red to blue represent values changing from low to high. (For interpretation of the references to color in this figure legend, the reader is referred to the web version of this article.)

Fig. 5(A) shows the viscosity of different KJ samples. It can be seen that both mono- and mixed fermentation significantly increased the viscosity of KJ (*p* < 0.05), and compared with CK, the viscosity of PBKJ increased by 1.6 mPa·s, twice the increase of LBKJ. Increased viscosity can efficiently improve homogeneity and cloud stability and prevent sedimentation in juice (Aghajanzadeh et al., 2017). This phenomenon might be caused by the synthesis of exopolysaccharides (EPSs) by LAB during fermentation (Welman and Maddox, 2003).

#### 3.4.2 Color profile

Color is one of the most important sensory attributes of juice, and has an important effect on commercial acceptance. As shown in Table S4, compared with CK, the L*, a*, b* and C* values of fermented KJ were significantly lower and h° significantly higher, and the total color difference was distinguishable by the naked eye, i.e., ΔE > 2.0. The color difference between fermented KJ and CK is mainly due to the change in L*, and studies have shown that increased viscosity might reduce the lightness (Aghajanzadeh et al., 2017). In a study by Wang et al. (2022), it was also proved that LAB fermentation significantly reduced the L* value of KJ.

#### 3.4.3 Overall odor profile: E-nose analysis

The aroma of juice is an important factor affecting consumer preference (Zhang et al., 2019), and LAB fermentation can significantly improve the aroma profile and VOC formation in juice (Wang et al., 2020). In order to explore the effect of fermentation on the overall odor profile of KJ, an E-nose was used for detection in juice samples and the data of 54–59 s were selected for further analysis. It can be seen from Fig. 5(B) that the responses of the E-nose to fermented KJ generally increased compared with CK, with the most significant changes in sensors S2 (broad-range sensitivity, very sensitive to nitrogen oxides) and S7 (sensitive to many sulfur organic compounds and terpenes), followed by S6 (sensitive to methane, broad range) and S8 (alcohol, sensitive to aromatic compounds with broad range). In addition, the PCA results (Fig. 5(C)) showed the overall odor profiles of different KJ were significantly different and could be clearly distinguished by PC1.

#### 3.4.4 Aroma profile: volatile metabolome analysis

Differences in the types and concentrations of aroma components in juice result in different aroma and flavor profiles (Wu et al., 2022). In this study, the changes of VOCs in KJ before and after LAB fermentation were investigated by HS-SPME/GC–MS. According to the total ion chromatogram (TIC) diagram of the mixed samples and intragroup correlation analysis (Fig. S1), three samples of each group exhibited good data reproducibility (Pearson > 0.98) and stability. Furthermore, PCA was performed to analyze the credibility of the identification results and overall compound differences among nine samples (Fig. 5 (D)). The results show that PC1 explained 42.77 % of the total variance and separated the samples according to whether they were fermented or not, indicating that the VOCs of KJ changed significantly after fermentation, while PC2 explained 22.84 % of the total variance and distinguished LBKJ and PBKJ according to different fermentation strategies, indicating that different fermentation strategies also had an effect on the VOCs. A total of 628 VOCs were detected in all KJ samples, including 142 terpenoids (T), 92 heterocyclic compounds (Hc), 87 esters (E), 70 ketones (K), 56 hydrocarbons (H), 54 alcohols (Alc), 42 aldehydes (Ald), 41 aromatics (Ar), 13 acids (Ac), 10 nitrogen/sulfur compounds (N/S), 9 phenols (P), 9 amines (Am), 2 halogenated hydrocarbons (Hh), and one ether (Et) (Table S5).

As shown in Fig. 5 (E), there was no significant difference in total VOCs content (TVC) among CK, LBKJ and PBKJ (*p* > 0.05); thus, the changes in aroma profile were mainly reflected in the types and concentrations of VOCs in KJ. It can be seen in Table S5 that, in all KJ samples, the concentration of terpenoids was the highest, accounting for 36.95–38.23 % of TVC, followed by heterocyclic compounds (15.33–16.91 %), esters (11.35–11.83 %), alcohols (6.63–9.32 %), aldehydes (5.37–6.86 %), ketones (5.08–6.16 %) and hydrocarbons (4.97–6.16 %), while the other VOCs accounted for<3.5 % of TVC. As shown in According to Fig. 5 (F), among all types of VOCs, the content of alcohols, ether, nitrogen/sulfur components, ketones and hydrocarbons increased significantly after fermentation (*p* < 0.05), while the content of aldehydes and halogenated hydrocarbons decreased significantly (*p* < 0.05), and other types of VOCs had no significant change; in addition, the content of all types of VOCs showed no significant difference between LBKJ and PBKJ (*p* > 0.05). Therefore, the differential accumulation of specific VOCs is the main reason for the aroma changes during KJ fermentation. In order to understand the change differences in VOCs between mono- and mixed culture fermentation, the DVMs of different KJ samples were further analyzed.

**Fig. 6:**
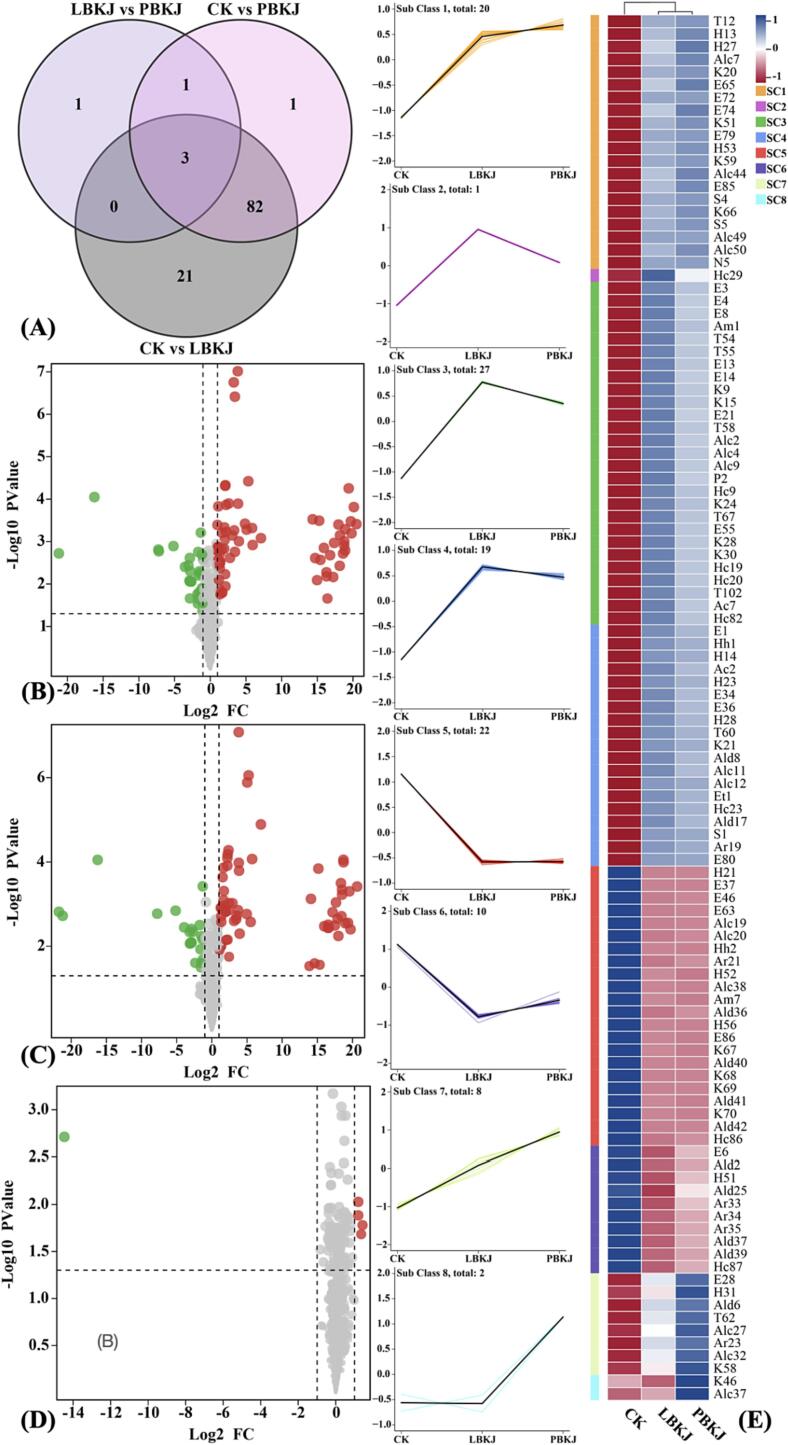
(A) Venn diagram of DVMs of CK vs LBKJ, CK vs PBKJ, and LBKJ vs PBKJ; (B–D) volcano plot of DVMs of CK vs LBKJ, CK vs PBKJ, and LBKJ vs PBKJ, with up-regulation in red, down-regulation in green; (E) line charts plot for K-means clustering analysis of DVMs and visualized in clustering heatmap, where shifting shades from red to blue represent values changing from low to high. (For interpretation of the references to color in this figure legend, the reader is referred to the web version of this article.)

Orthogonal projections to latent structure discriminant analysis (OPLS-DA) was applied to distinguish the DVMs (Fig. S2), and further, DVMs were screened. Overall, 109 VOCs exhibited differential metabolism, of which 78 were up-regulated and 32 were down-regulated (Fig. 6(A) & Table S6). Compared with CK, 74 DVMs were up-regulated and 32 DVMs were down-regulated in LBKJ (Fig. 6(B)), and 64 DVMs were up-regulated and 23 DVMs were down-regulated in PBKJ (Fig. 6(C)). In LBKJ vs PBKJ, 5 DVMs were up-regulated and one DVM was down-regulated. These DVMs might be important factors in altering the aroma profile during KJ fermentation. Therefore, these significantly up- and down-regulated DVMs were further analyzed.

The DVMs were clustered using the K-means clustering algorithm (Fig. 6(E)), and the DVMs of three KJ samples were divided into eight sub-classes (SCs) according to the accumulation patterns. The 67 kinds of DVMs in SC 1–4 were up-regulated after fermentation, among which 16 were mainly esters, followed by 10 ketones, 9 alcohols and 7 terpenoids. In contrast, 32 DVMs in SC 5–6 were down-regulated, among which there were 8 types of aldehydes, followed by 5 esters, 4 ketones, 4 hydrocarbons and 4 aromatics. The DVMs in SC 7 showed different degrees of increase in LBKJ and PBKJ; specifically, mixed fermentation significantly increased this class of VOCs compared to monoculture fermentation. In SC 8, 2,5-octanedione (K46) and 1-octen-3-ol (Alc37) showed no significant changes during monoculture fermentation but increased significantly after mixed fermentation. These data indicate that DVMs in SC 7–8 were closely related to the fermentation strategies. Studies have indicated that strain species and their metabolic variability in the food matrix are closely related to the formation of food aromas, and mono- and mixed cultures play different roles in changing the matrices, which may contribute to improving the aroma profile of foods (Fonseca et al., 2022).

Volatile ester compounds, the most abundant VOCs in fresh kiwifruit and the main source of fruity and sweet aromas (Lan et al., 2021). As shown in Table S6 and Fig. 6(E), there were 22 esters in DVMs. Among them, 15 esters were up-regulated and 4 esters were down-regulated in both LBKJ and PBKJ compared to CK. Additionally, (+)-*cis*-verbenol, acetate (E21) and 2-hydroxyphenyl benzoate (E55) were only up-regulated and methyl laurate (E6) was down-regulated only in LBKJ. However, no ester DVMs were observed in LBKJ vs PBKJ. LAB fermentation contributed to the formation of esters in KJ, producing a total of seven esters: ethyl butyrate (E85), bornyl butyrate (E3), γ-terpinyl acetate (E13), ethyl (E,Z)-2,4-decadienoate (E14), ethyl (E,Z)-2,4-decadienoate (E34), geranyl formate (E36) and nonyl acetate (E28). In terms of concentration, E85, E28 and ethyl phenylacetate (E65) were significantly higher in PBKJ than in LBKJ (*p* < 0.05), and conversely, E3, E14, E34, E36, neryl isobutyrate (E8) and (+)-*cis*-verbenol acetate (E21) were significantly lower in PBKJ than LBKJ (*p* < 0.05). The production or up-regulation of these esters mainly increased ripe-fruit aromas in fermented KJ. Only five ester DVMs were down-regulated in fermented KJ, among which phenyl propyl carbonate (E37) was metabolized completely and disappeared during fermentation, while 2-methyl butyl isovalerate (E46), heptyl acetate (E63), methyl 3- methyl-2-butenoate (E86) and methyl laurate (E6), with herbal and unripe fruit flavor, were not significantly different between LBKJ and PBKJ. In conclusion, LAB fermentation reduced unripe-fruit odor and enhanced ripe-fruit aroma, giving KJ more fruity and sweet flavors. Moreover, an increase in various esters has also been found in various fermented juices, such as fermented apple and passion fruit juice (Chen et al., 2019, Fonseca et al., 2022). In a word, LAB fermentation can promote the formation and production of esters to enrich the aroma of fermented KJ, providing a pleasant experience for consumers. In addition, the production of esters was found to be more complex in LBKJ than in PBKJ in this study.

Ketones have an intense aroma in low concentrations (Chen et al., 2019). After LAB fermentation, the concentration of ketones increased significantly in KJ (Fig. 5(F)). A total of 11 ketones were up-regulated (Table S6 & Fig. (E)), and only (±)-α-damascone (K15), 2-hydroxy-1-phenylbutan-1-one (K28) and 4-methoxyphenylacetone (K30) were up-regulated in LBKJ. The remaining eight ketone DVMs were up-regulated in both LBKJ and PBKJ, and among them, 2-heptanone (K66), piperitenone oxide (K24), and 2-octanone (K58) were produced during fermentation. While the concentrations of 3-(hydroxymethyl)-2-nonanone (K20), 8-nonen-2-one (K51), K66 and K58 were significantly higher in PBKJ than LBKJ (*p* < 0.05), these compounds contributed to fresh fruit and herb flavor. In contrast, K24 and 2-undecanone (K21), with herbal and fatty aromas, had significantly higher concentrations in LBKJ than PBKJ.

Notably, K58 and 2,5-octanedione (K46) showed up-regulation in LBKJ vs PBKJ, imparting herbal flavor to the juice. In addition, fermentation led to the down-regulation of three ketone DVMs, among which 2-hexanone (K68), with fruit and buttery odors, was completely metabolized and mesityl oxide (K69) and 4-hexen-3-one (K70), with a strong spicy odor, were largely degraded. This indicates that LAB fermentation promoted the production of a large number of ketones in KJ with fruity, sweet, herbal and creamy aromas while degrading some ketones with pungent odors. Moreover, compared with monoculture fermentation, mixed fermentation had a stronger promoting effect on the production of ketones.

Alcohols are an important class of VOCs in kiwifruit (Lan et al., 2021) that not only impart pleasant aromas to food, but also assist in the presentation of other aroma compounds (Chen et al., 2019). Compared with CK, the alcohol concentration in fermented KJ was significantly increased (Fig. 5(F)). 1-Hexanol (Alc49) was the most concentrated compound in fermented KJ (Table S6 & Fig. 6(E)) and was up-regulated after fermentation, which provided the juice with sweet and fruity aromas. (E)-2-hexen-1-ol (Alc50) was also up-regulated after fermentation and showed significantly higher concentrations in PBKJ than LBKJ, providing the fermented KJ with fresh fruit and leafy aromas. C6 alcohols are the core aroma compounds in fresh kiwifruit (Lan et al., 2021), therefore, an increase in these substances accentuates the characteristic odor of the kiwifruit in fermented KJ. In addition, the concentrations of 5-undecanol (Alc7), cyclooctanemethanol (Alc27), 1-octanol (Alc32) and 1-octen-3-ol (Alc37) in PBKJ were significantly higher in PBKJ than LBJK (*p* < 0.05), but only Alc37 showed up-regulation; these alcohols enrich the fruity, floral, and mushroom flavor of PBKJ. Three kinds of alcohol DVMs were down-regulated after fermentation: 3,7-dimethylocta-1,5,7-trien-3-ol (Alc19), 3,7-dimethyl-1,5(E),7-octatrien-3-ol (Alc20) and 2,6-dimethylcyclohexanol (Alc38), which were not significantly different in LBKJ and PBKJ, and their down-regulation reduced the musty and herbal odors in KJ. Overall, the increased alcohols in KJ after LAB fermentation brought out pleasant aromas of sweetness, fruitiness and freshness, while the small content of alcohols with undesirable odors was significantly reduced, which effectively improved the aroma profile of KJ.

Terpenoids are considered to provide floral, herbal and citrus aromas to foods (Ge et al., 2021). In this study, all terpenoid DVMs were up-regulated (Table S6 & Fig. 6(E)), among which β-sinensal (T12), *trans*-3a-*cis*-9a-1,2,3,3a,8,9,9a,9b-octahydro-4H-cyclopenta[*def*]phenanthrene (T54), perilla alcohol (T102) and *trans*-para-menthane-3,8-diol (T60) were produced during fermentation, providing the fermented KJ with orange, floral and herbal aromas. Meanwhile, T54, T102, T60 and perillic acid (T67) showed significantly higher concentrations in LBKJ than in PBKJ; on the contrary, diosphenol (T62), with mint and herbal aromas, had significantly higher concentrations in PBJK than in LBKJ. All of the above terpenoids were up-regulated in both CK vs LBKJ and CK vs PBKJ. However, geranyl acetate (T55) and (E)-β-damascenone (T58), the terpenoid DVMs with the highest concentrations, provided the KJ with floral, honey and apple aromas, and only showed up-regulation in CK vs LBKJ, but in terms of concentration, there was no significant difference between LBKJ and PBKJ (*p* > 0.05). In summary, the up-regulation of terpenoids such as geranyl acetate and damascenone during fermentation provided KJ with fruity, floral, honey, and herbal aromas, and this phenomenon was more prominent in LBKJ. Generally, this could improve the aroma profile of KJ to a certain extent, however, it is worth noting that terpenoids with higher concentration may cause off-flavor. The influence of terpenoids on the aroma characteristics of KJ will be further clarified based on sensory omics in the future.

In this study, aldehydes decreased significantly after LAB fermentation of KJ (Fig. 5(F)), which was also found in fermented jujube pulp (Pan et al., 2022). After LAB fermentation, eight aldehyde DVMs were down-regulated (Table S6 & Fig. 6(E)); only 3-ethylbenzaldehyde (Ald25), 4-heptenal (Ald37) and benzaldehyde (Ald39) were down-regulated in CK vs LBKJ, and the other five aldehyde DVMs were down-regulated in the comparison between CK and the two fermented KJ. Notably, C6 aldehydes hexanal (Ald40), (Z)-3-hexenal (Ald41) and (Z)-3-hexenal (Ald42), with herbal, fruity, and fatty aromas, were down-regulated after fermentation, which was consistent with the results of Wang et al. (2022) in fermented KJ. The reason for this phenomenon might be that aldehydes can be reduced to alcohols or oxidized to acids due to their instability under the action of microorganisms (Pan et al., 2022). However, as a kind of important aroma component in fresh kiwifruit, the down-regulation of C6 aldehydes could reduce the grass odor in KJ, but might also lead to the weakening of characteristic aroma in KJ.

In conclusion, the concentrations of esters, ketones, alcohols, and terpenoids in KJ increased significantly and the concentrations of aldehydes decreased significantly after LAB fermentation, which not only enriched the fruity, sweet, floral and herbal aromas in fermented KJ, but also reduced some of the unpleasant odors, thus improving the aroma profile to a certain extent. However, different fermentation strategies have different effects on the presentation of the aroma profile of KJ; specifically, monoculture fermentation had a more significant promoting effect on the production of esters and terpenoids, while mixed fermentation promoted the production of more ketones and alcohols. In terms of DVMs, K46 and Alc37 showed significant up-regulation only under mixed fermentation and their concentrations were significantly higher than in CK and LBKJ, indicating that they might be characteristic substances under mixed fermentation. In a word, LAB fermentation can effectively improve the aroma profile of KJ, and different fermentation strategies will have different effects, which is determined by the individual adaptability of the strain in the food matrix.

## 4 Conclusion

In this paper, the suitable strains for KJ fermentation were optimized and evaluated, and the effects of mono- and mixed culture fermentation on its sensory and aroma profiles were explored. Compared with CK, both mono- and mixed culture fermentation significantly increased the viscosity and decreased the lightness of KJ. However, mixed fermentation was superior to monoculture fermentation in terms of increased colony counts, overall sensory score, and viscosity. After fermentation, the odor characteristics were more obvious. In general, LAB fermentation significantly improved the aroma profile of KJ, enriching the fruity, sweet, floral and herbal aromas, while reducing some of the unpleasant odors. In terms of different fermentation strategies, the production of esters and terpenoids was more strongly promoted by monoculture fermentation, while mixed culture fermentation promoted the production of more ketones and alcohols. In the comparison between LBKJ and PBKJ, 2,5-octanedione and 1-octen-3-ol showed significant up-regulation only under mixed fermentation, indicating that they might be the characteristic substances under mixed fermentation. In conclusion, LAB fermentation is an effective way to improve the functional and sensory qualities of KJ, and the aroma profile can be effectively improved by monoculture fermentation of *L. brevis* and mixed culture fermentation of *L. plantarum* and *L. brevis*. In the future, we will focus on the change law of the nutritional quality and specific phytochemicals of KJ under different fermentation strategies, so as to provide more comprehensive information on fermented KJ.

## Funding

This work was supported by the class General Financial Grant from the China Postdoctoral Science Foundation (2020M673505), the Innovation Capacity Support Plan of Shaanxi Province (2022NY-039, 2022ZDLNY04-04, 2023-YBNY-176, 2020-TD-47).

## CRediT authorship contribution statement

**Tian Lan:** Methodology, Investigation, Data curation, Visualization, Writing – original draft. **Xinran Lv:** Methodology, Investigation, Data curation, Visualization, Writing – original draft. **Qinyu Zhao:** Methodology, Investigation, Data curation. **Yushan Lei:** Resources. **Chenxu Gao:** Methodology, Investigation, Data curation. **Quyu Yuan:** Methodology, Investigation, Data curation. **Xiangyu Sun:** Conceptualization, Writing – review & editing. **Xuebo Liu:** Conceptualization, Writing – review & editing. **Tingting Ma:** Conceptualization, Methodology, Writing – review & editing.

## Declaration of Competing Interest

The authors declare that they have no known competing financial interests or personal relationships that could have appeared to influence the work reported in this paper.

## Data Availability

Data will be made available on request.
