# Supplementary material for: Optimization of strains for fermentation of kiwifruit juice and effects of mono- and mixed culture fermentation on its sensory and aroma profiles
Source: Food Chem X. 2023 Feb 4;17:100595. doi: 10.1016/j.fochx.2023.100595 (PMC9941363; doi:10.1016/j.fochx.2023.100595)
Supplement: Supplementary data 1 [file mmc1.docx]

**
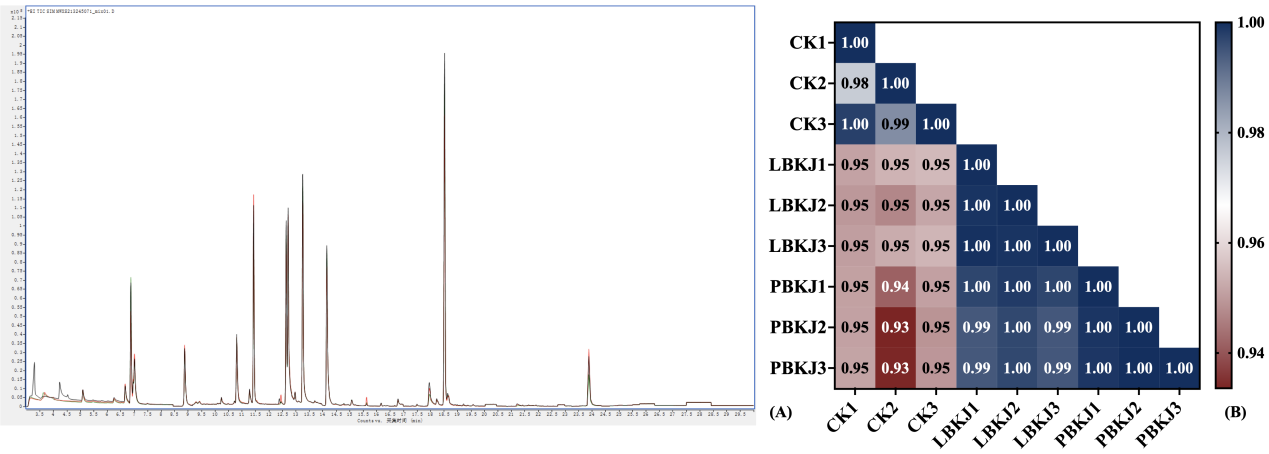
Fig. S1 The TIC diagram of the mixed samples (A) and intragroup correlation analysis (B).**

**
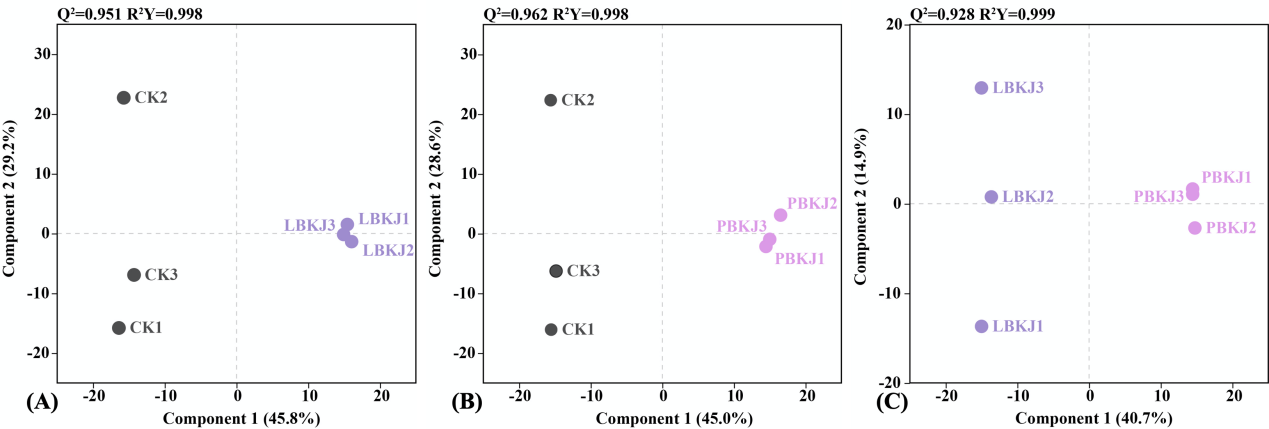
Fig. S2 OPLS-DA between CK and LBKJ (A), CK and PBKJ (B), LBKJ and PBKJ (C).**

**Table S1 Sensory attributes and reference scores used for sensory evaluation of KJ**

| Sensory attributes | Standard | Score |
| --- | --- | --- |
| Consistency  (15 points) | No suspended solids or precipitation in juice, and uniform | 12～15 |
|  | A small amount of suspended solids or precipitation in juice | 8～11 |
|  | Obvious stratification and a small amount of precipitation in juice | 4～7 |
|  | Obvious stratification and a large amount of precipitation | 0～3 |
| Color  (15 points) | The color is yellow and uniform | 12～15 |
|  | The color is light or dark yellow, and uniform | 8～11 |
|  | The color is yellow and slightly uneven | 4～7 |
|  | The color is light or dark yellow, and uneven | 0～3 |
| Aroma  (20 points) | It has a rich flavor of kiwifruit | 16～20 |
|  | It has some flavor of kiwifruit | 11～15 |
|  | It has almost no flavor of kiwifruit | 6～10 |
|  | It has an obvious peculiar odor | 0～5 |
| Sour and sweet  (20 points) | Juice is moderately sour and sweet | 16～20 |
|  | Juice is slightly sour or sweet | 11～15 |
|  | Juice is sour or sweet | 6～10 |
|  | Juice is too sour or sweet | 0～5 |
| Taste  (30 points) | It has a prominent kiwifruit flavor, and no bad taste | 23～30 |
|  | It has a slightly prominent kiwifruit flavor, and slightly refreshing | 16～22 |
|  | It has a general flavor of kiwifruit, and no refreshing | 8～15 |
|  | It has almost no kiwifruit flavor, and a seriously bad taste | 0～7 |

**Table S2 Component matrix and component coefficient matrix**

| Quality indices | Component matrix | | | Component coefficient matrix | | |
| --- | --- | --- | --- | --- | --- | --- |
|  | 1 | 2 | 3 | 1 | 2 | 3 |
| Overall sensory score | -0.266 | 0.808 | 0.473 | -0.103 | 0.65 | 0.6 |
| The increased colony count | 0.363 | -0.579 | 0.729 | 0.141 | -0.465 | 0.925 |
| AAC | 0.958 | 0.205 | -0.015 | 0.373 | 0.165 | -0.019 |
| TPC | 0.797 | 0.441 | 0.042 | 0.31 | 0.355 | 0.053 |
| TFC | 0.903 | -0.137 | -0.175 | 0.351 | -0.11 | -0.222 |

**Table S3 Comprehensive quality evaluation of different LAB fermented KJ**

| Strains | PC score (Z) | | | Comprehensive score (F) | Sort |
| --- | --- | --- | --- | --- | --- |
|  | 1 | 2 | 3 |  |  |
| LC | 0.396 | 0.452 | -1.595 | 0.071 | 4 |
| LP1 | -2.127 | -0.267 | 0.034 | -1.255 | 6 |
| LP2 | 0.357 | 1.713 | 0.667 | 0.776 | 1 |
| LB | 0.542 | -0.914 | 1.438 | 0.302 | 2 |
| LF | 0.769 | -0.858 | -0.162 | 0.171 | 3 |
| LH | 0.063 | -0.127 | -0.381 | -0.064 | 5 |

**Table S4 The color indices of different KJ samples**

|  | L^*^ | a^*^ | b^*^ | C^*^ | h° | ∆E |
| --- | --- | --- | --- | --- | --- | --- |
| CK | 65.04±0.06a | 1.29±0.06a | 37.70±0.13a | 37.72±0.13a | 88.04±0.09c | - |
| LBKJ | 60.57±0.07b | 1.10±0.06b | 37.00±0.14b | 37.02±0.14b | 88.30±0.09b | 4.52±0.03a |
| PBKJ | 60.37±0.10c | 1.03±0.09c | 36.67±0.19c | 36.69±0.19c | 88.40±0.14a | 4.75±0.14a |

Note: The different small letters indicate a significant difference (*p* < 0.05) among different KJ.

**Table S5 The VOCs of different KJ samples**

| Code | Compounds | Concentration (μg/L)^a^ | | | Formula | NIST_RI | CAS | cpd_ID |
| --- | --- | --- | --- | --- | --- | --- | --- | --- |
|  |  | CK | LBKJ | PBKJ |  |  |  |  |
| T1 | Sandaracopimaradiene | 331.21±143.94 | 257.55±171.57 | 385.93±7.21 | C_20_H_32_ | 1960 | 1686-56-2 | -- |
| T2 | m-Camphorene | 5.35±0.91 | 4.69±3.01 | 7.48±0.61 | C_20_H_32_ | 1960 | 20016-73-3 | -- |
| T3 | (2Z,6E)-Farnesol | 3.93±0.79b | 8.55±2.59a | 7.79±0.64a | C_15_H_26_O | 1697 | 3790-71-4 | C01126 |
| T4 | 4,8-Methanoazulen-9-ol, decahydro-2,2,4,8-tetramethyl-, stereoisomer | 6.19±0.93 | 7.38±1.45 | 8.01±1.56 | C_15_H_26_O | 1566 | 4586-22-5 | -- |
| T5 | Peruviol | 7.45±1.55 | 7.9±2.43 | 9.73±0.69 | C_15_H_26_O | 1544 | 142-50-7 | C09704 |
| T6 | β-Acorenol | 5.54±0.86 | 4.99±0.63 | 5.71±0.32 | C_15_H_26_O | 1649 | 28400-11-5 | -- |
| T7 | Farnesol | 9.26±0.24 | 8.05±4.51 | 11.57±1.9 | C_15_H_26_O | 1714 | 4602-84-0 | C01126 |
| T8 | Cubebol | 6.17±2.58 | 4.31±1.35 | 5.85±0.49 | C_15_H_26_O | 1515 | 23445-02-5 | C20172 |
| T9 | 8,14-Cedranoxide | 3.79±0.98 | 4.57±1.99 | 5.76±0.13 | C_15_H_24_O | 1546 | 18319-31-8 | -- |
| T10 | (2R,3R,6S)-6-Isopropyl-3-methyl-2-(prop-1-en-2-yl)-3-vinylcyclohexanone | 1261±152.2 | 1274.49±65.28 | 1150.11±84.61 | C_15_H_24_O | 1498 | 39020-72-9 | -- |
| T11 | Shyobunone | 1260.78±152.25 | 1271.26±69.81 | 1150.09±84.48 | C_15_H_24_O | 1503 | 21698-44-2 | -- |
| T12 | β-Sinensal |  | 8.75±2.28 | 9.47±1 | C_15_H_22_O | 1695 | 60066-88-8 | C09730 |
| T13 | Terpinyl propionate | 46.41±4.71 | 46.43±1.6 | 46.29±4.06 | C_13_H_22_O_2_ | 1432 | 80-27-3 | -- |
| T14 | (+)-epi-Bicyclosesquiphellandrene | 976.32±104.16 | 973.19±51.1 | 876.61±62.14 | C_15_H_24_ | 1482 | 54274-73-6 | -- |
| T15 | Bicyclo[4.4.0]dec-1-ene, 2-isopropyl-5-methyl-9-methylene- | 935.77±98.82 | 930.15±39.21 | 836.08±66.49 | C_15_H_24_ | 1507 | 150320-52-8 | -- |
| T16 | (Z,E)-α-Farnesene | 1229.05±127.49 | 1282.86±68.55 | 1164.6±80.51 | C_15_H_24_ | 1491 | 26560-14-5 | -- |
| T17 | β-Elemene | 9.08±1.46b | 12.59±1a | 11.26±0.48a | C_15_H_24_ | 1391 | 515-13-9 | C17094 |
| T18 | (1R,3aS,4aS,8aS)-1,4,4,6-Tetramethyl-1,2,3,3a,4,4a,7,8-octahydrocyclopenta[1,4]cyclobuta[1,2]benzene | 40.6±6.27b | 75.34±1.15a | 67.84±7.8a | C_15_H_24_ | 1403 | 94535-52-1 | -- |
| T19 | (-)-Isoledene | 4±0.64 | 5.57±0.92 | 5.68±1.18 | C_15_H_24_ | 1375 | 95910-36-4 | -- |
| T20 | Bicyclo[7.2.0]undecane, 10,10-dimethyl-2,6-bis(methylene)-, [1S-(1R*,9S*)]- | 809.89±88.84 | 831.66±48.82 | 755.24±55.84 | C_15_H_24_ | 1489 | 136296-38-3 | -- |
| T21 | δ-Guaiene | 3354.36±369 | 3396.54±180.69 | 3075.74±213.24 | C_15_H_24_ | 1505 | 3691-11-0 | C20174 |
| T22 | Naphthalene, decahydro-1,6-bis(methylene)-4-(1-methylethyl)-, (4.alpha.,4a.alpha.,8a.alpha.)- | 157.19±14.83a | 97.28±6.92b | 131.7±16.62a | C_15_H_24_ | 1459 | 30021-46-6 | -- |
| T23 | 1-Methyl-4-(6-methylhept-5-en-2-yl)cyclohexa-1,3-diene | 3439.61±418.53 | 3534.48±194.8 | 3195.82±222.02 | C_15_H_24_ | 1482 | 451-55-8 | -- |
| T24 | β-Guaiene | 982.36±112.57 | 981.9±46.73 | 884.32±68.38 | C_15_H_24_ | 1490 | 88-84-6 | -- |
| T25 | Isolongifolene | 9.62±1.1c | 16.26±0.59a | 13.72±1.55b | C_15_H_24_ | 1391 | 1135-66-6 | -- |
| T26 | β-Cadinene | 6.1±2.64 | 4.92±0.93 | 6.42±0.71 | C_15_H_24_ | 1518 | 523-47-7 | C09625 |
| T27 | Humulene | 411.47±50.27a | 281.66±22.43b | 381.14±42.88a | C_15_H_24_ | 1454 | 6753-98-6 | C09684 |
| T28 | Valerena-4,7(11)-diene | 21.75±1.86a | 16.01±1.74b | 18.3±3.38ab | C_15_H_24_ | 1460 | 351222-66-7 | C20480 |
| T29 | [1α,4aα,8aα]-1,2,4a,5,6,8a-hexahydro-4-7-dimethyl-1-[1-methylethyl]naphthalene | 3378.2±355.73 | 3419.14±178.14 | 3089.49±215.75 | C_15_H_24_ | 1496 | 31983-22-9 | -- |
| T30 | Eremophilene | 3348.58±369.58 | 3392.66±180.07 | 3072.89±212.52 | C_15_H_24_ | 1494 | 10219-75-7 | -- |
| T31 | (1S,5S,6R)-6-Methyl-2-methylene-6-(4-methylpent-3-en-1-yl)bicyclo[3.1.1]heptane | 1116.03±117.23 | 1125.63±59.68 | 1022.86±67.82 | C_15_H_24_ | 1492 | 15438-94-5 | -- |
| T32 | β-Farnesene | 1088.17±88.84a | 19.95±1.25b | 23.04±2.06ab | C_15_H_24_ | 1458 | 77129-48-7 | C09666 |
| T33 | α-Cubebene | 126.89±18.89 | 130.1±4.73 | 131.32±12.77 | C_15_H_24_ | 1351 | 17699-14-8 | C09647 |
| T34 | α-Muurolene | 3378.91±356.11 | 3420.42±178.34 | 3090.07±215.9 | C_15_H_24_ | 1499 | 10208-80-7 | C20272 |
| T35 | (1S,4S,4aR)-1-Isopropyl-4-methyl-7-methylene-1,2,3,4,4a,5,6,7-octahydronaphthalene | 57.18±6.75a | 39.74±5.1b | 51.77±6.9ab | C_15_H_24_ | 1463 | 157477-72-0 | -- |
| T36 | β-Himachalene | 3499.64±392.49 | 3551.34±198.84 | 3212.42±222.92 | C_15_H_24_ | 1500 | 1461-03-6 | -- |
| T37 | Cyclohexane, 1-ethenyl-1-methyl-2,4-bis(1-methylethenyl)-, (1R,2R,4S)-rel- | 8.79±1.66b | 12.73±0.66a | 11.29±1.06a | C_15_H_24_ | 1398 | 33880-83-0 | C17094 |
| T38 | (+)-Isoitalicene | 37.63±6.24b | 72.87±4.29a | 64.81±7.05a | C_15_H_24_ | 1395 | 94482-89-0 | -- |
| T39 | (Z)-α-Bisabolene | 4672.28±490.7 | 4724.89±251.94 | 4287.67±302.74 | C_15_H_24_ | 1504 | 29837-07-8 | -- |
| T40 | β-Farnesene | 28.45±3.96a | 19.84±1.4b | 22.88±2.28b | C_15_H_24_ | 1457 | 18794-84-8 | C09666 |
| T41 | 4a,8-Dimethyl-2-(prop-1-en-2-yl)-1,2,3,4,4a,5,6,7-octahydronaphthalene | 1.37±0.09 | 1.27±0.43 | 1.06±0.3 | C_15_H_24_ | 1492 | 103827-22-1 | -- |
| T42 | 3,6,6,9-Tetramethyl-4,4a,5,6,7,9a-hexahydro-1h-benzo[7]annulene | 2995.15±314.77 | 3039.52±173.47 | 2749.44±200.36 | C_15_H_24_ | 1495 | 24703-35-3 | -- |
| T43 | α-Bergamotene | 55.84±6.39 | 55.46±2.97 | 54.74±3.55 | C_15_H_24_ | 1435 | 17699-05-7 | C17088 |
| T44 | (+)-Sativene | 6.68±0.83b | 9.2±1.14a | 7.62±1.3ab | C_15_H_24_ | 1396 | 3650-28-0 | -- |
| T45 | Germacrene B | 10.3±1.68 | 8.65±1.17 | 8.21±0.2 | C_15_H_24_ | 1557 | 15423-57-1 | C09672 |
| T46 | (-)-β-Selinene | 1264.94±119.8 | 1267.55±69.95 | 1142.88±74.37 | C_15_H_24_ | 1486 | 17066-67-0 | C09723 |
| T47 | Zingiberene | 3501.73±397.85 | 3556.22±199.57 | 3216.43±226.39 | C_15_H_24_ | 1495 | 495-60-3 | C09750 |
| T48 | (-)-Germacrene D | 966.01±104.18 | 964.95±50.22 | 868.63±62.34 | C_15_H_24_ | 1481 | 23986-74-5 | C16142 |
| T49 | α-Curcumene | 3450.81±419.13 | 3544.98±196.19 | 3206.87±222.96 | C_15_H_22_ | 1483 | 644-30-4 | -- |
| T50 | Eudesma-2,4,11-triene | 666.59±73.75 | 678.43±36.58 | 607.38±42.66 | C_15_H_22_ | 1479 | 82462-31-5 | -- |
| T51 | Cuparene | 9.07±1.7 | 5.96±1.44 | 8.35±1.73 | C_15_H_22_ | 1505 | 16982-00-6 | -- |
| T52 | (5R,10R)-10-Methyl-6-methylene-2-(propan-2-ylidene)spiro[4.5]dec-7-ene | 1707.32±195.2 | 1747.15±87.72 | 1568.62±118.39 | C_15_H_22_ | 1495 | 28908-27-2 | -- |
| T53 | β-Calacorene | 68.36±9.93 | 76.8±0.7 | 67.12±9.93 | C_15_H_20_ | 1563 | 50277-34-4 | -- |
| T54 | trans-3a-cis-9a-1,2,3,3a,8,9,9a,9b-octahydro-4H-cyclopenta[def]phenanthrene | 0±0 | 113.52±2.44a | 86.19±9.44b | C_15_H_18_ | 1499 | 91110-19-9 | -- |
| T55 | Geranyl acetate | 231.33±28.32b | 499.06±9.94a | 440.37±53.03a | C_12_H_20_O_2_ | 1382 | 105-87-3 | C09861 |
| T56 | Linalyl acetate | 783.23±8.63 | 783.3±45.53 | 740.15±30.63 | C_12_H_20_O_2_ | 1257 | 115-95-7 | C09863 |
| T57 | Thymol acetate | 2.07±0.19 | 1.72±0.32 | 2.44±0.72 | C_12_H_16_O_2_ | 1355 | 528-79-0 | C09909 |
| T58 | Damascenone | 229.19±27.22b | 491.57±8.9a | 434.08±51.44a | C_13_H_18_O | 1386 | 23726-93-4 | -- |
| T59 | Citronellyl formate | 46.23±3.07 | 56.53±3.47 | 58.73±11.51 | C_11_H_20_O_2_ | 1275 | 105-85-1 | C_12_295 |
| T60 | Menthoglycol | 0±0 | 49.04±3.43a | 43.4±0.76b | C_10_H_20_O_2_ | 1301 | 3564-98-5 | C02904 |
| T61 | 2-Methylisoborneol | 937.51±13.29 | 938±52.32 | 930.27±44.62 | C_11_H_20_O | 1198 | 2371-42-8 | C20243 |
| T62 | Diosphenol | 1.14±0.35c | 9.65±0.8b | 14.99±1.24a | C_10_H_16_O_2_ | 1302 | 490-03-9 | C09854 |
| T63 | Geranic acid | 5.33±0.99 | 5.13±0.31 | 5.6±0.48 | C_10_H_16_O_2_ | 1355 | 459-80-3 | C16461 |
| T64 | Carvenone oxide | 561.06±13.26ab | 592.81±30.51a | 537.71±9.5b | C_10_H_16_O_2_ | 1261 | 5729-99-7 | -- |
| T65 | Ascaridole | 98.34±2.69 | 99.72±5.54 | 97.35±3.27 | C_10_H_16_O_2_ | 1244 | 512-85-6 | C09836 |
| T66 | Myrtenic acid | 8.9±1.08 | 9.21±0.37 | 9.2±1.56 | C_10_O_2_ | 1359 | 19250-17-0 | C11940 |
| T67 | Perillic acid | 3.83±1.42c | 221.85±9.4a | 175.98±15.91b | C_10_H_14_O_2_ | 1484 | 7694-45-3 | C11924 |
| T68 | p-Menthan-1-ol | 2837.28±185.66 | 2605.67±82.83 | 2795.97±116.86 | C_10_H_20_O | 1167 | 3901-93-7 | -- |
| T69 | l-Menthol | 2875.46±222.03 | 2609.03±72.49 | 2814.57±120.23 | C_10_H_20_O | 1171 | 2216-51-5 | C00400 |
| T70 | Neoisomenthol | 374.5±28.32a | 322.74±12.68b | 286.29±26.94b | C_10_H_20_O | 1187 | 491-02-1 | -- |
| T71 | cis-rose oxide | 801.84±119.56 | 684.52±19.03 | 723.01±16.72 | C_10_H_18_O | 1110 | 3033-23-6 | -- |
| T72 | cis-(+/-)-4-Thujanol | 45.66±11.11 | 32.61±2.68 | 36.81±4.28 | C_10_H_18_O | 1070 | 15537-55-0 | C02462 |
| T73 | (-)-Borneol | 4624.4±278.78 | 4423.69±191 | 4382.62±129.53 | C_10_H_18_O | 1170 | 464-45-9 | C01411 |
| T74 | (R)-Lavandulol | 2280.86±173.29 | 2283.81±119.55 | 2484.79±106.28 | C_10_H_18_O | 1170 | 498-16-8 | -- |
| T75 | 4-Thujanol | 122.52±17.67a | 78.51±2.59b | 86.15±7.2b | C_10_H_18_O | 1070 | 17699-16-0 | -- |
| T76 | (-)-α-Terpineol | 34.08±0.81b | 38.78±3.86ab | 40.97±4.17ab | C_10_H_18_O | 1190 | 10482-56-1 | C11393 |
| T77 | (-)-Terpinen-4-ol | 394.09±40.42a | 347.76±20.84ab | 334.89±8.41b | C_10_H_18_O | 1182 | 20126-76-5 | C17073 |
| T78 | p-Menthan-3-one | 2151.11±117.57 | 2063.9±90.82 | 2042.91±66.69 | C_10_H_18_O | 1169 | 10458-14-7 | -- |
| T79 | Geraniol | 611.53±10.83 | 624.27±32.32 | 586.06±29.18 | C_10_H_18_O | 1260 | 624-15-7 | C01500 |
| T80 | 3,3,6-Trimethylhepta-1,5-dien-4-ol | 508.76±109.61 | 482.92±36.57 | 583.93±22.01 | C_10_H_18_O | 1084 | 27644-04-8 | -- |
| T81 | Menthone | 2043.44±123.32 | 1913.34±83.23 | 1940.89±67.42 | C_10_H_18_O | 1154 | 89-80-5 | C00843 |
| T82 | Eucalyptol | 155.37±10.43a | 100.6±1.23c | 115.05±6.15b | C_10_H_18_O | 1030 | 470-82-6 | C09844 |
| T83 | 7-Methyl-3-methylene-6-octen-1-ol | 2521.78±106.73 | 2553.74±133.46 | 2518.13±59.1 | C_10_H_18_O | 1195 | 13066-51-8 | -- |
| T84 | Borneol | 4217±264.61 | 3863.85±103.4 | 4043.18±136.8 | C_10_H_18_O | 1167 | 507-70-0 | C01411 |
| T85 | p-Menthan-3-one | 1973.16±115.92 | 1836.53±81.53 | 1866.24±35.49 | C_10_H_18_O | 1164 | 491-07-6 | C11952 |
| T86 | Isogeraniol | 403.96±2.07 | 396.76±23.59 | 390.73±17.51 | C_10_H_18_O | 1240 | 5944-20-7 | -- |
| T87 | (-)-Ipsenol | 492.92±73.31 | 431.29±9.28 | 456.2±11.28 | C_10_H_18_O | 1097 | 35628-05-8 | -- |
| T88 | cis-β-Terpineol | 2858.2±228.92 | 2568.95±77.12 | 2771.06±119.13 | C_10_H_18_O | 1161 | 7299-40-3 | C17517 |
| T89 | Linalool | 1367.04±190.42 | 1196±27.15 | 1283.72±32.31 | C_10_H_18_O | 1099 | 78-70-6 | C03985 |
| T90 | γ-Terpineol | 40.14±2.3b | 46.19±3.45a | 43.55±2.28ab | C_10_H_18_O | 1197 | 586-81-2 | C17518 |
| T91 | Linalool | 607.42±27.53 | 624.32±28.05 | 583.84±24.97 | C_10_H_18_O | 1255 | 106-24-1 | C01500 |
| T92 | α-Terpineol | 32.49±0.56 | 38.01±4.56 | 39.39±4.59 | C_10_H_18_O | 1189 | 98-55-5 | -- |
| T93 | Fenchol | 694.87±101.2 | 602.37±12.58 | 632.64±15.75 | C_10_H_18_O | 1113 | 1632-73-1 | C02344 |
| T94 | cis-Chrysanthenol | 117.55±8.51 | 116.94±0.22 | 112.65±2.48 | C_10_H_16_O | 1162 | 55722-60-6 | -- |
| T95 | (±)-Nerol oxide | 24.93±1.69b | 24.01±0.62b | 28.42±1.62a | C_10_H_16_O | 1154 | 1786-08-9 | -- |
| T96 | Artemisia ketone | 99.45±50.55 | 24.49±1.88 | 28.81±4.82 | C_10_H_16_O | 1062 | 546-49-6 | -- |
| T97 | Isopulegone | 47.22±4.18 | 48.5±1.62 | 46.27±2.06 | C_10_H_16_O | 1179 | 529-00-0 | -- |
| T98 | cis-(+/-)- Pinocarveol | 1210.22±87.17 | 1224.41±66.09 | 1319.01±56.32 | C_10_H_16_O | 1178 | 6712-79-4 | -- |
| T99 | cis-Dihydrocarvone | 334.22±6.05 | 349.52±17.26 | 335.71±11.24 | C_10_H_16_O | 1195 | 3792-53-8 | -- |
| T100 | Neral | 444.8±10 | 443.16±11.12 | 426.41±18.97 | C_10_H_16_O | 1240 | 106-26-3 | C09847 |
| T101 | (±)-Piperitone | 1216.63±54.57 | 1229.78±61.52 | 1169.68±52.82 | C_10_H_16_O | 1253 | 89-81-6 | -- |
| T102 | Perillyl alcohol | 0±0 | 25.51±0.88a | 20.56±1.08b | C_10_H_16_O | 1296 | 536-59-4 | C02452 |
| T103 | Dehydro-1,8-cineole | 5.78±1.32ab | 4.28±0.53b | 6.34±0.16a | C_10_H_16_O | 992 | 92760-25-3 | -- |
| T104 | Geranial | 19.87±6.32 | 19.68±1.49 | 20.07±1.79 | C_10_H_16_O | 1270 | 141-27-5 | C01499 |
| T105 | (RS)-Pulegone | 94.07±1.24 | 105.63±21.1 | 92.31±5.41 | C_10_H_16_O | 1212 | 15932-80-6 | -- |
| T106 | Pinocamphone | 70.42±4.14ab | 63.23±11.72b | 81.41±4.2a | C_10_H_16_O | 1160 | 547-60-4 | -- |
| T107 | Thujone | 456.44±66.11 | 384.25±27.43 | 431.99±30.43 | C_10_H_16_O | 1103 | 546-80-5 | C09906 |
| T108 | p-Mentha-1,5-dien-7-ol | 32.06±1.79 | 31.68±0.46 | 30.08±2.13 | C_10_H_16_O | 1194 | 19876-45-0 | -- |
| T109 | Carvenone | 6084.73±65.12 | 6089.09±287.06 | 5734.75±221.68 | C_10_H_16_O | 1257 | 499-74-1 | -- |
| T110 | Phellandral | 10.04±2.22 | 12.57±1.71 | 13.42±3.32 | C_10_H_16_O | 1276 | 21391-98-0 | -- |
| T111 | α-Pinene oxide | 914.62±134.19 | 789.71±20.3 | 837.56±21.04 | C_10_H_16_O | 1095 | 1686-14-2 | C02759 |
| T112 | Isopinocamphone | 1605.34±125.5b | 1675.76±117.83ab | 1854.85±79.66a | C_10_H_16_O | 1173 | 15358-88-0 | -- |
| T113 | Citral | 44.4±5.16b | 63.28±4.47a | 66.7±12.77a | C_10_H_16_O | 1273 | 5392-40-5 | C01499 |
| T114 | (±)-Fenchone | 690.81±100.83 | 601.86±14.16 | 632.04±16.21 | C_10_H_16_O | 1096 | 1195-79-5 | C09859 |
| T115 | Eucarvone | 2722.92±32.32 | 2713.31±151.03 | 2585.18±105.37 | C_10_H_14_O | 1243 | 503-93-5 | C16955 |
| T116 | Pinocarvone | 18.07±0.45b | 18.97±0.54a | 16.24±0.27c | C_10_H_14_O | 1164 | 30460-92-5 | C09884 |
| T117 | (+)-Verbenone | 2.59±0.39 | 2.67±0.4 | 2.79±0.26 | C_10_H_14_O | 1228 | 18309-32-5 | C09913 |
| T118 | D-Carvone | 1234.29±22.29 | 1221.94±85.81 | 1156.02±52.13 | C_10_H_14_O | 1246 | 2244-16-8 | C11383 |
| T119 | Perillene | 2317.23±335.16 | 2065.4±64.12 | 2221.88±52.81 | C_10_H_14_O | 1101 | 539-52-6 | -- |
| T120 | (±)-Myrtenal | 61±1.85b | 68.32±3.4a | 68.49±3.06a | C_10_H_14_O | 1193 | 564-94-3 | C11939 |
| T121 | Carvone | 1175.8±25.41 | 1191.57±67.99 | 1133.95±59.11 | C_10_H_14_O | 1242 | 99-49-0 | -- |
| T122 | (+)-Isopiperitenone | 13.35±2.38 | 14.19±3.04 | 12.01±0.35 | C_10_H_14_O | 1190 | 16750-82-6 | C02289 |
| T123 | Umbellulone | 24.12±0.53 | 24.3±0.87 | 23.28±1.32 | C_10_H_14_O | 1171 | 24545-81-1 | -- |
| T124 | (-)-Carvone | 1239.35±20.88 | 1229.11±66.71 | 1167.09±56.1 | C_10_H_14_O | 1254 | 6485-40-1 | C01767 |
| T125 | (±)-cis-Pinane | 234.52±73.96a | 115.4±9.21b | 124.82±4.26b | C_10_H_18_ | 972 | 33626-25-4 | -- |
| T126 | 2,6-Dimethyl-2-trans-6-octadiene | 79.51±13.19 | 84.44±1.19 | 94.67±2.13 | C_10_H_18_ | 993 | 2609-23-6 | -- |
| T127 | (Z)-β-Ocimene | 141.68±13.6a | 103.22±1.08b | 118.31±6.25b | C_10_H_16_ | 1038 | 3338-55-4 | -- |
| T128 | β-Ocimene | 141.04±13.88a | 101.78±0.52b | 116.31±6.55b | C_10_H_16_ | 1037 | 13877-91-3 | -- |
| T129 | (1R)-α-Pinene | 6.09±0.17a | 4.11±0.31b | 4.35±0.77b | C_10_H_16_ | 932 | 7785-70-8 | C06306 |
| T130 | β-Phellandrene | 46.36±3.05 | 41.96±2.77 | 41.69±4.45 | C_10_H_16_ | 1031 | 555-10-2 | C19818 |
| T131 | (E)-β-Ocimene | 13.63±2.19b | 18.75±1.01a | 18.17±0.2a | C_10_H_16_ | 1049 | 3779-61-1 | C09873 |
| T132 | alpha-Phellandrene | 11.01±0.88 | 9.68±0.73 | 10.03±1.65 | C_10_H_16_ | 1006 | 99-83-2 | C11391 |
| T133 | (+)-3-Carene | 10.19±0.17 | 9.7±0.8 | 9.3±0.35 | C_10_H_16_ | 1010 | 498-15-7 | C11382 |
| T134 | α-Pinene | 5.74±0.38a | 3.95±0.17b | 4.42±1.08ab | C_10_H_16_ | 937 | 80-56-8 | C09880 |
| T135 | 4-Carene | 10.13±0.09 | 9.37±0.77 | 9.46±0.61 | C_10_H_16_ | 1009 | 29050-33-7 | -- |
| T136 | Limonene | 43.37±4.23a | 37.39±0.97b | 40.72±1.98ab | C_10_H_16_ | 1026 | 138-86-3 | C06078 |
| T137 | γ-Terpinene | 121.91±17.12a | 77.39±2.06b | 85.12±7.57b | C_10_H_16_ | 1060 | 99-85-4 | C09900 |
| T138 | α-Terpinene | 7.62±1.49 | 7.15±0.34 | 6.91±0.32 | C_10_H_16_ | 1012 | 99-86-5 | C09898 |
| T139 | (S)-Limonene | 43.66±4.28a | 37.53±0.64b | 41.13±2.56ab | C_10_H_16_ | 1029 | 5989-54-8 | C00521 |
| T140 | 1,5,8-p-Menthatriene | 1.82±0.12 | 1.47±0.3 | 1.7±0.52 | C_10_H_14_ | 1111 | 21195-59-5 | -- |
| T141 | 1,3,8-p-Menthatriene | 13.9±3.49 | 15.46±1.21 | 16.88±1.02 | C_10_H_14_ | 1119 | 18368-95-1 | -- |
| T142 | 1-Isopropyl-4-methylenebicyclo[3.1.0]hex-2-ene | 2.14±0.35 | 1.67±0.23 | 1.81±0.51 | C_10_H_14_ | 956 | 36262-09-6 | -- |
| ***Terpenoids*** |  | 110963.78±2383.23 | 109433.59±5056.97 | 105697.99±5430.42 |  |  |  |  |
| Hc1 | Meperidine | 16.39±2.62 | 11.74±7.15 | 19.47±0.13 | C_15_H_21_NO_2_ | 1758 | 57-42-1 | C07128 |
| Hc2 | (4-Dimethylamino-benzyl)-thiazol-2-yl-amine | 57.71±24.43 | 45.78±28.43 | 64.68±1.58 | C_12_H_15_N_3_S | 1966 | 13159-95-0 | -- |
| Hc3 | δ-Tetradecalactone | 4.26±1.25ab | 2.68±1.65b | 5.29±0.39a | C_14_H_26_O_2_ | 1929 | 2721-22-4 | -- |
| Hc4 | 2,2'-Isopropylidenebis(5-methylfuran) | 4.34±0.29ab | 3.35±0.64b | 4.48±0.6a | C_13_H_16_O_2_ | 1458 | 59212-75-8 | -- |
| Hc5 | 1-Pentanol, 5-[(tetrahydro-2H-pyran-2-yl)oxy]- | 108.46±16.97 | 112.39±5.26 | 109.96±9.04 | C_10_H_20_O_3_ | 1486 | 76102-74-4 | -- |
| Hc6 | Thiocyclam | 128.19±23.05 | 121.61±8.17 | 127.06±10.04 | C_5_H_11_NS_3_ | 1491 | 31895-21-3 | C11473 |
| Hc7 | Linalool oxide | 2048.08±158.33 | 1931.25±73.4 | 2082.55±92.81 | C_10_H_18_O_2_ | 1173 | 14049-11-7 | -- |
| Hc8 | Linalool oxide III | 2037.75±158.65 | 1919.6±71.94 | 2076.3±92.31 | C_10_H_18_O_2_ | 1171 | 14009-71-3 | -- |
| Hc9 | (±)-Massoia lactone | 8.27±1.42c | 90.39±1.61a | 74.37±6.78b | C_10_H_16_O_2_ | 1501 | 54814-64-1 | -- |
| Hc10 | Ethyl 2-furanpropanoate | 107.38±2.6 | 107.74±9.8 | 118.8±17.3 | C_9_H_12_O_3_ | 1192 | 10031-90-0 | -- |
| Hc11 | 2-Pentanoylthiophene | 2.83±0.6 | 1.92±0.56 | 2.45±0.49 | C_9_H_12_OS | 1328 | 53119-25-8 | -- |
| Hc12 | 2-Heptylfuran | 104.26±2.02 | 112.18±6.33 | 109.92±5.98 | C_11_H_18_O | 1195 | 3777-71-7 | -- |
| Hc13 | 2-sec-Butyl-3-methoxypyrazine | 4.72±0.19 | 4.16±0.14 | 4.28±0.56 | C_9_H_14_N_2_O | 1175 | 24168-70-5 | -- |
| Hc14 | Elsholtziaketone | 26.24±0.09b | 32.24±2.23a | 30.51±2.33a | C_10_H_14_O_2_ | 1201 | 488-05-1 | -- |
| Hc15 | 2-Hexanoylfuran | 5718.04±152.53 | 5934.62±281.61 | 5618.51±199.43 | C_10_H_14_O_2_ | 1239 | 14360-50-0 | C02088 |
| Hc16 | 5-SEC-BUTYL-2,3-DIMETHYLPYRAZINE, 98% | 2.57±0.81 | 3±0.32 | 3.02±0.26 | C_10_H_16_ | 1229 | 32263-00-6 | -- |
| Hc17 | 1-Butanamine, N-(2-pyridinylmethylene)- | 40.29±6.8b | 76.82±2.18a | 67.99±5.79a | C_10_H_14_N_2_ | 1401 | 7032-24-8 | -- |
| Hc18 | 1,3-Dithiolane, 2-acetyl-2-methyl- | 53.68±1.36 | 54.75±2.67 | 51.72±2.68 | C_6_H_10_OS_2_ | 1252 | 33266-07-8 | -- |
| Hc19 | 1H-Pyrrolo(2,3-b)pyridine, 2-isopropyl- | 9.92±1.48b | 19.95±0.84a | 17.75±2.15a | C_10_H_12_N_2_ | 1392 | 27257-18-7 | -- |
| Hc20 | 2-Acetylbenzofuran | 10±1.05b | 20.11±0.67a | 17.88±2.09a | C_10_H_8_O_2_ | 1395 | 1646-26-0 | -- |
| Hc21 | 3-Phenylthiophene | 9.5±0.93 | 11.02±1.04 | 9.65±0.79 | C_10_H_8_S | 1368 | 2404-87-7 | -- |
| Hc22 | 2-Methyl-5-oxotetrahydro-2-furanyl acetate | 6.86±1.13b | 7.49±0.52b | 9.25±0.81a | C_7_H_10_O_4_ | 1226 | 57681-51-3 | -- |
| Hc23 | Whiskey lactone | 0±0 | 16.77±0.95a | 14.8±1.01b | C_9_H_16_O_2_ | 1302 | 39212-23-2 | -- |
| Hc24 | 2-Pentylthiophene | 276.12±308.39 | 235.65±40.72 | 265.83±9.83 | C_9_H_14_S | 1170 | 4861-58-9 | -- |
| Hc25 | 2,3,3a,4,5,7a-Hexahydro-3,6-dimethylbenzofuran | 5.76±0.51 | 5.82±0.46 | 6.26±0.3 | C_10_H_16_O | 1192 | 70786-44-6 | -- |
| Hc26 | 2-Hexylfuran | 689.48±100.95 | 600.94±13.83 | 630.61±15.85 | C_10_H_16_O | 1095 | 3777-70-6 | -- |
| Hc27 | 2-Isopropyl-3-methoxypyrazine | 30.2±4.72 | 26.99±0.34 | 27.76±0.51 | C_8_H_12_N_2_O | 1097 | 25773-40-4 | -- |
| Hc28 | 1,4-Benzodioxan-6-amine | 58.41±7.7a | 41.88±2.29b | 44.61±5.13b | C_8_H_9_NO_2_ | 1456 | 22013-33-8 | -- |
| Hc29 | 1H-Pyrrolo[3,2-d]pyrimidine-2,4(3H,5H)-dione | 0±0 | 2.67±0.23a | 1.5±0.46b | C_6_H_5_N_3_O_2_ | 1475 | 65996-50-1 | -- |
| Hc30 | 2-Acetyl-3,5-dimethylpyrazine | 34.29±1.62 | 30.38±2.03 | 30.44±3.33 | C_8_H_10_N_2_O | 1174 | 54300-08-2 | -- |
| Hc31 | 2-Pentylpyridine | 46±3.17 | 46.19±3.45 | 46.88±1.18 | C_10_H_15_N | 1202 | 2294-76-0 | -- |
| Hc32 | 4,7-Dimethylbenzofuran | 6.84±0.61 | 7.14±0.48 | 7.39±0.3 | C_10_H_10_O | 1220 | 28715-26-6 | -- |
| Hc33 | 5-Ethyl-3-hydroxy-4-methyl-2(5H)-furanone | 2678.31±65.06 | 2753.7±112.97 | 2664.28±123.2 | C_7_H_10_O_3_ | 1195 | 698-10-2 | -- |
| Hc34 | Kojic acid | 132.19±16.07a | 87.18±10.71b | 121.85±15.02a | C_6_H_6_O_4_ | 1462 | 501-30-4 | C14516 |
| Hc35 | Hygrine | 23.87±4.8c | 30.79±0.46b | 37.84±0.85a | C_8_H_15_NO | 1092 | 496-49-1 | C06179 |
| Hc36 | Guvacoline | 1212.33±12.68 | 1194.7±73.15 | 1139.3±63.07 | C_7_H_11_NO_2_ | 1250 | 495-19-2 | C16821 |
| Hc37 | 3-Butylthiophene | 1013.14±158.43 | 887.89±17.28 | 948.88±22.03 | C_8_H_12_S | 1093 | 34722-01-5 | -- |
| Hc38 | 2-Methyl-3-(methylthio)pyrazine | 1.82±0.19 | 1.84±0.11 | 1.92±0.14 | C_6_H_8_N_2_S | 1184 | 2882-20-4 | -- |
| Hc39 | Tropinone | 43.38±2.15 | 47.63±2.9 | 46.67±0.5 | C_8_H_13_NO | 1198 | 532-24-1 | C00783 |
| Hc40 | 3-Ethyl-4-methyl-1H-pyrrole-2,5-dione | 636.32±10.19 | 647.54±31.49 | 610.75±27.31 | C_7_H_9_NO_2_ | 1239 | 20189-42-8 | -- |
| Hc41 | 2-Pentylfuran | 31.09±3.4b | 28.17±0.67b | 40.98±3.32a | C_9_H_14_O | 993 | 3777-69-3 | -- |
| Hc42 | 3,5-Dimethylpyrazole-1-carboxamidine | 6418.96±921.42 | 5530.28±137.13 | 5843.62±143.63 | C_6_H_10_N4 | 1096 | 22906-75-8 | -- |
| Hc43 | 2-Ethoxy-3-methylpyrazine | 224.91±28.4 | 195.36±12.62 | 216.93±3.32 | C_7_H_10_N_2_O | 1065 | 32737-14-7 | -- |
| Hc44 | 3-Acetyl-2,5-dimethylfuran | 295.2±42.51 | 251.1±6.88 | 269.02±7.48 | C_8_H_10_O_2_ | 1099 | 10599-70-9 | -- |
| Hc45 | 2-Methyl-6-nitropyridine | 50.99±4.58 | 45.71±3.02 | 46.09±2.21 | C_6_H_6_N_2_O_2_ | 1183 | 18368-61-1 | -- |
| Hc46 | Tetramethylpyrazine | 52.19±9.87 | 46.07±3.31 | 55.53±2.39 | C_8_H_12_N_2_ | 1088 | 1124-11-4 | -- |
| Hc47 | 4-Isobutylpyrimidine | 3.03±0.43 | 2.96±0.68 | 3.11±0.5 | C_8_H_12_N_2_ | 1015 | 98489-37-3 | -- |
| Hc48 | 2-Methyl-5-isopropylpyrazine | 34.97±7.69 | 26.12±1.06 | 28.38±2.46 | C_8_H_12_N_2_ | 1059 | 13925-05-8 | -- |
| Hc49 | 5-Methyl-6,7-dihydro-5H-cyclopentapyrazine | 6.79±0.56b | 9.71±0.11a | 9.83±0.4a | C_8_H_10_N_2_ | 1144 | 23747-48-0 | -- |
| Hc50 | 7-Methyl-5H-pyrrolo[2,3-b]pyrazine | 5.4±0.65 | 5.5±0.37 | 4.91±0.56 | C_7_H_7_N_3_ | 1252 | 20321-99-7 | -- |
| Hc51 | Oxindole | 321.56±33.18 | 327.26±19.28 | 297.29±23.8 | C_8_H_7_NO | 1487 | 59-48-3 | C12312 |
| Hc52 | 2-Thiophenemethanethiol | 1017.94±153.52 | 894.92±19.38 | 953.44±23.06 | C_5_H_6_S_2_ | 1105 | 6258-63-5 | -- |
| Hc53 | 3-Acetyl-2-oxazolidinone | 15.87±0.8 | 14.1±1.48 | 15.69±1.26 | C_5_H_7_NO_3_ | 1174 | 1432-43-5 | -- |
| Hc54 | 2-Acetyl-2-thiazoline | 16.6±4.64b | 21.84±2.03ab | 25.44±3.2a | C_5_H_7_NOS | 1106 | 29926-41-8 | -- |
| Hc55 | Furaneol | 3560.16±419.62 | 3176.54±54.33 | 3436.8±79.65 | C_6_H_8_O_3_ | 1070 | 3658-77-3 | C20717 |
| Hc56 | 5-Methyl-2-furanmethanethiol | 19.12±5.49 | 21.55±0.87 | 18.04±0.63 | C_6_H_8_OS | 995 | 59303-05-8 | -- |
| Hc57 | 6-ethyl-5,6-dihydro-2H-pyran-2-one | 506.63±30.08 | 419.17±104.05 | 438.03±86.66 | C_7_H_10_O_2_ | 1160 | 19895-35-3 | -- |
| Hc58 | Isomaltol | 18.17±4.02ab | 14.33±0.47b | 20.54±0.83a | C_6_H_6_O_3_ | 989 | 3420-59-5 | -- |
| Hc59 | 2-Acetylthiophene | 5.25±0.89 | 4.43±0.34 | 5.57±0.98 | C_6_H_6_OS | 1092 | 88-15-3 | -- |
| Hc60 | 2,3,4,5-Tetrahydro-6-propylpyridine | 7.77±1.03 | 7.96±0.52 | 7.7±0.47 | C_8_H_15_N | 1028 | 1604-01-9 | C10138 |
| Hc61 | 4-Methyl-5-vinylthiazole | 29.5±1.88a | 18.63±0.43c | 21.21±1.11b | C_6_H_7_NS | 1027 | 1759-28-0 | -- |
| Hc62 | 2-Methoxy-6-methylpyrazine | 7.73±1.13 | 7.65±0.72 | 8.36±0.21 | C_6_H_8_N_2_O | 994 | 2882-21-5 | -- |
| Hc63 | 2-Acetyl-5-methylfuran | 98.63±26.71 | 85.65±9.01 | 97.61±20.99 | C_7_H_8_O_2_ | 1039 | 1193-79-9 | -- |
| Hc64 | Pyrazinamide | 448.75±5.12 | 442.78±28.9 | 417.36±19.36 | C_5_H_5_N_3_O | 1250 | 98-96-4 | C01956 |
| Hc65 | β-2-Furanacrolein | 135.56±18.13 | 113.18±3.75 | 119.38±2.9 | C_7_H_6_O_2_ | 1111 | 623-30-3 | -- |
| Hc66 | 3-Acetylpyridine | 166.38±23.13 | 143.8±2.56 | 148.28±2.84 | C_7_H_7_NO | 1112 | 350-03-8 | -- |
| Hc67 | 2-Methyl-1,3-dithiolane | 15.45±1.94a | 12.78±0.37b | 14.03±0.85ab | C_4_H_8_S_2_ | 1026 | 5616-51-3 | -- |
| Hc68 | Benzofuran | 10.24±2.97 | 7.35±0.95 | 9.15±0.8 | C_8_H_6_O | 1000 | 271-89-6 | C14512 |
| Hc69 | Methylsuccinic anhydride | 28.14±14a | 9.89±0.66b | 9.08±1.5b | C_5_H_6_O_3_ | 1057 | 4100-80-5 | -- |
| Hc70 | 2-Methyl-3-furanthiol | 1749.65±790 | 2305.55±87.13 | 2169.38±332.92 | C_5_H_6_OS | 870 | 28588-74-1 | -- |
| Hc71 | 2-Ethylpiperidine | 118.16±9.54a | 90.58±4.55b | 99.58±9.13b | C_7_H_15_N | 1026 | 1484-80-6 | -- |
| Hc72 | 2-Acetylpyrrolidine | 9.58±0.55 | 7.3±0.11c | 8.22±0.52b | C_6_H_11_NO | 1042 | 60026-20-2 | -- |
| Hc73 | 1H-1,2,4-Triazole-1-ethanol | 679.11±99.94 | 607.36±15.87 | 653.81±14.44 | C_4_H_7_N_3_O | 1095 | 3273-14-1 | -- |
| Hc74 | 5-Ethyl-2(5H)-furanone | 655.16±149.28 | 357.46±28.71b | 390.24±11.07b | C_6_H_8_O_2_ | 966 | 2407-43-4 | -- |
| Hc75 | 3-Ethylthiophene | 400.12±178.1 | 526.08±20.44 | 500.55±79.89 | C_6_H_8_S | 869 | 1795-01-3 | -- |
| Hc76 | N-Vinyl-2-pyrrolidone | 670.72±98.18 | 593.37±14.64 | 641.49±15.63 | C_6_H_9_NO | 1102 | 88-12-0 | C19548 |
| Hc77 | 2-Propylimidazole | 1998.38±289.94 | 1759.18±46.68 | 1876.9±47.59 | C_6_H_10_N_2_ | 1095 | 50995-95-4 | -- |
| Hc78 | 4-Acetylpyrazole | 4547.03±267.65 | 4340.31±187.6 | 4325.36±134.02 | C_5_H_6_N_2_O | 1156 | 25016-16-4 | -- |
| Hc79 | 1-(1H-Imidazol-1-yl)ethanone | 108.57±48.45a | 45.24±5.04b | 47.21±4.59b | C_5_H_6_N_2_O | 950 | 2466-76-4 | C02560 |
| Hc80 | 4-Pyridinecarboxaldehyde | 17.59±1.54a | 9.04±1.34b | 10.82±0.86b | C_6_H_5_NO | 976 | 872-85-5 | -- |
| Hc81 | δ-Valerolactone | 188.26±29.46a | 104.85±5.91b | 116.9±2.32b | C_5_H_8_O_2_ | 961 | 542-28-9 | C02240 |
| Hc82 | 1H-Tetrazole-1,5-diamine | 25.96±4.1c | 156.15±7.84a | 128.8±10.94b | CH_4_N_6_ | 1511 | 2165-21-1 | -- |
| Hc83 | 2-Iminopiperidine | 983.08±98.27a | 699.56±70.7b | 803.7±20.34b | C_5_H_10_N_2_ | 824 | 22780-54-7 | -- |
| Hc84 | 4,5-Dihydro-5,5-dimethyl-1H-pyrazole | 28.06±13.14a | 9.62±1.5b | 14.13±2.42ab | C_5_H_10_N_2_ | 983 | 4320-85-8 | -- |
| Hc85 | Maleimide | 27.89±1.93a | 24.48±0.5b | 26.18±1.07ab | C_4_H_3_NO_2_ | 1033 | 541-59-3 | C07272 |
| Hc86 | 3-Furancarboxaldehyde | 64.47±14.61a | 8.92±1.01b | 12.53±1.01b | C_5_H_4_O_2_ | 831 | 498-60-2 | -- |
| Hc87 | 4H-Pyran-4-one | 6.08±1.11a | 2.89±0.36b | 3.52±0.37b | C_5_H_4_O_2_ | 972 | 108-97-4 | -- |
| Hc88 | Pyrrole-2-carboxaldehyde | 6.5±1.77 | 4.41±0.4 | 4.39±0.02 | C_5_H_5_NO | 1012 | 1003-29-8 | -- |
| Hc89 | 2-Pyridone | 2434.12±355.6 | 2135.26±58.37 | 2256.85±57.15 | C_5_H_5_NO | 1094 | 142-08-5 | C02502 |
| Hc90 | 4-Aminopyridine | 893.75±43.74a | 815.15±42.41b | 794.7±17.12b | C_5_H_6_N_2_ | 1158 | 504-24-5 | C13728 |
| Hc91 | 3-Cyanopyrrole | 11.98±2.01a | 7.76±0.11b | 8.13±0.69b | C_5_H_4_N_2_ | 1044 | 7126-38-7 | -- |
| Hc92 | 1,5-Diazabicyclo[3.1.0]hexane | 631.38±163.98a | 322.85±21.94b | 362.47±5.43b | C_4_H_8_N_2_ | 951 | 13090-31-8 | -- |
| ***Heterocyclic compounds*** |  | 47206.81±3161.15 | 44108.7±1695.25 | 45110.72±1681.65 |  |  |  |  |
| E1 | Phosphoric acid, dibutyl isopropenyl ester | 2.1±0.89b | 8.46±0.89a | 7.59±0.77a | C_11_H_23_O_4_P | 1474 | 5954-40-5 | -- |
| E2 | Octyl trifluoroacetate | 74.51±28.59a | 29.78±2.39b | 23.44±4.22b | C_10_H_17_F_3_O_2_ | 1059 | 2561-21-9 | -- |
| E3 | Bornyl butyrate | 0±0 | 84.6±2.68a | 67.51±6.2b | C_14_H_24_O_2_ | 1476 | 13109-70-1 | -- |
| E4 | Neryl isobutyrate | 6.21±0.76c | 60.93±0.88a | 47.61±2.96b | C_14_H_24_O_2_ | 1475 | 2345-24-6 | -- |
| E5 | Dimethylbenzylcarbinyl butyrate | 128.11±22.88 | 129.62±8.25 | 134.94±11.08 | C_14_H_20_O_2_ | 1493 | 10094-34-5 | -- |
| E6 | Methyl laurate | 4.36±0.68a | 1.51±0.14b | 2.22±0.77b | C_13_H_26_O_2_ | 1526 | 111-82-0 | -- |
| E7 | Pentyl octanoate | 161.49±19.85 | 166.22±8.39 | 151.96±10.98 | C_13_H_26_O_2_ | 1484 | 638-25-5 | C04780 |
| E8 | 2-Furanylmethyl heptanoate | 15.76±2.84c | 228.48±2.35a | 175.55±15.16b | C_12_H_18_O_3_ | 1480 | 39481-28-2 | -- |
| E9 | Phenoxyethyl isobutyrate | 1043.09±109.94 | 1050.44±57.88 | 941.18±52.69 | C_12_H_16_O_3_ | 1488 | 103-60-6 | -- |
| E10 | Methyl p-tert-butylphenylacetate | 19.11±1.81 | 19.53±1.62 | 17.94±2.18 | C_13_H_18_O_2_ | 1491 | 3549-23-3 | -- |
| E11 | Octyl butyrate | 2.83±0.3c | 5.18±0.36a | 4.53±0.18b | C_12_H_24_O_2_ | 1393 | 110-39-4 | -- |
| E12 | δ-Dodecalactone | 14.82±0.86 | 12.91±6.95 | 17.41±1.03 | C_12_H_22_O_2_ | 1720 | 713-95-1 | -- |
| E13 | γ-Terpinyl acetate | 0±0 | 3.2±0.5 | 2.5±0.71 | C_12_H_20_O_2_ | 1341 | 10235-63-9 | C12302 |
| E14 | Ethyl (E,Z)-2,4-decadienoate | 0±0 | 68.93±0.89a | 51.9±4.82b | C_12_H_20_O_2_ | 1479 | 3025-30-7 | C08486 |
| E15 | Acetic acid--(4S)-3,3,6-trimethylhepta-1,5-dien-4-ol (1/1) | 2328.4±180.66 | 2091.41±65.93 | 2264.43±84.54 | C_12_H_20_O_2_ | 1173 | 3465-88-1 | -- |
| E16 | Neryl acetate | 5.23±0.65 | 5.64±0.3 | 5.42±0.37 | C_12_H_20_O_2_ | 1362 | 141-12-8 | -- |
| E17 | (±)-α-Terpinyl acetate | 1.42±0.36 | 1.17±0.2 | 1.32±0.26 | C_12_H_20_O_2_ | 1350 | 80-26-2 | C12300 |
| E18 | Isopulegol acetate | 135.7±5.74 | 139.9±7.05 | 131.93±5.1 | C_12_H_20_O_2_ | 1259 | 89-49-6 | -- |
| E19 | Ethyl safranate | 47.5±4.39 | 47.62±1.48 | 47.34±4.09 | C_12_H_18_O_2_ | 1434 | 35044-59-8 | -- |
| E20 | (+)-cis-Verbenol, acetate | 6.12±1.21 | 7.75±0.46 | 8.21±1.24 | C_12_H_18_O_2_ | 1279 | 29135-27-1 | -- |
| E21 | 2-Phenylethyl isobutyrate | 4.73±0.29c | 10.34±0.44a | 8.9±0.95b | C_12_H_16_O_2_ | 1396 | 103-48-0 | -- |
| E22 | Isobutyl phenylacetate | 56.71±8.32c | 111.96±3.05a | 97.02±8.82b | C_12_H_16_O_2_ | 1393 | 102-13-6 | -- |
| E23 | Benzyl 2-methylbutyrate | 58.13±8.26b | 114.31±4.11a | 101.53±11.18a | C_12_H_16_O_2_ | 1392 | 56423-40-6 | -- |
| E24 | Benzyl isovalerate | 58.94±9.16b | 113.65±3.75a | 102.13±11.57a | C_12_H_16_O_2_ | 1395 | 103-38-8 | -- |
| E25 | Benzyl pentanoate | 59.7±8.76b | 112.86±1.92a | 105.57±10.72a | C_12_H_16_O_2_ | 1396 | 10361-39-4 | -- |
| E26 | Benzyl tiglate | 4837.61±529.33 | 4901.32±237.49 | 4406±318.84 | C_12_H_14_O_2_ | 1498 | 37526-88-8 | -- |
| E27 | 2-Nonyl acetate | 244.91±4.01 | 252.3±12.47 | 239.43±10.73 | C_11_H_22_O_2_ | 1236 | 14936-66-4 | -- |
| E28 | Nonyl acetate | 0±0 | 6.19±0.4b | 10.12±1.06a | C_11_H_22_O_2_ | 1309 | 143-13-5 | -- |
| E29 | Isoamyl hexanoate | 428.79±12.7 | 437.48±26.9 | 408.38±16.49 | C_11_H_22_O_2_ | 1250 | 2198-61-0 | -- |
| E30 | Hexyl isovalerate | 788.57±29.24 | 811.75±65.81 | 754.82±42.61 | C_11_H_22_O_2_ | 1244 | 10032-13-0 | -- |
| E31 | Heptyl isobutyrate | 412.09±7.91 | 421.53±16.03 | 398.65±17.08 | C_11_H_22_O_2_ | 1247 | 2349-13-5 | -- |
| E32 | 2-Methylbutyl hexanoate | 224.92±9.43 | 227.09±12.66 | 223.14±16.08 | C_11_H_22_O_2_ | 1247 | 2601-13-0 | -- |
| E33 | cis-3-Hexenyl isovalerate | 1195.04±13.23 | 1208.35±53.55 | 1144.2±45.26 | C_11_H_20_O_2_ | 1238 | 35154-45-1 | -- |
| E34 | (Z)-6-Nonen-1-yl acetate | 0±0 | 44.82±2.44a | 37.49±1.38b | C_11_H_20_O_2_ | 1308 | 76238-22-7 | -- |
| E35 | cis-3-Hexenyl valerate | 1150.5±12.08 | 1177.52±47.71 | 1113.77±45.14 | C_11_H_20_O_2_ | 1237 | 35852-46-1 | -- |
| E36 | Geranyl formate | 0±0 | 40.07±3.04a | 33.49±1.04b | C_11_H_18_O_2_ | 1301 | 105-86-2 | C12294 |
| E37 | Phenyl propyl carbonate | 7.7±0.13 | 0±0 | 0±0 | C_10_H_12_O_3_ | 1335 | 13183-16-9 | -- |
| E38 | 3-Sulfanylhexyl acetate | 11.98±1.06 | 12.27±0.91 | 11.51±0.25 | C_8_H_16_O_2_S | 1248 | 136954-20-6 | -- |
| E39 | Propanedioic acid, 2-amino-, 1,3-diethyl ester | 4.16±0.82 | 3.88±0.57 | 4.09±0.74 | C_7_H_13_NO_4_ | 1231 | 6829-40-9 | -- |
| E40 | S-(1-Methylpropyl) 3-methylbutanethioate | 4000.91±287.2 | 3678.46±109.56 | 3959.7±162.99 | C_9_H_18_OS | 1174 | 2432-91-9 | -- |
| E41 | Dimethyl adipate | 121.92±1.8 | 126.56±4.69 | 121±7.1 | C_8_H_14_O_4_ | 1243 | 627-93-0 | C14570 |
| E42 | 2-Methylbutyl 2-methylbutanoate | 685.94±101.23 | 614.39±15.03 | 661.3±14.37 | C_10_H_20_O_2_ | 1105 | 2445-78-5 | -- |
| E43 | Amyl isovalerate | 9.24±1.39 | 8.73±0.61 | 8.61±0.4 | C_10_H_20_O_2_ | 1110 | 25415-62-7 | -- |
| E44 | 3-Methylbutyl 2-methylbutanoate | 684.49±101.76 | 612.85±15.36 | 659.4±14.62 | C_10_H_20_O_2_ | 1101 | 27625-35-0 | -- |
| E45 | Propanoic acid, 2,2-dimethyl-, pentyl ester | 53.16±22.76a | 16.8±2.06b | 17.83±3.37b | C_10_H_20_O_2_ | 1063 | 2313-68-0 | -- |
| E46 | 2-Methylbutyl isovalerate | 216.43±21.51a | 29.83±6.3b | 33.96±0.86b | C_10_H_20_O_2_ | 1107 | 2445-77-4 | -- |
| E47 | Isoamyl isovalerate | 686.03±101.49 | 614.37±15.11 | 661.09±14.51 | C_10_H_20_O_2_ | 1104 | 659-70-1 | C12289 |
| E48 | Butanoic acid, 5-hexenyl ester | 388.06±38.37a | 344.18±21.57ab | 330.43±9.54b | C_10_H_18_O_2_ | 1183 | 108058-75-9 | -- |
| E49 | δ-Decalactone | 32.35±9.47b | 62.21±7.69a | 63.43±3.56a | C_10_H_18_O_2_ | 1497 | 705-86-2 | -- |
| E50 | (4Z)-4-Hexenyl butyrate | 343.24±5.67 | 354.26±12.14 | 345.99±15.67 | C_10_H_18_O_2_ | 1203 | 69727-41-9 | -- |
| E51 | cis-3-Hexenyl butanoate | 13.23±2.32 | 11.57±0.12 | 10.89±0.29 | C_10_H_18_O_2_ | 1187 | 16491-36-4 | -- |
| E52 | trans-3-Hexenyl butyrate | 67.58±9.33 | 59.98±7.72 | 57.87±3.37 | C_10_H_18_O_2_ | 1185 | 53398-84-8 | -- |
| E53 | (3Z)-3-Hexen-1-yl (2E)-2-butenoate | 2514.12±42.31 | 2585.57±129.03 | 2526.31±70.9 | C_10_H_16_O_2_ | 1199 | 65405-80-3 | -- |
| E54 | Ethinamate | 61.07±7.71b | 115.75±3.65a | 104.32±11.82a | C_9_H_13_NO_2_ | 1397 | 126-52-3 | C07832 |
| E55 | 2-Hydroxyethyl benzoate | 100.88±13.44b | 208.78±3.38a | 185.08±22.17a | C_9_H_10_O_3_ | 1402 | 94-33-7 | -- |
| E56 | Ethyl phenylacetate | 90.35±0.97 | 91.15±6.23 | 87.83±5.59 | C_10_H_12_O_2_ | 1247 | 101-97-3 | -- |
| E57 | Isopropyl benzoate | 3.59±0.33 | 3.48±0.37 | 3.51±0.33 | C_10_H_12_O_2_ | 1217 | 939-48-0 | -- |
| E58 | Allyl benzoate | 49.91±0.5 | 50.01±1.96 | 47.29±2.26 | C_10_H_10_O_2_ | 1254 | 583-04-0 | -- |
| E59 | Ethyl 2-hydroxy-4-methylpentanoate | 76.68±29.91a | 29.77±2.38b | 25.22±2.73b | C_8_H_16_O_3_ | 1060 | 10348-47-7 | -- |
| E60 | Propyl hexanoate | 10.83±1.25 | 10.47±0.91 | 10.52±1.2 | C_9_H_18_O_2_ | 1097 | 626-77-7 | -- |
| E61 | Ethyl heptanoate | 35.73±4.97 | 30.61±1.13 | 32.74±0.47 | C_9_H_18_O_2_ | 1098 | 106-30-9 | -- |
| E62 | 2-Methylbutyl isobutyrate | 1.77±0.8 | 2.42±0.32 | 2.49±0.15 | C_9_H_18_O_2_ | 1016 | 2445-69-4 | -- |
| E63 | Heptyl acetate | 207.61±19.82a | 24.81±1.84b | 27.84±1.38b | C_9_H_18_O_2_ | 1112 | 112-06-1 | -- |
| E64 | Isobutyl 2-methylbutyrate | 5.95±2.83 | 8.34±0.41 | 7.87±1.24 | C_9_H_18_O_2_ | 1004 | 2445-67-2 | -- |
| E65 | (2Z)-2-Penten-1-yl butanoate | 5.32±0.48c | 58.45±4.09b | 75.32±0.69a | C_9_H_16_O_2_ | 1091 | 42125-13-3 | -- |
| E66 | 2-Butenoic acid, 2-methyl-, 2-methylpropyl ester | 2709.56±400.8 | 2347.29±65.17 | 2521±58.44 | C_9_H_16_O_2_ | 1097 | 66917-61-1 | -- |
| E67 | 2-methyl-2E-butenyl methacrylate | 2299.1±332.34 | 2000.78±54.79 | 2141.67±49.91 | C_9_H_14_O_2_ | 1088 | 88142-95-4 | -- |
| E68 | Methyl 2-methylbenzoate | 9.98±2.52 | 9.81±1.44 | 10.01±0.98 | C_9_H_10_O_2_ | 1181 | 89-71-4 | -- |
| E69 | Ethyl benzoate | 132.72±4.19a | 110.02±2.45b | 114.1±4.29b | C_9_H_10_O_2_ | 1172 | 93-89-0 | -- |
| E70 | 2-Phenylethyl formate | 3.28±0.77 | 2.59±0.21 | 2.88±0.23 | C_9_H_10_O_2_ | 1178 | 104-62-1 | -- |
| E71 | Methyl phenylacetate | 7.77±1.73 | 7.76±0.28 | 7.23±1.39 | C_9_H_10_O_2_ | 1178 | 101-41-7 | -- |
| E72 | 1-(dimethylamino)propan-2-yl acetate | 68.83±16.54b | 319.19±21.49a | 328.45±11.05a | C_7_H_15_NO_2_ | 869 | 32188-28-6 | -- |
| E73 | Butyl isobutyrate | 4.76±0.89a | 2.38±0.37b | 3.35±0.25b | C_8_H_16_O_2_ | 898 | 97-87-0 | -- |
| E74 | Hexyl acetate | 1.61±0.31b | 3.96±0.66a | 4.67±0.27a | C_8_H_16_O_2_ | 1014 | 142-92-7 | -- |
| E75 | Isobutyl butyrate | 24.97±7.98a | 10.19±0.97b | 11.53±1.11b | C_8_H_16_O_2_ | 954 | 539-90-2 | -- |
| E76 | Pentyl acrylate | 212.5±54.26a | 112.63±16.93b | 114.52±4.76b | C_8_H_14_O_2_ | 974 | 2998-23-4 | -- |
| E77 | Phenyl acetate | 34.77±3.34 | 31.78±2.47 | 34.47±0.95 | C_8_H_8_O_2_ | 1067 | 122-79-2 | C00548 |
| E78 | Methyl benzoate | 4.92±0.38 | 4.57±0.83 | 4.46±0.73 | C_8_H_8_O_2_ | 1094 | 93-58-3 | C20645 |
| E79 | 2-Methylbutyl acetate | 93.32±19.61b | 217.6±1.46a | 224.51±19.71a | C_7_H_14_O_2_ | 880 | 624-41-9 | -- |
| E80 | Isoamyl acetate | 97.49±23.44b | 250.69±27.06a | 245.97±18.68a | C_7_H_14_O_2_ | 876 | 123-92-2 | C12296 |
| E81 | Methyl hexanoate | 4.51±0.23a | 2.47±0.39c | 3.32±0.16b | C_7_H_14_O_2_ | 925 | 106-70-7 | -- |
| E82 | Propyl butyrate | 4.82±0.62a | 2.49±0.12b | 3.03±0.49b | C_7_H_14_O_2_ | 899 | 105-66-8 | -- |
| E83 | Sotolone | 2713.78±402.49 | 2346.65±58.86 | 2524.49±59.52 | C_6_H_8_O_3_ | 1110 | 28664-35-9 | -- |
| E84 | Isobutyl acetate | 858±4.44 | 846.2±10.46 | 852.06±55.39 | C_6_H_12_O_2_ | 772 | 110-19-0 | -- |
| E85 | Ethyl butyrate | 0±0 | 7.97±1.01b | 9.56±0.93a | C_6_H_12_O_2_ | 804 | 105-54-4 | -- |
| E86 | Methyl 3-methyl-2-butenoate | 38.37±5.33a | 5.76±0.27b | 5.13±0.42b | C_6_H_10_O_2_ | 842 | 924-50-5 | -- |
| E87 | ε-Caprolactone | 118.07±17.37 | 98.54±3.95 | 107.45±1.8 | C_6_H_10_O_2_ | 1065 | 502-44-3 | C01880 |
| ***Esters*** |  | 33450.69±1332.05 | 32666.11±1147.06 | 32646.87±1306.4 |  |  |  |  |
| Alc1 | Balinol | 9.97±1.37 | 9.22±0.85 | 9.07±1.07 | C_14_H_24_O | 1559 | 28219-61-6 | -- |
| Alc2 | 1-Dodecanol | 7.59±1.46c | 112.84±4.12a | 87.52±7.13b | C_12_H_26_O | 1474 | 112-53-8 | C02277 |
| Alc3 | 2-Butyl-2-octenal | 8.36±0.79a | 5.94±0.47b | 8.18±1.02a | C_12_H_22_O | 1378 | 13019-16-4 | -- |
| Alc4 | Benzenemethanol, 4-methyl-.alpha.-(1-methyl-2-propenyl)-, (R*,R*)- | 288.91±36.74b | 617.32±13.34a | 544.07±66.47a | C_12_H_16_O | 1393 | 83173-76-6 | -- |
| Alc5 | 1-Undecanol | 10.19±1.3a | 5.91±0.29b | 7.86±1.87ab | C_11_H_24_O | 1371 | 112-42-5 | -- |
| Alc6 | 6-Undecanol | 10.02±2.35b | 17.41±1.05a | 19.96±3.28a | C_11_H_24_O | 1277 | 23708-56-7 | -- |
| Alc7 | 5-Undecanol | 0±0 | 28.84±3.06b | 35.46±2.71a | C_11_H_24_O | 1288 | 37493-70-2 | -- |
| Alc8 | 3,7-Dimethyl-1-octen-3,7-diol | 129.97±7.59 | 134.83±4.3 | 128.73±4.28 | C_10_H_20_O_2_ | 1237 | 29210-77-3 | -- |
| Alc9 | 2-[(4-Chlorophenyl)amino]ethanol | 2±0.64c | 80.72±1.83a | 62.39±4.72b | C_8_H_10_ClNO | 1514 | 2933-81-5 | -- |
| Alc10 | 2,6-Dimethylocta-3,7-diene-2,6-diol | 42.7±3.36 | 47.46±3.8 | 47.1±2.77 | C_10_H_18_O_2_ | 1193 | 13741-21-4 | -- |
| Alc11 | 2,6-Dimethyl-1-nonen-3-yn-5-ol | 0±0 | 44.59±2.72a | 37.41±1.58b | C_11_H_18_O | 1300 | 19780-98-4 | -- |
| Alc12 | Ethanol, 2-(4-ethylphenoxy)- | 0±0 | 4.43±0.4 | 4.16±1.22 | C_10_H_14_O_2_ | 1424 | 54411-10-8 | -- |
| Alc13 | 2-Methyl-4-phenyl-2-butanol | 2.83±1.13 | 2.78±0.08 | 3.14±0.97 | C_11_H_16_O | 1276 | 103-05-9 | -- |
| Alc14 | (5E)-2,6-Dimethyl-5,7-octadien-2-ol | 1072.22±61.33 | 1023.96±49.56 | 1015.6±34.48 | C_10_H_18_O | 1169 | 7643-60-9 | -- |
| Alc15 | Cyclohexanol, 2-methyl-3-(1-methylethenyl)-, (1.alpha.,2.alpha.,3.alpha.)- | 32.01±0.99 | 34.47±1.99 | 33.34±1.66 | C_10_H_18_O | 1196 | 54244-81-4 | -- |
| Alc16 | (+)-Santolina alcohol | 225.13±23.9a | 169.32±10.94b | 202.79±23.57ab | C_10_H_18_O | 1039 | 35671-15-9 | -- |
| Alc17 | Grandlure II | 21.22±5.68b | 35.22±0.72a | 33.15±1.74a | C_10_H_18_O | 1267 | 26532-23-0 | -- |
| Alc18 | Grandisol, (+/-)- | 41.39±9.8 | 38.49±2.39 | 41.98±2.96 | C_10_H_18_O | 1183 | 30820-22-5 | -- |
| Alc19 | 3,7-Dimethylocta-1,5,7-trien-3-ol | 121.29±10.46a | 42.34±1.53b | 43.21±0.62b | C_10_H_16_O | 1107 | 29957-43-5 | -- |
| Alc20 | 3,7-Dimethyl-1,5(E),7-octatrien-3-ol | 121.88±10.89a | 45.52±1.82b | 48.42±0.96b | C_10_H_16_O | 1106 | 20053-88-7 | -- |
| Alc21 | 4-Phenyl-2-butanol | 32.82±2.46ab | 34.33±0.37a | 30.95±0.21b | C_10_H_14_O | 1241 | 2344-70-9 | -- |
| Alc22 | 1,2-Octanediol | 63.64±2.97b | 69.58±1.87a | 68.95±2.88a | C_8_H_18_O_2_ | 1204 | 1117-86-8 | C14273 |
| Alc23 | 1-Nonanol | 2379.99±185.13b | 2431.61±127.87ab | 2682.04±108.09a | C_9_H_20_O | 1173 | 143-08-8 | C14696 |
| Alc24 | cis-6-Nonen-1-ol | 1210.33±92.61 | 1214.27±59.96 | 1308.62±58.35 | C_9_H_18_O | 1171 | 35854-86-5 | -- |
| Alc25 | trans-2-Nonen-1-ol | 3951.15±309.54 | 3625.15±101.76 | 3919.42±165.29 | C_9_H_18_O | 1169 | 31502-14-4 | -- |
| Alc26 | (E)-6-Nonen-1-ol | 99.61±5.83 | 96.9±6.48 | 99.86±7.19 | C_9_H_18_O | 1167 | 31502-19-9 | -- |
| Alc27 | Cyclooctanemethanol | 4.5±2.66c | 14.62±3.34b | 24.83±1.59a | C_9_H_18_O | 1073 | 3637-63-6 | -- |
| Alc28 | 1-Nonen-4-ol | 2359.35±348.38 | 2057.19±63.05 | 2229.85±52.81 | C_9_H_18_O | 1103 | 35192-73-5 | -- |
| Alc29 | Acetic acid--hex-4-en-1-ol (1/1) | 6.51±3.3 | 4.06±0.48 | 4.8±0.61 | C_8_H_14_O_2_ | 1020 | 72237-36-6 | -- |
| Alc30 | trans-2,cis-6-Nonadienol | 1198.47±91.81 | 1206.73±54.65 | 1298.86±55.97 | C_9_H_16_O | 1170 | 28069-72-9 | -- |
| Alc31 | 3-Phenylpropanol | 30.82±1.41 | 28.78±2.23 | 29.02±2.41 | C_9_H_12_O | 1233 | 122-97-4 | -- |
| Alc32 | 1-Octanol | 10.46±4.79c | 27.22±5.6b | 39.41±4.2a | C_8_H_18_O | 1072 | 111-87-5 | C00756 |
| Alc33 | 2-Ethylhexanol | 6.1±1.76 | 3.95±1.13 | 5.28±0.67 | C_8_H_18_O | 1030 | 104-76-7 | C02498 |
| Alc34 | Cyclopentanepropanol | 919.55±134.1 | 796.81±20.16 | 843.62±21.09 | C_8_H_16_O | 1102 | 767-05-5 | -- |
| Alc35 | 3,5-Dimethylcyclohexanol | 13.88±1.81 | 13.64±0.07 | 15.01±0.36 | C_8_H_16_O | 1030 | 5441-52-1 | -- |
| Alc36 | (E)-2-Octen-1-ol | 20.62±2.2a | 16.03±2.09b | 14.13±1.8b | C_8_H_16_O | 1067 | 18409-17-1 | -- |
| Alc37 | 1-Octen-3-ol | 43.24±20.07b | 66.73±14.26b | 179.43±33.56a | C_8_H_16_O | 982 | 3391-86-4 | C14272 |
| Alc38 | 2,6-Dimethylcyclohexanol | 117.88±8.24a | 36.63±2.47b | 35.84±0.72b | C_8_H_16_O | 1112 | 5337-72-4 | -- |
| Alc39 | 6-Methyl-5-hepten-2-ol | 35.86±11.21 | 32.52±9 | 47.56±5.22 | C_8_H_16_O | 994 | 1569-60-4 | C07288 |
| Alc40 | 2-Phenylethanol | 2.96±0.16 | 4.37±1.6 | 3.51±0.37 | C_8_H_10_O | 1116 | 22258 | C05853 |
| Alc41 | 1-Phenylethanol | 33.61±7.32a | 17.47±1.4b | 17.59±2.17b | C_8_H_10_O | 1061 | 98-85-1 | C07112 |
| Alc42 | 4-Methylbenzyl alcohol | 317.21±45.91 | 272.3±2.53 | 286.9±6.96 | C_8_H_10_O | 1106 | 589-18-4 | C06757 |
| Alc43 | 3-Mercapto-3-methylbutanol | 129.14±52.52a | 56.27±6.98b | 54.24±4.37b | C_5_H_12_OS | 970 | 34300-94-2 | -- |
| Alc44 | 3-Heptanol | 14.09±3.93b | 56.04±5.03a | 66.3±10.48a | C_7_H_16_O | 877 | 589-82-2 | -- |
| Alc45 | 1-Hexanol, 4-methyl- | 103.09±46.77a | 43.69±5.3b | 44.21±5.9b | C_7_H_16_O | 953 | 818-49-5 | -- |
| Alc46 | (2E,4E)-2,4-Heptadien-1-ol | 8.56±3.53 | 4.65±0.26 | 7.27±1.06 | C_7_H_12_O | 1004 | 33467-79-7 | -- |
| Alc47 | Benzyl alcohol | 45.63±7.32 | 57.86±1.29 | 64.4±14.82 | C_7_H_8_O | 1036 | 100-51-6 | C00556 |
| Alc48 | 2-Hexanol, (R)- | 993.55±7.97 | 1001.65±18.23 | 948.86±49.89 | C_6_H_14_O | 780 | 26549-24-6 | -- |
| Alc49 | 1-Hexanol | 2201.63±478.2b | 5565.25±129.23a | 5610.42±188.93a | C_6_H_14_O | 868 | 111-27-3 | -- |
| Alc50 | trans-2-Hexen-1-ol | 813.66±201.07c | 3736.09±67.17b | 4194.94±133.53a | C_6_H_12_O | 862 | 928-95-0 | -- |
| Alc51 | (2E)-3-Methyl-2,4-pentadien-1-ol | 72.09±7.37a | 40.19±10.01b | 46.32±7.91b | C_6_H_10_O | 836 | 1572-08-3 | -- |
| Alc52 | Glycerol | 3.16±0.51a | 1.62±0.22b | 2.1±0.25b | C_3_H_8_O_3_ | 967 | 56-81-5 | C00116 |
| Alc53 | 3-Methyl-2-buten-1-ol | 12.27±1.05b | 16.68±0.88a | 17.59±1.48a | C_5_H_10_O | 776 | 556-82-1 | C01390 |
| Alc54 | 3-Pentyn-1-ol | 2.01±0.21 | 2.3±0.08 | 2.31±0.5 | C_5_H_8_O | 778 | 10229-10-4 | -- |
| ***Alcohols*** |  | 19405.12±2088.88b | 25154.75±661.16a | 26657.97±889.74a |  |  |  |  |
| Ald1 | Myrac aldehyde | 7.54±1.55 | 8.05±2.43 | 9.76±0.66 | C_13_H_20_O | 1540 | 37677-14-8 | -- |
| Ald2 | (E)-2-Dodecenal | 3.05±0.6a | 0.87±0.18b | 1.23±0.26b | C_12_H_22_O | 1468 | 20407-84-5 | -- |
| Ald3 | (2E,6Z)-2,6-Dodecadienal | 57.85±4.97 | 47.1±1.06 | 49.1±9.88 | C_12_H_20_O | 1449 | 21662-13-5 | -- |
| Ald4 | Metaldehyde | 19±2.05 | 17.43±1.4 | 20.13±0.73 | C_8_H_16_O_4_ | 1160 | 108-62-3 | C18744 |
| Ald5 | (3Z)-1-(1-Ethoxyethoxy)-3-hexene | 311.25±44.23 | 259.27±8.22 | 278.86±6.31 | C_10_H_20_O_2_ | 1102 | 28069-74-1 | -- |
| Ald6 | Undecanal | 1.7±1.4c | 17.33±1.92b | 24.2±0.8a | C_11_H_22_O | 1307 | 112-44-7 | -- |
| Ald7 | 2-Undecenal | 0.65±0.37 | 1.14±0.38 | 0.69±0.31 | C_11_H_20_O | 1367 | 2463-77-6 | -- |
| Ald8 | 10-Undecenal | 0±0 | 90.6±3.97a | 77.03±2.98b | C_11_H_20_O | 1297 | 112-45-8 | -- |
| Ald9 | Lilac aldehyde, (2S,2'R,5'S)- | 87.44±9.99 | 77.29±4.49 | 84.41±3.96 | C_10_H_16_O_2_ | 1167 | 53447-48-6 | -- |
| Ald10 | Lilac aldehyde, (2R,2'R,5'S)- | 1554.24±123.62b | 1620±100.32ab | 1815.14±82.84a | C_10_H_16_O_2_ | 1169 | 53447-47-5 | -- |
| Ald11 | 3,4-Dimethoxybenzaldehyde | 673.75±80.21 | 693.96±28.94 | 619.69±45.29 | C_9_H_10_O_3_ | 1489 | 120-14-9 | C02201 |
| Ald12 | trans-4-Decen-1-al | 46.99±1.74 | 49.8±2.41 | 48.32±3.57 | C_10_H_18_O | 1198 | 65405-70-1 | -- |
| Ald13 | 2-Decenal, (2Z)- | 419.89±17.64 | 430.31±30.86 | 403.12±11.09 | C_10_H_18_O | 1252 | 2497-25-8 | -- |
| Ald14 | cis-7-Decenal | 720.42±18.77 | 722.18±25.58 | 679.58±33.76 | C_10_H_18_O | 1212 | 21661-97-2 | -- |
| Ald15 | Grandlure III | 11.5±2.02 | 10.29±1.21 | 12.43±0.51 | C_10_H_16_O | 1226 | 26532-24-1 | -- |
| Ald16 | 4-Isopropyl-3-cyclohexene-1-carbaldehyde | 117.63±6.83 | 113.94±2.22 | 115.68±5.02 | C_10_H_16_O | 1169 | 27841-22-1 | -- |
| Ald17 | 2,4-Decadienal, (2E,4Z)- | 0.8±0.01c | 48.75±2.88a | 42.16±0.66b | C_10_H_16_O | 1295 | 25152-83-4 | -- |
| Ald18 | (S)-Perillaldehyde | 2073.15±25.91 | 2125.32±90.26 | 2048.86±90.19 | C_10_H_14_O | 1207 | 18031-40-8 | -- |
| Ald19 | 3-(4-hydroxyphenyl)propanal | 3335.99±369.43 | 3383.62±181.3 | 3064.05±221.13 | C_9_H_10_O_2_ | 1490 | 20238-83-9 | C16706 |
| Ald20 | 2-Nonenal | 5.02±0.38a | 3.59±0.37b | 4.87±0.47a | C_9_H_16_O | 1161 | 2463-53-8 | -- |
| Ald21 | (E)-2-Nonenal | 1326.05±83.27 | 1223.76±50.99 | 1253.4±41.7 | C_9_H_16_O | 1162 | 18829-56-6 | -- |
| Ald22 | trans-4-Nonenal | 1434.63±209.27 | 1288.53±35.73 | 1343.82±42.3 | C_9_H_16_O | 1105 | 2277-16-9 | -- |
| Ald23 | cis-6-Nonenal | 1433.34±208.45 | 1286.05±35.25 | 1341.64±41.75 | C_9_H_16_O | 1101 | 2277-19-2 | -- |
| Ald24 | 4-Methoxybenzaldehyde | 116.16±2.59 | 118.23±3.88 | 110.71±4.6 | C_8_H_8_O_2_ | 1251 | 123-11-5 | C10761 |
| Ald25 | 3-Ethylbenzaldehyde | 115.03±4.69a | 3.76±0.29b | 1.87±0.13 | C_9_H_10_O | 1168 | 34246-54-3 | -- |
| Ald26 | Paraldehyde | 977.81±23.24 | 1004.77±30.92 | 1001.15±58.37 | C_6_H_12_O_3_ | 776 | 123-63-7 | C07834 |
| Ald27 | Cinnamaldehyde [NF] | 6.74±1.21 | 7.88±0.98 | 6.92±0.18 | C_9_H_8_O | 1274 | 104-55-2 | -- |
| Ald28 | 2-Ethylhexanal | 122.16±46.37a | 50.44±4.63b | 55.76±2.12b | C_8_H_16_O | 956 | 123-05-7 | -- |
| Ald29 | (E)-2-Octenal | 63.99±25.69a | 21.95±1.6b | 22.51±3.08b | C_8_H_14_O | 1060 | 2548-87-0 | C21138 |
| Ald30 | 2-Methylhept-2-enal | 49±7.93b | 57.32±3.26b | 79.2±4a | C_8_H_14_O | 990 | 30567-26-1 | -- |
| Ald31 | 2,5-Furandicarboxaldehyde | 0.89±0.13b | 1.09±0.14ab | 1.24±0.18a | C_6_H_4_O_3_ | 1076 | 823-82-5 | C20899 |
| Ald32 | 3-Hydroxybenzaldehyde | 9.39±2.39 | 11.82±0.44 | 12.05±0.97 | C_7_H_6_O_2_ | 1327 | 100-83-4 | C03067 |
| Ald33 | 3-Methylbenzaldehyde | 54.43±11.78a | 37.77±1.57b | 41.93±4.25ab | C_8_H_8_O | 1071 | 620-23-5 | C07209 |
| Ald34 | 2-Methylbenzaldehyde | 54.42±11.87a | 37.33±2.01b | 41.94±4.24ab | C_8_H_8_O | 1064 | 529-20-4 | C07214 |
| Ald35 | 4-Methylbenzaldehyde | 4.31±0.22 | 4.61±0.64 | 3.98±0.63 | C_8_H_8_O | 1079 | 104-87-0 | C06758 |
| Ald36 | Phenylacetaldehyde | 28.71±5.35a | 8.75±0.48b | 10.1±0.64b | C_8_H_8_O | 1049 | 122-78-1 | C00601 |
| Ald37 | 4-Heptenal | 7.36±1.14a | 3.64±0.4b | 4.37±0.49b | C_7_H_12_O | 899 | 62238-34-0 | -- |
| Ald38 | Cyclohexanecarboxaldehyde | 240.01±74.69a | 113.99±10.09b | 121.86±11.18b | C_7_H_12_O | 963 | 2043-61-0 | -- |
| Ald39 | Benzaldehyde | 126.28±11.35a | 56.53±6.62b | 67.28±4.15b | C_7_H_6_O | 960 | 100-52-7 | C00193 |
| Ald40 | Hexanal | 1830.49±129.87a | 12.4±0.82b | 8.42±1.32b | C_6_H_12_O | 801 | 66-25-1 | -- |
| Ald41 | (Z)-3-Hexenal | 58.03±5.81a | 4.95±0.17b | 3.79±0.53b | C_6_H_10_O | 800 | 6789-80-6 | C16310 |
| Ald42 | trans-2-Hexenal | 1035.22±70.03a | 412.32±81.41b | 421.77±55.85b | C_6_H_10_O | 854 | 6728-26-3 | C08497 |
| ***Aldehydes*** |  | 18431.04±516.96a | 15486.87±666.35b | 15355.82±722.76b |  |  |  |  |
| K1 | 6-Methyl-6-(5-methylfuran-2-yl)heptan-2-one | 10.21±1.68 | 9.87±0.34 | 9.89±0.98 | C_13_H_20_O_2_ | 1433 | 50464-95-4 | -- |
| K2 | 1-Pentanone, 1-(p-anisyl)-3-methyl- | 9.45±1.36 | 8.83±0.49 | 8.81±0.42 | C_13_H_18_O_2_ | 1552 | 66333-82-2 | -- |
| K3 | 1-(4-Methoxyphenyl)-4-methyl-1-penten-3-one | 3.63±0.33 | 3.41±0.41 | 2.99±0.76 | C_13_H_16_O_2_ | 1560 | 103-13-9 | -- |
| K4 | 3-Isopropyl-5-methyl-3,4-dihydro-1(2H)-naphthalenone | 3.07±0.38 | 3.07±0.08 | 3.29±0.36 | C_14_H_18_O | 1647 | 29107-42-4 | -- |
| K5 | 2-Tridecanone | 221.26±21.59 | 214.98±10.67 | 198.17±14.89 | C_13_H_26_O | 1496 | 593-08-8 | -- |
| K6 | 6,10-Dimethyl-2-undecanone | 12.95±1.58b | 10.36±0.68b | 17.18±2.23a | C_13_H_26_O | 1408 | 1604-34-8 | -- |
| K7 | 6,7-Dodecanedione | 357.32±37.7 | 359.3±14.8 | 327.35±23.77 | C_12_H_22_O_2_ | 1486 | 13757-90-9 | -- |
| K8 | Geranylacetone | 67.05±7.96 | 54.11±1.28 | 62.01±7.9 | C_13_H_22_O | 1452 | 689-67-8 | -- |
| K9 | Dihydro-α-ionone | 289.48±36.93b | 617.13±15.54a | 544.28±65.97a | C_13_H_22_O | 1406 | 31499-72-6 | -- |
| K10 | Geranylacetone | 70.47±7.24a | 56.37±1.72b | 63.67±7.69ab | C_13_H_22_O | 1453 | 3796-70-1 | -- |
| K11 | Dihydro-β-ionone | 47.04±4.47 | 47.05±1.62 | 46.73±3.99 | C_13_H_22_O | 1433 | 17283-81-7 | C03527 |
| K12 | (6E)-8-Methyl-5-(1-methylethyl)-6,8-nonadien-2-one | 2.71±0.45 | 2.9±0.02 | 2.98±0.62 | C_13_H_22_O | 1373 | 54868-48-3 | -- |
| K13 | Pseudoionone, (3E,5Z)- | 12.26±1.9a | 8.36±1.33b | 9.22±1.23b | C_13_H_20_O | 1535 | 13927-47-4 | -- |
| K14 | 4-(2,6,6-Trimethyl-1,3-cyclohexadien-1-yl)-2-butanone | 17.25±5.17 | 20.41±1.81 | 12.93±3.36 | C_13_H_20_O | 1424 | _20_483-36-7 | -- |
| K15 | (±)-α-Damascone | 234.61±26.66b | 498.87±9.55a | 441.34±52.18a | C_13_H_20_O | 1395 | 24720-09-0 | -- |
| K16 | Megastigmatrienone | 0.54±0.05 | 0.82±0.2 | 1.02±0.52 | C_13_H_18_O | 1473 | 38818-55-2 | -- |
| K17 | p-Mentha-8-thiol-3-one | 11.36±2.24 | 11.61±1.09 | 12.56±2.12 | C_10_H_18_OS | 1369 | 38462-22-5 | -- |
| K18 | 2,5-dihydroxy-6-propan-2-ylcyclohepta-2,4,6-trien-1-one | 8.17±0.52a | 4.64±0.28b | 4.55±0.44b | C_10_H_12_O_3_ | 1441 | 54755-56-5 | -- |
| K19 | 4',6'-Dihydroxy-2',3'-dimethylacetophenone | 4.79±0.76b | 6.79±0.47a | 6.52±0.64a | C_10_H_12_O_3_ | 1697 | 7743-14-8 | -- |
| K_20_ | 3-(Hydroxymethyl)-2-nonanone | 8.88±0.31c | 303.65±11.73b | 333.72±1.15a | C_10_H_20_O_2_ | 1093 | 67801-33-6 | -- |
| K21 | 2-Undecanone | 8.5±2.26c | 1167.82±58.37a | 1069.36±10.08b | C_11_H_22_O | 1294 | 112-12-9 | C01875 |
| K22 | 6-Methyl-3-(1-methylethyl)-7-oxabicyclo[4.1.0]heptan-2-one | 606.99±24.66 | 625.4±31.46 | 585.99±30.33 | C_10_H_16_O_2_ | 1256 | 5286-38-4 | -- |
| K23 | 2-Cyclohexen-1-one, 4-hydroxy-3-methyl-6-(1-methylethyl)-, trans- | 1.58±0.13a | 1.27±0.13b | 1.21±0.09b | C_10_H_16_O_2_ | 1432 | 55955-53-8 | -- |
| K24 | Piperitenone oxide | 0±0 | 7.99±1.13a | 6.46±0.65b | C_10_H_14_O_2_ | 1368 | 35178-55-3 | -- |
| K25 | Acetovanillone | 535.05±61.87 | 526.36±21.12 | 486.97±40.24 | C_9_H_10_O_3_ | 1487 | 498-02-2 | C11380 |
| K26 | Paeonol | 1.13±0.09b | 1.34±0.21ab | 1.45±0.14a | C_9_H_10_O_3_ | 1438 | 552-41-0 | C10712 |
| K27 | 4-(4′-Hydroxyphenyl)-2-butanone | 17.87±3.03 | 14.85±0.73 | 15.17±1.23 | C_10_H_12_O_2_ | 1553 | 5471-51-2 | -- |
| K28 | 2-Hydroxy-1-phenylbutan-1-one | 94.38±11.47b | 205.63±2.13a | 180.89±25.05a | C_10_H_12_O_2_ | 1390 | 16183-46-3 | -- |
| K29 | 4′-Methoxypropiophenone | 4.55±2.21 | 3.08±0.92 | 5.14±0.44 | C_10_H_12_O_2_ | 1513 | 121-97-1 | -- |
| K30 | 4-Methoxyphenylacetone | 287.38±36.85b | 615.53±13.63a | 542.79±66.35a | C_10_H_12_O_2_ | 1385 | 122-84-9 | -- |
| K31 | Valerophenone | 3.44±0.33 | 3.92±0.44 | 3.86±0.64 | C_11_H_14_O | 1374 | 1009-14-9 | -- |
| K32 | 2,2,5-Trimethylhexane-3,4-dione | 4.32±0.57 | 3.91±0.51 | 4.34±0.71 | C_9_H_16_O_2_ | 1039 | 20633-03-8 | -- |
| K33 | Methyl 2,2,3-trimethylcyclopentyl ketone | 2239.03±328.42 | 2057.25±60.11 | 2198.9±52.6 | C_10_H_18_O | 1092 | 17983-22-1 | -- |
| K34 | 4-Isopropyl-1,3-cyclohexanedione | 617.67±12.39 | 630.96±28.82 | 591.71±27.5 | C_9_H_14_O_2_ | 1258 | 62831-62-3 | -- |
| K35 | 3-Isopropylidene-5-methyl-hex-4-en-2-one | 918.79±133.76 | 794.56±20.36 | 841.78±20.73 | C_10_H_16_O | 1099 | 64149-32-2 | -- |
| K36 | 1-(1,3-Dimethyl-3-cyclohexen-1-yl)ethanone | 27.24±10.29 | 19.38±1.54 | 23.83±1.41 | C_10_H_16_O | 1161 | 51733-68-7 | -- |
| K37 | 2-Acetyl-4,4-dimethyl-cyclopent-2-enone | 2444.79±135.41 | 2543.91±69.34 | 2458.44±162.9 | C_9_H_12_O_2_ | 1213 | 81979-96-6 | -- |
| K38 | 2′,4′-Dihydroxyacetophenone | 7.14±1.66 | 10.4±0.61 | 10.15±2.84 | C_8_H_8_O_3_ | 1276 | 89-84-9 | C03663 |
| K39 | 4,7,7-Trimethylbicyclo[3.2.0]hept-3-en-6-one | 373.04±49.05 | 321.93±7.5 | 334.13±8.32 | C_10_H_14_O | 1108 | 4613-37-0 | -- |
| K40 | (E)-ocimenone | 119.66±6.93 | 122.55±10.31 | 117.57±3.92 | C_10_H_14_O | 1239 | 33746-72-4 | -- |
| K41 | 6-Camphenone | 652.32±89.6 | 581.04±14.1 | 605.9±18.36 | C_10_H_14_O | 1114 | 55659-42-2 | -- |
| K42 | 2′,4′-Dimethylacetophenone | 10.86±0.47b | 9.73±0.21a | 10.57±0.45b | C_10_H_12_O | 1253 | 89-74-7 | -- |
| K43 | 1-Phenyl-1,2-propanedione | 14.39±0.91a | 13.3±0.79ab | 12.04±0.6b | C_9_H_8_O_2_ | 1183 | 579-07-7 | C17268 |
| K44 | Isobutyl ketone | 118.27±45.33a | 56.13±5.43b | 56.11±4.33b | C_9_H_18_O | 952 | 108-83-8 | -- |
| K45 | 4,6-Dimethyl-2-heptanone | 28.13±2.9a | 17.72±0.33b | 20.36±1.54b | C_9_H_18_O | 1045 | 19549-80-5 | -- |
| K46 | 2,5-Octanedione | 7.48±3.32b | 5.77±1.52b | 14.77±3.06a | C_8_H_14_O_2_ | 984 | 3214-41-3 | -- |
| K47 | 2,2,6-Trimethylcyclohexanone | 22.92±4.52a | 15.85±1.21b | 16.7±0.35b | C_9_H_16_O | 1036 | 2408-37-9 | -- |
| K48 | 3-Nonen-5-one | 15.32±3.48 | 12.04±0.6 | 13.64±0.14 | C_9_H_16_O | 1052 | 82456-34-6 | -- |
| K49 | 2-Methyl-3-isopropylcyclopentanone | 9.64±0.7 | 8.49±0.29 | 9.03±0.64 | C_9_H_16_O | 1028 | 54549-81-4 | -- |
| K50 | 7-Methyl-3-octen-2-one, (3E)- | 105.15±15.17 | 90.04±3.36 | 94.56±3.14 | C_9_H_16_O | 1113 | 33046-81-0 | -- |
| K51 | 8-Nonen-2-one | 10.37±2.43c | 302.12±12.25b | 341.02±0.95a | C_9_H_16_O | 1085 | 5009-32-5 | -- |
| K52 | β-Isophorone | 40.38±5.83 | 41.31±1.6 | 45.8±2.52 | C_9_H_14_O | 1044 | 471-01-2 | -- |
| K53 | Isophorone | 5.4±0.6 | 5.19±2.34 | 5.35±0.12 | C_9_H_14_O | 1124 | 78-59-1 | C14743 |
| K54 | 3-Hydroxyacetophenone | 47.65±4.43 | 46.9±1.09 | 47.46±4.13 | C_8_H_8_O_2_ | 1439 | 121-71-1 | -- |
| K55 | p-Methylacetophenone | 23.4±2.98 | 21.32±0.72 | 20.41±1.17 | C_9_H_10_O | 1183 | 122-00-9 | -- |
| K56 | Phenylacetone | 16.69±4.01 | 19.46±0.99 | 20.56±0.74 | C_9_H_10_O | 1124 | 103-79-7 | C15512 |
| K57 | 2′-Methylacetophenone | 12.26±2.61a | 7.66±0.79b | 8.04±1.03b | C_9_H_10_O | 1173 | 577-16-2 | -- |
| K58 | 2-Octanone | 0±0 | 11.34±0.9b | 26.08±3.42a | C_8_H_16_O | 991 | 111-13-7 | -- |
| K59 | 2,2-Dimethyl-3-hexanone | 872.18±195.8b | 3799.41±169.7a | 4004.82±106.94a | C_8_H_16_O | 868 | 5405-79-8 | -- |
| K60 | (E)-5-Methyl-2-hepten-4-one | 94.82±41.57a | 40.74±3.41b | 40.13±5.19b | C_8_H_14_O | 972 | 102322-83-8 | -- |
| K61 | 6-Methyl-6-hepten-2-one | 52.23±5.23a | 35.34±2.64b | 37.1±1.82b | C_8_H_14_O | 966 | 10408-15-8 | -- |
| K62 | 3-Octen-2-one, (E)- | 131.7±11.11a | 89.26±6.93b | 105.19±12.08b | C_8_H_14_O | 1035 | 18402-82-9 | -- |
| K63 | 3-Octen-2-one | 51.47±2.67a | 36.16±0.73b | 41.05±3.69b | C_8_H_14_O | 1040 | 1669-44-9 | -- |
| K64 | 3,5-Octadien-2-one | 8.86±1.03b | 9.71±0.8ab | 11.22±0.41a | C_8_H_12_O | 1091 | 38284-27-4 | -- |
| K65 | Acetophenone | 11.9±3.47 | 7.89±0.88 | 8.19±0.69 | C_8_H_8_O | 1065 | 98-86-2 | C07113 |
| K66 | 2-Heptanone | 0±0 | 145.78±4.97b | 165.57±5.62a | C_7_H_14_O | 895 | 110-43-0 | C08380 |
| K67 | 2,2,3-Trimethyl cyclobutanone | 451.3±62.13a | 71.97±2.39b | 61.11±3.91b | C_7_H_12_O | 847 | 1449-49-6 | -- |
| K68 | 2-Hexanone | 243.13±18.37 | 0±0 | 0±0 | C_6_H_12_O | 792 | 591-78-6 | -- |
| K69 | Mesityl oxide | 56.25±3.59a | 1.58±0.4b | 1.58±0.07b | C_6_H_10_O | 798 | 141-79-7 | -- |
| K70 | 4-Hexen-3-one | 1382.32±195.52a | 189.59±9.75b | 175.1±10.94b | C_6_H_10_O | 855 | 2497-21-4 | -- |
| ***Ketones*** |  | 14201.85±855.13b | 17613.35±449.83a | 17608.88±735.97a |  |  |  |  |
| H1 | Eicosane | 30.48±8.73 | 21.41±12.51 | 35.31±3.95 | C_20_H_42_ | 2000 | 112-95-8 | -- |
| H2 | Pristane | 7±0.18 | 8.38±4.54 | 11.62±0.62 | C_19_H_40_ | 1690 | 1921-70-6 | -- |
| H3 | 1-Nonadecene | 10.29±1.33ab | 8.33±5.28b | 15.17±1.01a | C_19_H_38_ | 1893 | 18435-45-5 | -- |
| H4 | 2-Methylheptadecane | 14.86±0.76 | 12.29±8.09 | 17.91±2.2 | C_18_H_38_ | 1765 | 1560-89-0 | -- |
| H5 | Heptadecane, 3-methyl- | 2.99±0.38 | 3.1±2.08 | 4.93±1.2 | C_18_H_38_ | 1769 | 6418-44-6 | -- |
| H6 | Heptadecane, 7-methyl- | 4.04±0.24 | 2.86±1.59 | 4.36±0.19 | C_18_H_38_ | 1740 | 20959-33-5 | -- |
| H7 | Dispiro[1,3-dioxolane-2,2'-bicyclo[2.2.1]heptane-3',2''-(1'',3''-dioxolane)], 4',7',7'-trimethyl- | 2.47±0.43 | 2.81±1.3 | 3.94±0.52 | C_14_H_22_O_4_ | 1694 | 24022-14-8 | -- |
| H8 | 2-Methylhexadecane | 11.03±0.96 | 8.7±4.87 | 11.1±0.6 | C_17_H_36_ | 1664 | 1560-92-5 | -- |
| H9 | Pentadecane, 2-methyl- | 4.46±0.99 | 4.75±1.96 | 6.25±0.38 | C_16_H_34_ | 1563 | 1560-93-6 | -- |
| H10 | 5,8-Diethyldodecane | 6.45±1.51 | 6±2.07 | 7.84±0.42 | C_16_H_34_ | 1572 | 24251-86-3 | -- |
| H11 | Pentadecane, 4-methyl- | 6.05±1.75 | 6.24±2.54 | 8.43±0.86 | C_16_H_34_ | 1556 | 2801-87-8 | -- |
| H12 | Hexadecane | 7.24±1.7 | 7.26±2.76 | 9.97±0.82 | C_16_H_34_ | 1600 | 544-76-3 | -- |
| H13 | (5Z,13Z)-tricyclo[8.6.0.02,9]hexadeca-5,13-diene | 0±0 | 15.35±2.21 | 18.45±1.81 | C_16_H_24_ | 1687 | 61277-44-9 | -- |
| H14 | Pentadecane | 101.32±33.66b | 267.98±27.91a | 253.5±11.53a | C_15_H_32_ | 1500 | 629-62-9 | C08388 |
| H15 | 1-Pentadecene | 1274.51±124.14 | 1284.19±68.61 | 1165.91±80.63 | C_15_H_30_ | 1492 | 13360-61-7 | -- |
| H16 | (1S,5S)-2-Methyl-5-((R)-6-methylhept-5-en-2-yl)bicyclo[3.1.0]hex-2-ene | 37.48±5.38b | 73.37±3.57a | 65.06±6.42a | C_15_H_24_ | 1394 | 159407-35-9 | -- |
| H17 | (2S,6R,7S,8E)-(+)-2,7-Epoxy-4,8-megastigmadiene | 4.06±0.84b | 5.51±0.51a | 5.59±0.39a | C_13_H_20_O | 1325 | 108342-25-2 | -- |
| H18 | 8,8-Dimethyl-9-methylidenecycloundeca-1,5-diene | 4671.19±490.89 | 4676.03±327.73 | 4236.25±221.98 | C_14_H_22_ | 1485 | 62338-54-9 | -- |
| H19 | 6,6-Dimethylundecane | 5.36±1.41 | 4.81±1.22 | 3.67±0.58 | C_13_H_28_ | 1229 | 17312-76-4 | -- |
| H20 | 5-Methyldodecane | 277.27±3.79 | 279.01±17.71 | 266.94±13.75 | C_13_H_28_ | 1255 | 17453-93-9 | -- |
| H21 | 2,6-Dimethylundecane | 35.1±4.64a | 11.23±1.78b | 11.42±0.5b | C_13_H_28_ | 1210 | 17301-23-4 | -- |
| H22 | 2,9-Dimethylundecane | 5.34±1.35 | 4.75±1.17 | 3.71±0.77 | C_13_H_28_ | 1233 | 17301-26-7 | -- |
| H23 | Tridecane | 5.3±4.96b | 85.7±9.54a | 77.39±2.73a | C_13_H_28_ | 1300 | 629-50-5 | C13834 |
| H24 | 4,4-Dimethyl-undecane | 2.86±0.38 | 2.8±0.37 | 2.09±0.68 | C_13_H_28_ | 1229 | 17312-68-4 | -- |
| H25 | 4,6-Dimethylundecane | 99.29±14.16b | 118.93±5.9a | 108.05±5.48ab | C_13_H_28_ | 1196 | 17312-82-2 | -- |
| H26 | 3,4-Dimethylundecane | 271.24±13.16 | 266.89±22.2 | 263.24±11.54 | C_13_H_28_ | 1247 | 17312-78-6 | -- |
| H27 | 1-Tridecene | 1.32±0.89b | 5.64±0.41a | 7.24±1.88a | C_13_H_26_ | 1292 | 2437-56-1 | -- |
| H28 | 1-Tridecyne | 0±0 | 49.69±1.95a | 42.87±0.7b | C_13_H_24_ | 1297 | 26186-02-7 | -- |
| H29 | 2,6-Dimethyldecane | 58.96±13.5 | 61.14±2.81 | 63.61±0.71 | C_12_H_26_ | 1112 | 13150-81-7 | -- |
| H30 | Dodecane | 98.78±14.54 | 114.04±3.26 | 109.66±7.23 | C_12_H_26_ | 1200 | 112-40-3 | C08374 |
| H31 | 2,2,4,6,6-Pentamethylheptane | 7.68±0.36c | 51.78±3.9b | 119.11±13.78a | C_12_H_26_ | 990 | 13475-82-6 | -- |
| H32 | 1-Dodecene | 46.26±9.13 | 50.47±7.23 | 47.45±1.22 | C_12_H_24_ | 1190 | 112-41-4 | -- |
| H33 | (Z)-3-methylundec-4-ene | 62.92±4.82 | 59.81±1.04 | 65.29±5.66 | C_12_H_24_ | 1158 | 74645-87-7 | -- |
| H34 | 1,1-Dimethoxyheptane | 6.01±1.07ab | 4.64±1.41b | 8.79±2.72a | C_9_H_20_O_2_ | 1072 | 10032-05-0 | -- |
| H35 | (Z)-1-(1-methoxyethoxy)hex-3-ene | 1.77±0.34 | 1.71±0.38 | 1.45±0.09 | C_9_H_18_O_2_ | 1012 | 54340-96-4 | -- |
| H36 | Decane, 5-methyl- | 51.84±23.24 | 24.8±3.01 | 26.24±4.18 | C_11_H_24_ | 1057 | 13151-35-4 | -- |
| H37 | 2,6-Dimethylnonane | 0.89±0.5 | 0.92±0.31 | 1.08±0.16 | C_11_H_24_ | 1018 | 17302-28-2 | -- |
| H38 | Nonane, 4,5-dimethyl- | 115.19±5.83a | 72.7±1.37c | 85.77±8.5b | C_11_H_24_ | 1046 | 17302-23-7 | -- |
| H39 | 2,3,3-Trimethyloctane | 612.1±91.55a | 351.55±24.35b | 411.84±11.17b | C_11_H_24_ | 966 | 62016-30-2 | -- |
| H40 | Undecane | 236.88±35.74 | 237.66±3.88 | 255.5±10.78 | C_11_H_24_ | 1100 | 1120-21-4 | -- |
| H41 | 3,7-Dimethylnonane | 4.26±0.32 | 3.49±0.74 | 4.49±1.03 | C_11_H_24_ | 1038 | 17302-32-8 | -- |
| H42 | 1-Undecyne | 25.17±3.61ab | 23.32±0.9b | 29.16±0.73a | C_11_H_20_ | 1095 | 2243-98-3 | -- |
| H43 | 1,10-Undecadiene | 1425.01±208.09 | 1272.39±36.27 | 1347.78±29.32 | C_11_H_20_ | 1095 | 13688-67-0 | -- |
| H44 | 2-ethenyl-1,3,3-trimethylcyclohexene | 8.93±1.04 | 8.19±0.51 | 8.46±0.71 | C_11_H_18_ | 1105 | 5293-90-3 | -- |
| H45 | 2,4-dimethyl-1-prop-1-en-2-ylcyclohexene | 672.37±94.08 | 595.65±16.4 | 613.56±13.39 | C_11_H_18_ | 1108 | 56763-60-1 | -- |
| H46 | 2-Methyl-7-exo-vinylbicyclo[4.2.0]oct-1(2)-ene | 94.45±12.56 | 83.26±1.94 | 87.01±2.69 | C_11_H_16_ | 1112 | 107914-89-6 | -- |
| H47 | 3-Ethyl-3-methylheptane | 120.02±45.71a | 57.59±5.25b | 56.83±5b | C_10_H_22_ | 953 | 17302-01-1 | -- |
| H48 | cis-2,6-Dimethyl-2,6-octadiene | 7.16±2.89 | 4.1±0.77 | 6.56±1.44 | C_10_H_18_ | 985 | 2492-22-0 | -- |
| H49 | 1,6-Dimethyl-1,5-cyclooctadiene | 496.33±73.17 | 433.81±9.46 | 458.42±11.54 | C_10_H_16_ | 1103 | 3760-13-2 | -- |
| H50 | 1,5-Cyclooctadiene, 3,4-dimethyl- | 2.28±0.8 | 2.26±0.3 | 2.13±0.67 | C_10_H_16_ | 1046 | 21284-05-9 | -- |
| H51 | β-Thujene | 5.04±1.04a | 2.3±0.1b | 3.04±0.2b | C_10_H_16_ | 966 | 28634-89-1 | C21702 |
| H52 | Hexane, 3-ethyl-2-methyl- | 655.77±97.53a | 99.27±15.66b | 75.18±10.46b | C_9_H_20_ | 845 | 16789-46-1 | -- |
| H53 | trans-3,4-Epoxyoctane | 874.79±192.57b | 3725.37±167.08a | 4044.03±115.55a | C_8_H_16_O | 869 | 28180-72-5 | -- |
| H54 | 3-Ethyl-2-methyl-1,3-hexadiene | 31.65±2.04a | 26.93±0.71b | 29.82±1.26a | C_9_H_16_ | 1031 | 61142-36-7 | -- |
| H55 | Bicyclo(3.3.1)non-2-ene | 174.39±24.79a | 101.22±5.96b | 116.95±1.9b | C_9_H_14_ | 964 | 6671-66-5 | -- |
| H56 | 2-Hexene, 6-methoxy-, (2E)- | 349.2±23.63a | 2.27±0.17b | 0±0 | C_7_H_14_O | 801 | 134777-60-9 | -- |
| ***Hydrocarbons*** |  | 13145.11±235.42b | 14716.62±698.96a | 14747.42±564.73a |  |  |  |  |
| Ar1 | (5-Bromopentyl)benzene | 4842.57±477.55 | 4882.26±280.22 | 4442.74±322.11 | C_11_H_15_Br | 1487 | 14469-83-1 | -- |
| Ar2 | Asarone | 7.27±2.59 | 4.2±0.77 | 6.95±1.65 | C_12_H_16_O_3_ | 1678 | 2883-98-9 | C17846 |
| Ar3 | (1-Methyl-1-propylpentyl)benzene | 3380.53±356.48 | 3422.02±177.82 | 3092.7±216.06 | C_15_H_24_ | 1504 | 54932-91-1 | -- |
| Ar4 | Myristicin | 94.5±33.22 | 66.04±19.34 | 92.24±7.18 | C_11_H_12_O_3_ | 1520 | 607-91-0 | C10480 |
| Ar5 | Geosmin | 11.75±1.21 | 12.65±1.01 | 10.97±0.3 | C_12_H_22_O | 1430 | 19700-21-1 | C16286 |
| Ar6 | (1-Nitropropyl)benzene | 6.68±0.7 | 8.34±0.6 | 8.33±1.17 | C_9_H_11_NO_2_ | 1310 | 5279-14-1 | -- |
| Ar7 | 1,3,5-Triethylbenzene | 7.67±0.27 | 7.71±0.09 | 7.61±0.29 | C_12_H_18_ | 1218 | 102-25-0 | -- |
| Ar8 | 1H-Indene, 2,3-dihydro-4-propyl- | 6.16±1.07 | 6.68±0.15 | 6.37±0.18 | C_12_H_16_ | 1359 | 92013-16-6 | -- |
| Ar9 | 3,4-Dichlorotoluene | 2.46±0.31 | 1.86±0.45 | 2.13±0.18 | C_7_H_6_Cl2 | 1125 | 95-75-0 | -- |
| Ar10 | 1,4-Dimethylnaphthalene | 17.46±1.19 | 19.43±0.44 | 19.04±1.67 | C_12_H_12_ | 1436 | 571-58-4 | -- |
| Ar11 | 1,8-Dimethylnaphthalene | 31.47±2.18a | 25.11±3.2b | 30.94±3.25a | C_12_H_12_ | 1472 | 569-41-5 | C14703 |
| Ar12 | Biphenyl | 10.37±0.82b | 12.17±0.3a | 11.54±0.79ab | C_12_H_10_ | 1381 | 92-52-4 | C06588 |
| Ar13 | 3,4-Dimethoxytoluene | 10.29±0.89b | 8.97±0.81b | 11.93±0.19a | C_9_H_12_O_2_ | 1233 | 494-99-5 | -- |
| Ar14 | 4a-methyl-2,3,4,5,6,7-hexahydro-1H-naphthalene | 1.93±0.2 | 1.93±0.3 | 1.97±0.22 | C_11_H_18_ | 1185 | 13943-77-6 | -- |
| Ar15 | Benzene, (1-methoxypropyl)- | 65.58±8.72 | 61.76±1.9 | 63.76±1.83 | C_10_H_14_O | 1104 | 59588-12-4 | -- |
| Ar16 | Pentamethylbenzene | 10.34±0.78 | 10±0.77 | 10.54±0.64 | C_11_H_16_ | 1260 | 700-12-9 | -- |
| Ar17 | trans-Anethole | 4.57±0.64b | 5.13±0.22ab | 5.73±0.43a | C_10_H_12_O | 1283 | 4180-23-8 | -- |
| Ar18 | 1-Methylnaphthalene | 19±2.67 | 22.33±0.33 | 23.01±2.42 | C_11_H_10_ | 1307 | 90-12-0 | C14082 |
| Ar19 | 2-Methylnaphthalene | 0±0 | 3.93±0.12b | 3.64±0.07a | C_11_H_10_ | 1297 | 91-57-6 | C14098 |
| Ar20 | 1-Ethyl-4-methoxybenzene | 65.59±8.66 | 62.83±2.76 | 64.12±1.93 | C_9_H_12_O | 1110 | 1515-95-3 | -- |
| Ar21 | Butylbenzene | 29.44±6.26a | 8.73±0.24b | 10±0.55b | C_10_H_14_ | 1054 | 104-51-8 | C18150 |
| Ar22 | 1,4-Dimethyl-2-ethylbenzene | 15±4.89 | 17.33±2.01 | 18.23±1.76 | C_10_H_14_ | 1076 | 1758-88-9 | -- |
| Ar23 | Durene | 2.32±0.4b | 4.15±0.69a | 5.01±0.77a | C_10_H_14_ | 1115 | 95-93-2 | C14534 |
| Ar24 | p-Cymene | 120.59±17.3a | 95.64±2.69b | 111.91±6.92ab | C_10_H_14_ | 1027 | 99-87-6 | C06575 |
| Ar25 | 1-Ethyl-3,5-dimethylbenzene | 35.14±11.14 | 32.08±1.63 | 33.06±3.05 | C_10_H_14_ | 1059 | 934-74-7 | -- |
| Ar26 | 4-Methoxystyrene | 18.71±0.28a | 16.88±0.55b | 18.83±0.85a | C_9_H_10_O | 1156 | 637-69-4 | -- |
| Ar27 | Chavicol | 37.63±1.83 | 38.06±1.68 | 35.48±2.42 | C_9_H_10_O | 1255 | 501-92-8 | C16930 |
| Ar28 | 1-Methyl-2-phenylcyclopropane | 5.94±1.25 | 5.43±0.45 | 4.92±0.67 | C_10_H_12_ | 1056 | 3145-76-4 | -- |
| Ar29 | p-Cymenene | 31.45±1.34b | 35.99±1.3a | 39±2.54a | C_10_H_12_ | 1090 | 1195-32-0 | -- |
| Ar30 | m-Cymenene | 35.92±2.27b | 42.14±0.92a | 44.63±3.59a | C_10_H_12_ | 1082 | 1124-20-5 | -- |
| Ar31 | Naphthalene | 198.36±17.78 | 223.88±23.38 | 207.05±11.08 | C_10_H_8_ | 1182 | 91-20-3 | C00829 |
| Ar32 | 1,2,4-Trimethylbenzene | 7.97±1.33 | 7.93±0.6 | 8.44±0.45 | C_9_H_12_ | 990 | 95-63-6 | C14533 |
| Ar33 | 1-Ethyl-3-methylbenzene | 138.55±17.28a | 60.77±3.96c | 82.28±4.53b | C_9_H_12_ | 957 | 620-14-4 | C14522 |
| Ar34 | 1-Ethyl-2-methylbenzene | 145.68±22.26a | 66.23±5.53b | 80.26±3.6 | C_9_H_12_ | 971 | 611-14-3 | C14572 |
| Ar35 | Mesitylene | 145.07±22.1a | 65.83±5.02b | 80.29±3.56b | C_9_H_12_ | 972 | 108-67-8 | C14508 |
| Ar36 | Indane | 18.41±2.36a | 14.34±0.63b | 16.46±0.96ab | C_9_H_10_ | 1029 | 496-11-7 | -- |
| Ar37 | Methylphenylacetylene | 1.9±0.29 | 2.42±0.9 | 1.43±0.15 | C_9_H_8_ | 1052 | 673-32-5 | -- |
| Ar38 | o-Xylene | 12.67±4.08 | 8.26±0.67 | 9.88±0.6 | C_8_H_10_ | 888 | 95-47-6 | C07212 |
| Ar39 | p-Xylene | 26.92±6.73a | 17.94±0.52b | 21.09±1.48ab | C_8_H_10_ | 865 | 106-42-3 | C06756 |
| Ar40 | Ethylbenzene | 9.76±1.19a | 6.41±0.27b | 7.6±1.11b | C_8_H_10_ | 855 | 100-41-4 | C07111 |
| Ar41 | Styrene | 7.85±0.93a | 5.52±0.58b | 6.12±0.15b | C_8_H_8_ | 893 | 100-42-5 | C07083 |
| ***Aromatics*** |  | 9641.49±819.64 | 9421.28±512.71 | 8758.24±596.83 |  |  |  |  |
| Ac1 | Myristoleic acid | 2.55±0.39a | 1.66±0.53b | 3.04±0.17a | C_14_H_26_O_2_ | 1764 | 544-64-9 | C08322 |
| Ac2 | 2-Fluoro-4,5-dimethoxybenzoic acid | 0±0 | 2.08±0.06a | 1.75±0.08b | C_9_H_9_FO_4_ | 1503 | 79474-35-4 | -- |
| Ac3 | cis-5-Dodecenoic acid | 4.54±0.61 | 4.98±0.77 | 5.47±0.78 | C_12_H_22_O_2_ | 1561 | 2430-94-6 | -- |
| Ac4 | (Z)-8-methylnon-6-enoic acid | 11.89±1.8 | 12.14±0.89 | 14.04±1.22 | C_10_H_18_O_2_ | 1373 | 21382-25-2 | C18202 |
| Ac5 | 2,6-Dihydroxybenzoic acid | 2.31±0.5b | 3.15±0.38a | 2.75±0.12ab | C_7_H_6_O_4_ | 1397 | 303-07-1 | C21298 |
| Ac6 | Hydroxyphenylacetic acid | 17.34±2.45 | 14.83±0.35 | 14.84±1.33 | C_8_H_8_O_3_ | 1551 | 156-38-7 | C00642 |
| Ac7 | Phenoxyacetic acid | 32.2±4.84b | 69.38±2.45a | 61.05±7.34a | C_8_H_8_O_3_ | 1389 | 122-59-8 | C02181 |
| Ac8 | p-Tolylacetic acid | 99.4±15.02 | 103.83±2.27 | 104.49±10.54 | C_9_H_10_O_2_ | 1363 | 622-47-9 | -- |
| Ac9 | Oct-2-enoic acid | 4254.51±334.23 | 3798.41±95.54 | 4087.86±173.59 | C_8_H_14_O_2_ | 1181 | 1470-50-4 | -- |
| Ac10 | 4-Hydroxybenzoic acid | 3003.61±327.36 | 3047.33±166.66 | 2750.96±198.77 | C_7_H_6_O_3_ | 1502 | 99-96-7 | C00156 |
| Ac11 | 3-Pentenoic acid, 2,2-dimethyl- | 8.69±2.85b | 7.69±1.82b | 13.38±1.66a | C_7_H_12_O_2_ | 997 | 16642-52-7 | -- |
| Ac12 | Benzoic acid | 133.29±4.02a | 110.61±2.91b | 114.4±4.19b | C_7_H_6_O_2_ | 1177 | 65-85-0 | C00180 |
| Ac13 | Isocaproic acid | 107.62±45.12a | 45.88±5.08b | 45.85±1.98b | C_6_H_12_O_2_ | 949 | 646-07-1 | C21399 |
| ***Acids*** |  | 7677.96±48.09 | 7221.96±271.64 | 7219.86±389.45 |  |  |  |  |
| N1 | Dodecanenitrile | 360.31±39.68 | 363.07±20.31 | 329.8±22.36 | C_12_H_23_N | 1490 | 2437-25-4 | -- |
| S1 | Benzyl isothiocyanate | 1.76±0.4b | 5.77±1.1a | 5.58±0.19a | C_8_H_7_NS | 1367 | 622-78-6 | C03098 |
| N2 | 2-Nonenenitrile | 52.61±5.16 | 50.26±2 | 50.45±4.08 | C_9_H_15_N | 1194 | 29127-83-1 | -- |
| N3 | Cyclopentanecarbonitrile, 3-(1-methylethylidene)- | 1081.08±64.46 | 1048.02±49.6 | 1031±33.4 | C_9_H_13_N | 1166 | 89683-72-7 | -- |
| S2 | Dimethyl trisulfide | 40.79±1.69 | 36.4±8.95 | 36.47±4.96 | C2H_6_S_3_ | 971 | 3658-80-8 | C08372 |
| S3 | Thioanisole | 2287.17±349.56 | 1955.61±58.56 | 2075.24±52.22 | C_7_H_8_S | 1106 | 100-68-5 | -- |
| S4 | 3-(Propylthio)-1-propene | 667.01±124.44b | 2111.45±53.43a | 2219.91±35.23a | C_6_H_12_S | 871 | 27817-67-0 | -- |
| S5 | Diallyl sulfide | 60.35±15.46b | 185.5±6.85a | 197.6±10.13a | C_6_H_10_S | 861 | 592-88-1 | C08370 |
| N4 | Biuret | 505.46±6.36 | 515.41±23.67 | 491.05±17.98 | C_2_H_5_N_3_O_2_ | 1237 | 108-19-0 | C06555 |
| N5 | Dicyandiamide | 136.65±27.5b | 342.29±8.26a | 347.54±11.7a | C_2_H_4_N_4_ | 866 | 461-58-5 | -- |
| ***Nitrogen/Sulfur compounds*** |  | 5193.19±521.39b | 6613.78±227.28a | 6784.64±173.3a |  |  |  |  |
| P1 | Propofol | 4.81±0.42b | 5.91±0.15a | 5.84±0.49a | C_12_H_18_O | 1364 | 2078-54-8 | C07523 |
| P2 | [1,1′-Biphenyl]-2-ol | 1.81±0.26c | 7.04±0.61a | 5.71±0.34b | C_12_H_10_O | 1507 | 90-43-7 | C02499 |
| P3 | 4-Methyl-2-nitrophenol | 124.39±1.17 | 128.16±3.83 | 121.49±5.25 | C_7_H_7_NO_3_ | 1250 | 119-33-5 | -- |
| P4 | 4-Ethylguaiacol | 7.97±1.06 | 11.85±0.79 | 12.32±3.48 | C_9_H_12_O_2_ | 1282 | 2785-89-9 | -- |
| P5 | 8,9-Dehydrothymol | 3.01±0.23 | 3.15±0.14 | 3.29±0.33 | C_10_H_12_O | 1221 | 18612-99-2 | -- |
| P6 | 4-Propylphenol | 2623.59±35.32 | 2616.77±160.97 | 2510.02±104.59 | C_9_H_12_O | 1260 | 645-56-7 | C14311 |
| P7 | 2,3,6-Trimethylphenol | 2583.46±112.1a | 84.75±1.54b | 73.33±1.94b | C_9_H_12_O | 1239 | 2416-94-6 | -- |
| P8 | Guaiacol | 77.97±5.49 | 937.37±135.36 | 804.21±21.42 | C_7_H_8_O_2_ | 1089 | 32994 | C01502 |
| P9 | Phenol | 868.63±90.39ab | 694.45±160.82b | 553.31±69.26a | C_6_H_6_O | 980 | 108-95-2 | C00146 |
| ***Phenols*** |  | 4482.15±252.74 | 4203.72±131.98 | 4454.32±216.12 |  |  |  |  |
| Hh1 | 2-Bromododecane | 100.06±33.39b | 267.26±27.2a | 251.14±10.98a | C_12_H_25_Br | 1505 | 13187-99-0 | -- |
| Hh2 | Cyclopentyl bromide | 1379.74±191.7a | 183.41±7.86b | 175.14±10.99b | C_5_H_9_Br | 857 | 137-43-9 | -- |
| ***Halogenated hydrocarbons*** |  | 1479.8±159.15b | 450.67±20.83a | 426.27±21.92a |  |  |  |  |
| Am1 | Diethylpropion | 0±0 | 29.55±1.37a | 23.94±1.96b | C_13_H_19_NO | 1497 | 90-84-6 | C06954 |
| Am2 | Hordenine | 221.24±21.56 | 213.47±10.8 | 197.02±15.08 | C_10_H_15_NO | 1495 | 539-15-1 | C06199 |
| Am3 | Isopropylbenzylamine | 15.9±1.63 | 26.13±6.11 | 26.68±9.76 | C_10_H_15_N | 1226 | 102-97-6 | -- |
| Am4 | N-Methyloctylamine | 5.91±1.31b | 7.49±0.31b | 10.25±1.81a | C_9_H_21_N | 1088 | 2439-54-5 | -- |
| Am5 | (+)-1-(4-Methylphenyl)ethylamine | 3.09±0.34 | 2.64±0.26 | 3.07±0.24 | C_9_H_13_N | 1185 | 4187-38-6 | -- |
| Am6 | Benzeneacetamide | 66.03±8.42b | 121.39±3.1a | 106.07±14.87a | C_8_H_9_NO | 1402 | 103-81-1 | C02505 |
| Am7 | 1-ButanAmine, N-methyl-N-2-propenyl- | 97.83±26.23a | 68.54±7.39b | 9.88±0.58b | C_8_H_17_N | 856 | 24209-62-9 | -- |
| Am8 | N,N-Dimethylaniline | 28.72±30.11 | 22.87±3.05 | 19.65±0.14 | C_8_H_11_N | 1101 | 121-69-7 | C02846 |
| Am9 | Glutarimide | 172.37±7.95a | 156.23±4.68b | 157.98±4.27b | C_5_H_7_NO_2_ | 1154 | 1121-89-7 | C07275 |
| ***Amines*** |  | 575.95±11.37 | 586.43±21.43 | 552.84±48.12 |  |  |  |  |
| Et1 | Benzyl butyl ether | 1.88±0.32b | 8.29±1.36a | 7.52±0.71a | C_11_H_16_O | 1267 | 588-67-0 | -- |
| ***Ether*** |  | 1.88±0.32b | 8.29±1.36a | 7.52±0.71a |  |  |  |  |
| ***Total*** |  | 285856.82±6039.4a | 287686.11±11520.7a | 286029.37±12735.26a |  |  |  |  |

^a^ The different small letters indicate a significant difference (*p* < 0.05) among different KJ.

**Table S6 The significant DVMs in different KJ samples**

| SC | Code | Compounds | Concentration (μg/L)^a^ | | | CK vs LBKJ | | CK vs PBKJ | | LBKJ vs PBKJ | | Odor description^c^ |
| --- | --- | --- | --- | --- | --- | --- | --- | --- | --- | --- | --- | --- |
|  |  |  | CK | LBKJ | PBKJ | VIP | Type | VIP | Type | VIP | Type |  |
| 1 | T12 | β-Sinensal | - | 8.75±2.28a | 9.47±1.00a | 1.413 | ↑^b^ | 1.478 | ↑ | - | - | orange sweet juicy |
| 1 | H13 | (5Z,13Z)-tricyclo[8.6.0.02,9]hexadeca-5,13-diene | - | 15.35±2.21a | 18.45±1.81a | 1.457 | ↑ | 1.474 | ↑ | - | - | - |
| 1 | H27 | 1-Tridecene | 1.32±0.89b | 5.64±0.41a | 7.24±1.88a | 1.423 | ↑ | 1.369 | ↑ | - | - | - |
| 1 | Alc7 | 5-Undecanol | - | 28.84±3.06b | 35.46±2.71a | 1.463 | ↑ | 1.480 | ↑ | - | - | - |
| 1 | K20 | 3-(Hydroxymethyl)-2-nonanone | 8.88±0.31c | 303.65±11.73b | 333.72±1.15a | 1.475 | ↑ | 1.490 | ↑ | - | - | fresh sweet herbal |
| 1 | E65 | Ethyl phenylacetate | 5.32±0.48c | 58.45±4.09b | 75.32±0.69a | 1.471 | ↑ | 1.490 | ↑ | - | - | - |
| 1 | E72 | 1-(dimethylamino)propan-2-yl acetate | 68.83±16.54b | 319.19±21.49a | 328.45±11.05a | 1.462 | ↑ | 1.481 | ↑ | - | - | - |
| 1 | E74 | Hexyl acetate | 1.61±0.31b | 3.96±0.66a | 4.67±0.27a | 1.385 | ↑ | 1.465 | ↑ | - | - | green apple banana |
| 1 | K51 | 8-Nonen-2-one | 10.37±2.43c | 302.12±12.25b | 341.02±0.95a | 1.473 | ↑ | 1.490 | ↑ | - | - | fruity baked |
| 1 | E79 | 2-Methylbutyl acetate | 93.32±19.61b | 217.6±1.46a | 224.51±19.71a | 1.455 | ↑ | 1.441 | ↑ | - | - | over-ripe fruit |
| 1 | H53 | trans-3,4-Epoxyoctane | 874.79±192.57b | 3725.37±167.08a | 4044.03±115.55a | 1.465 | ↑ | 1.482 | ↑ | - | - | - |
| 1 | K59 | 2,2-Dimethyl-3-hexanone | 872.18±195.8b | 3799.41±169.7a | 4004.82±106.94a | 1.466 | ↑ | 1.483 | ↑ | - | - | - |
| 1 | Alc44 | 3-Heptanol | 14.09±3.93b | 56.04±5.03a | 66.3±10.48a | 1.451 | ↑ | 1.433 | ↑ | - | - | powerful herbal |
| 1 | E85 | Ethyl butyrate | - | 7.97±1.01b | 9.56±0.93a | 1.463 | ↑ | 1.484 | ↑ | - | - | pineapple cognac |
| 1 | S4 | 3-(Propylthio)-1-propene | 667.01±124.44b | 2111.45±53.43a | 2219.91±35.23a | 1.465 | ↑ | 1.482 | ↑ | - | - | alliaceous garlic |
| 1 | K66 | 2-Heptanone | - | 145.78±4.97b | 165.57±5.62a | 1.475 | ↑ | 1.486 | ↑ | - | - | fruit herbal coconut |
| 1 | S5 | Diallyl sulfide | 60.35±15.46b | 185.5±6.85a | 197.6±10.13a | 1.455 | ↑ | 1.468 | ↑ | - | - | garlic horseradish |
| 1 | Alc49 | 1-Hexanol | 2201.63±478.2b | 5565.25±129.23a | 5610.42±188.93a | 1.453 | ↑ | 1.465 | ↑ | - | - | fruity green alcoholic |
| 1 | Alc50 | (E)-2-hexen-1-ol | 813.66±201.07c | 3736.09±67.17b | 4194.94±133.53a | 1.468 | ↑ | 1.482 | ↑ | - | - | Fresh leafy unripe banana |
| 1 | N5 | Dicyandiamide | 136.65±27.5b | 342.29±8.26a | 347.54±11.7a | 1.455 | ↑ | 1.468 | ↑ | - | - | - |
| 2 | Hc29 | 1H-Pyrrolo[3,2-d]pyrimidine-2,4(3H,5H)-dione | - | 2.67±0.23a | 1.5±0.46b | 1.466 | ↑ | 1.402 | ↑ | - | - | - |
| 3 | E3 | Bornyl butyrate | - | 84.6±2.68a | 67.51±6.2b | 1.474 | ↑ | 1.477 | ↑ | - | - | herbal woody |
| 3 | E4 | Neryl isobutyrate | 6.21±0.76c | 60.93±0.88a | 47.61±2.96b | 1.474 | ↑ | 1.481 | ↑ | - | - | sweet fresh raspberry strawberry |
| 3 | E8 | 2-Furanylmethyl heptanoate | 15.76±2.84c | 228.48±2.35a | 175.55±15.16b | 1.475 | ↑ | 1.476 | ↑ | - | - | green |
| 3 | Am1 | Diethylpropion | - | 29.55±1.37a | 23.94±1.96b | 1.474 | ↑ | 1.479 | ↑ | - | - | - |
| 3 | T54 | trans-3a-cis-9a-1,2,3,3a,8,9,9a,9b-octahydro-4H-cyclopenta[def]phenanthrene | - | 113.52±2.44a | 86.19±9.44b | 1.475 | ↑ | 1.473 | ↑ | - | - | - |
| 3 | T55 | Geranyl acetate | 231.33±28.32b | 499.06±9.94a | 440.37±53.03a | 1.462 | ↑ | - | - | - | - | floral rose lavender green waxy |
| 3 | E13 | γ-Terpinyl acetate | - | 3.2±0.5a | 2.5±0.71a | 1.448 | ↑ | 1.401 | ↑ | - | - | - |
| 3 | E14 | Ethyl (E,Z)-2,4-decadienoate | - | 68.93±0.89a | 51.9±4.82b | 1.475 | ↑ | 1.477 | ↑ | - | - | pear apple tropical |
| 3 | K9 | Dihydro-α-ionone | 289.48±36.93b | 617.13±15.54a | 544.28±65.97a | 1.459 | ↑ | 1.477 | ↑ | - | - | woody floral violet raspberry |
| 3 | K15 | (±)-α-Damascone | 234.61±26.66b | 498.87±9.55a | 441.34±52.18a | 1.463 | ↑ | - | - | - | - | floral rose apple fruity |
| 3 | E21 | (+)-cis-Verbenol, acetate | 4.73±0.29c | 10.34±0.44a | 8.9±0.95b | 1.462 | ↑ | - | - | - | - | floral fruity rose tea rose peach |
| 3 | T58 | Damascenone | 229.19±27.22b | 491.57±8.9a | 434.08±51.44a | 1.464 | ↑ | - | - | - | - | apple rose honey tobacco |
| 3 | Alc2 | 1-Dodecanol | 7.59±1.46c | 112.84±4.12a | 87.52±7.13b | 1.474 | ↑ | - | - | - | - | earthy fatty honey coconut |
| 3 | Alc4 | Benzenemethanol, 4-methyl-.alpha.-(1-methyl-2-propenyl)-, (R*,R*)- | 288.91±36.74b | 617.32±13.34a | 544.07±66.47a | 1.460 | ↑ | - | - | - | - | - |
| 3 | Alc9 | 2-[(4-Chlorophenyl)amino]ethanol | 2±0.64c | 80.72±1.83a | 62.39±4.72b | 1.475 | ↑ | 1.480 | ↑ | - | - | - |
| 3 | P2 | [1,1′-Biphenyl]-2-ol | 1.81±0.26c | 7.04±0.61a | 5.71±0.34b | 1.457 | ↑ | 1.474 | ↑ | - | - | - |
| 3 | Hc9 | (±)-Massoia lactone | 8.27±1.42c | 90.39±1.61a | 74.37±6.78b | 1.476 | ↑ | 1.474 | ↑ | - | - | creamy peach herbal |
| 3 | K24 | Piperitenone oxide | - | 7.99±1.13a | 6.46±0.65b | 1.453 | ↑ | 1.474 | ↑ | - | - | herbal minty |
| 3 | T67 | Perillic acid | 3.83±1.42c | 221.85±9.4a | 175.98±15.91b | 1.475 | ↑ | 1.476 | ↑ | - | - | - |
| 3 | E55 | 2-Hydroxyethyl benzoate | 100.88±13.44b | 208.78±3.38a | 185.08±22.17a | 1.458 | ↑ | - | - | - | - | - |
| 3 | K28 | 2-Hydroxy-1-phenylbutan-1-one | 94.38±11.47b | 205.63±2.13a | 180.89±25.05a | 1.465 | ↑ | - | - | - | - | - |
| 3 | K30 | 4-Methoxyphenylacetone | 287.38±36.85b | 615.53±13.63a | 542.79±66.35a | 1.460 | ↑ | - | - | - | - | creamy licorice fennel |
| 3 | Hc19 | 1H-Pyrrolo(2,3-b)pyridine, 2-isopropyl- | 9.92±1.48b | 19.95±0.84a | 17.75±2.15a | 1.446 | ↑ | - | - | - | - | - |
| 3 | Hc20 | 2-Acetylbenzofuran | 10±1.05b | 20.11±0.67a | 17.88±2.09a | 1.459 | ↑ | - | - | - | - | Honey floral tropical |
| 3 | T102 | Perillyl alcohol | - | 25.51±0.88a | 20.56±1.08b | 1.475 | ↑ | 1.487 | ↑ | - | - | cumin cardamom floral violet |
| 3 | Ac7 | Phenoxyacetic acid | 32.2±4.84b | 69.38±2.45a | 61.05±7.34a | 1.454 | ↑ |  |  | - | - | sour sweet |
| 3 | Hc82 | 1H-Tetrazole-1,5-diamine | 25.96±4.1c | 156.15±7.84a | 128.8±10.94b | 1.469 | ↑ | 1.472 | ↑ | - | - | - |
| 4 | E1 | Phosphoric acid, dibutyl isopropenyl ester | 2.1±0.89b | 8.46±0.89a | 7.59±0.77a | 1.441 | ↑ | 1.436 | ↑ | - | - | - |
| 4 | Hh1 | 2-Bromododecane | 100.06±33.39b | 267.26±27.2a | 251.14±10.98a | 1.412 | ↑ | 1.433 | ↑ | - | - | - |
| 4 | H14 | Pentadecane | 101.32±33.66b | 267.98±27.91a | 253.5±11.53a | 1.409 | ↑ | 1.432 | ↑ | - | - | waxy |
| 4 | Ac2 | 2-Fluoro-4,5-dimethoxybenzoic acid | - | 2.08±0.06a | 1.75±0.08b | 1.474 | ↑ | 1.484 | ↑ | - | - | - |
| 4 | H23 | Tridecane | 5.3±4.96b | 85.7±9.54a | 77.39±2.73a | 1.459 | ↑ | 1.480 | ↑ | - | - | - |
| 4 | E34 | (Z)-6-Nonen-1-yl acetate | - | 44.82±2.44a | 37.49±1.38b | 1.473 | ↑ | 1.487 | ↑ | - | - | melon honeydew pear kiwi |
| 4 | E36 | Geranyl formate | - | 40.07±3.04a | 33.49±1.04b | 1.472 | ↑ | 1.487 | ↑ | - | - | fresh rose neroli tea |
| 4 | H28 | 1-Tridecyne | - | 49.69±1.95a | 42.87±0.7b | 1.474 | ↑ | 1.489 | ↑ | - | - | - |
| 4 | T60 | Menthoglycol | - | 49.04±3.43a | 43.4±0.76b | 1.472 | ↑ | 1.489 | ↑ | - | - | herbal eucalyptus minty |
| 4 | K21 | 2-Undecanone | 8.5±2.26c | 1167.82±58.37a | 1069.36±10.08b | 1.474 | ↑ | 1.490 | ↑ | - | - | fruity fatty creamy orris floral |
| 4 | Ald8 | 10-Undecenal | - | 90.6±3.97a | 77.03±2.98b | 1.474 | ↑ | 1.487 | ↑ | - | - | rose mandarin citrus |
| 4 | Alc11 | 2,6-Dimethyl-1-nonen-3-yn-5-ol | - | 44.59±2.72a | 37.41±1.58b | 1.472 | ↑ | 1.487 | ↑ | - | - | - |
| 4 | Alc12 | Ethanol, 2-(4-ethylphenoxy)- | - | 4.43±0.4a | 4.16±1.22a | 1.465 | ↑ | 1.395 | ↑ | - | - | - |
| 4 | Et1 | Benzyl butyl ether | 1.88±0.32b | 8.29±1.36a | 7.52±0.71a | 1.423 | ↑ | 1.462 | ↑ | - | - | floral rose |
| 4 | Hc23 | Whiskey lactone | - | 16.77±0.95a | 14.8±1.01b | 1.474 | ↑ | 1.484 | ↑ | - | - | coconut toasted nutty |
| 4 | Ald17 | 2,4-Decadienal, (2E,4Z)- | 0.8±0.01c | 48.75±2.88a | 42.16±0.66b | 1.473 | ↑ | 1.489 | ↑ | - | - | fried fatty geranium |
| 4 | S1 | Benzyl isothiocyanate | 1.76±0.4b | 5.77±1.1a | 5.58±0.19a | 1.387 | ↑ | 1.471 | ↑ | - | - | mild watercress horseradish |
| 4 | Ar19 | 2-Methylnaphthalene | - | 3.93±0.12b | 3.64±0.07a | 1.474 | ↑ | 1.488 | ↑ | - | - | sweet floral woody |
| 4 | E80 | Isoamyl acetate | 97.49±23.44b | 250.69±27.06a | 245.97±18.68a | 1.433 | ↑ | 1.456 | ↑ | - | - | fruity banana |
| 5 | H21 | 2,6-Dimethylundecane | 35.1±4.64a | 11.23±1.78b | 11.42±0.5b | 1.442 | ↓ | 1.457 | ↓ | - | - | - |
| 5 | E37 | Phenyl propyl carbonate | 7.7±0.13 | - | - | 1.476 | ↓ | 1.490 | ↓ | - | - | - |
| 5 | E46 | 2-Methylbutyl 2-methylbutanoate | 216.43±21.51a | 29.83±6.3b | 33.96±0.86b | 1.470 | ↓ | 1.478 | ↓ | - | - | herbal cheese apple green |
| 5 | E63 | Heptyl acetate | 207.61±19.82a | 24.81±1.84b | 27.84±1.38b | 1.472 | ↓ | 1.480 | ↓ | - | - | ripe fruit pear apricot |
| 5 | Alc19 | 3,7-Dimethylocta-1,5,7-trien-3-ol | 121.29±10.46a | 42.34±1.53b | 43.21±0.62b | 1.467 | ↓ | 1.474 | ↓ | - | - | mouldy |
| 5 | Alc20 | 3,7-Dimethyl-1,5(E),7-octatrien-3-ol | 121.88±10.89a | 45.52±1.82b | 48.42±0.96b | 1.465 | ↓ | 1.470 | ↓ | - | - | sweet tropical ocimene |
| 5 | Hh2 | Cyclopentyl bromide | 1379.74±191.7a | 183.41±7.86b | 175.14±10.99b | 1.453 | ↓ | 1.465 | ↓ | - | - | - |
| 5 | Ar21 | Butylbenzene | 29.44±6.26a | 8.73±0.24b | 10±0.55b | 1.400 | ↓ |  |  | - | - | - |
| 5 | H52 | Hexane, 3-ethyl-2-methyl- | 655.77±97.53a | 99.27±15.66b | 75.18±10.46b | 1.441 | ↓ | 1.460 | ↓ | - | - | - |
| 5 | Alc38 | 2,6-Dimethylcyclohexanol | 117.88±8.24a | 36.63±2.47b | 35.84±0.72b | 1.472 | ↓ | 1.481 | ↓ | - | - | - |
| 5 | Am7 | 1-ButanAmine, N-methyl-N-2-propenyl- | 97.83±26.23a | 68.54±7.39b | 9.88±0.58b | 1.458 | ↓ | 1.473 | ↓ | - | - | - |
| 5 | Ald36 | Phenylacetaldehyde | 28.71±5.35a | 8.75±0.48b | 10.1±0.64b | 1.417 | ↓ | 1.415 | ↓ | - | - | hyacinth honey cocoa |
| 5 | H56 | 2-Hexene, 6-methoxy-, (2E)- | 349.2±23.63a | 2.27±0.17b | - | 1.471 | ↓ | 1.484 | ↓ | 1.561 | ↓ |  |
| 5 | E86 | Methyl 3-methyl-2-butenoate | 38.37±5.33a | 5.76±0.27b | 5.13±0.42b | 1.453 | ↓ | 1.464 | ↓ | - | - | - |
| 5 | K67 | 2,2,3-Trimethyl cyclobutanone | 451.3±62.13a | 71.97±2.39b | 61.11±3.91b | 1.451 | ↓ | 1.464 | ↓ | - | - | - |
| 5 | Ald40 | Hexanal | 1830.49±129.87a | 12.4±0.82b | 8.42±1.32b | 1.470 | ↓ | 1.484 | ↓ | - | - | fatty leafy fruity sweaty |
| 5 | K68 | 2-Hexanone | 243.13±18.37 | - | - | 1.469 | ↓ | 1.483 | ↓ | - | - | fruity meaty buttery |
| 5 | K69 | Mesityl oxide | 56.25±3.59a | 1.58±0.4b | 1.58±0.07b | 1.470 | ↓ | 1.484 | ↓ | - | - | pungent earthy vegetable |
| 5 | Ald41 | (Z)-3-Hexenal | 58.03±5.81a | 4.95±0.17b | 3.79±0.53b | 1.465 | ↓ | 1.477 | ↓ | - | - | green fatty grassy fruity apple |
| 5 | K70 | 4-Hexen-3-one | 1382.32±195.52a | 189.59±9.75b | 175.1±10.94b | 1.452 | ↓ | 1.464 | ↓ | - | - | pungent tropical metallic |
| 5 | Ald42 | trans-2-Hexenal | 1035.22±70.03a | 412.32±81.41b | 421.77±55.85b | 1.446 | ↓ | 1.467 | ↓ | - | - | green banana cheesy |
| 5 | Hc86 | 3-Furancarboxaldehyde | 64.47±14.61a | 8.92±1.01b | 12.53±1.01b | 1.418 | ↓ | 1.416 | ↓ | - | - | - |
| 6 | E6 | Methyl laurate | 4.36±0.68a | 1.51±0.14b | 2.22±0.77b | 1.409 | ↓ | - | - | - | - | creamy coconut mushroom |
| 6 | Ald2 | (E)-2-Dodecenal | 3.05±0.6a | 0.87±0.18b | 1.23±0.26b | 1.393 | ↓ | 1.374 | ↓ | - | - | citrus mandarin |
| 6 | H51 | β-Thujene | 5.04±1.04a | 2.3±0.1b | 3.04±0.2b | 1.351 | ↓ | 1.395 | ↓ | - | - | - |
| 6 | Ald25 | 3-Ethylbenzaldehyde | 115.03±4.69a | 3.76±0.29b | 1.87±0.13b | 1.450 | ↓ | - | - | - | - | - |
| 6 | Ar33 | 1-Ethyl-3-methylbenzene | 138.55±17.28a | 60.77±3.96c | 82.28±4.53b | 1.434 | ↓ | - | - | - | - | - |
| 6 | Ar34 | 1-Ethyl-2-methylbenzene | 145.68±22.26a | 66.23±5.53b | 80.26±3.6 | 1.404 | ↓ | - | - | - | - | - |
| 6 | Ar35 | Mesitylene | 145.07±22.1a | 65.83±5.02b | 80.29±3.56b | 1.406 | ↓ | - | - | - | - | - |
| 6 | Ald37 | 4-Heptenal | 7.36±1.14a | 3.64±0.4b | 4.37±0.49b | 1.403 | ↓ | - | - | - | - | - |
| 6 | Ald39 | Benzaldehyde | 126.28±11.35a | 56.53±6.62b | 67.28±4.15b | 1.448 | ↓ | - | - | - | - | sharp sweet bitter almond |
| 6 | Hc87 | 4H-Pyran-4-one | 6.08±1.11a | 2.89±0.36b | 3.52±0.37b | 1.356 | ↓ | - | - | - | - | - |
| 7 | E28 | Nonyl acetate | - | 6.19±0.4b | 10.12±1.06a | 1.470 | ↑ | 1.475 | ↑ | - | - | green tropical fruity |
| 7 | H31 | 2,2,4,6,6-Pentamethylheptane | 7.68±0.36c | 51.78±3.9b | 119.11±13.78a | 1.465 | ↑ | 1.483 | ↑ | 1.523 | ↑ | - |
| 7 | Ald6 | Undecanal | 1.7±1.4c | 17.33±1.92b | 24.2±0.8a | 1.450 | ↑ | 1.485 | ↑ | - | - | floral citrus green fatty |
| 7 | T62 | Diosphenol | 1.14±0.35c | 9.65±0.8b | 14.99±1.24a | 1.463 | ↑ | 1.476 | ↑ | - | - | minty herbal woody |
| 7 | Alc27 | Cyclooctanemethanol | 4.5±2.66c | 14.62±3.34b | 24.83±1.59a | 1.311 | ↑ | 1.471 | ↑ | - | - | - |
| 7 | Ar23 | Durene | 2.32±0.4b | 4.15±0.69a | 5.01±0.77a | - | - | 1.388 | ↑ | - | - | rancid sweet |
| 7 | Alc32 | 1-Octanol | 10.46±4.79c | 27.22±5.6b | 39.41±4.2a | 1.306 | ↑ | 1.453 | ↑ | - | - | waxy green orange rose mushroom |
| 7 | K58 | 2-Octanone | - | 11.34±0.9b | 26.08±3.42a | 1.467 | ↑ | 1.482 | ↑ | 1.511 | ↑ | natural woody herbal |
| 8 | K46 | 2,5-Octanedione | 7.48±3.32b | 5.77±1.52b | 14.77±3.06a | - | - | - | - | 1.437 | ↑ | - |
| 8 | Alc37 | 1-Octen-3-ol | 43.24±20.07b | 66.73±14.26b | 179.43±33.56a | - | - | 1.429 | ↑ | 1.471 | ↑ | mushroom earthy green oily |

^a^ The different small letters indicate a significant difference (*p* < 0.05) among different KJ.

^b^ ↑, up-regulated DVM; ↓, down-regulated DVM.

^c^ Odor descriptions were taken Flavornet and human odor space (Acree, & Arn, 2004) and The Good Scents Company Information System (2021).

**Supplementary Materials**

**The extraction, detection, identification, and quantification of VOCs**

One mL of the KJ sample was transferred immediately to a 20 mL head-space vial (Agilent, Palo Alto, CA, USA), containing NaCl saturated solution, to inhibit any enzyme reaction. Subsequently, 10 μL of 50 μg/mL 3-Hexanone-2,2,4,4-d4 solution was added to the vials as a stable isotope internal standard and sealed using crimp-top caps with TFE-silicone headspace septa (Agilent). Fully automatic headspace-solid phase microextraction (HS-SPME) technology was used to extract samples for gas chromatography-mass spectrometry (GC-MS) analysis. At the time of SPME analysis, each vial was balanced at 60°C for 5 min, then a 120 μm DVB/CWR/PDMS fiber (Agilent) after aging (250℃, 5 min) was exposed to the headspace of the sample for 15 min at 100°C.

After sampling, desorption of the VOCs from the fiber coating was carried out in the injection port of the GC apparatus (Model 8890, Agilent) at 250°C for 5 min in the splitless mode. The identification and quantification of VOCs were carried out using an Agilent Model 8890 GC and a 7000D mass spectrometer (Agilent), equipped with a 30 m × 0.25 mm × 0.25 μm DB-5MS (5% phenyl-polymethylsiloxane) capillary column. Helium was used as the carrier gas at a linear velocity of 1.2 mL/min. The injector temperature was kept at 250°C and the detector at 280°C. The temperature rise procedure was: the initial temperature of 40°C, hold for 3.5 min, increasing at 10°C/min to 100°C, at 7°C/min to 180°C, at 25°C/min to 280°C, hold for 5 min. MS was recorded in electron impact (EI) ionization mode at 70 eV. The quadrupole mass detector, ion source and transfer line temperatures were set, respectively, at 150, 230 and 280°C. The MS was selected ion monitoring (SIM) mode was used for the identification and quantification of analytes. Qualitative analysis of the raw data obtained by GC-MS is carried out through Qualitative Analysis Workflows B.08.00 software, and the quantitative data is standardized based on stable isotope internal standard.
